# Supplementary material for: Development of Combretastatin A-4 Analogues as Potential Anticancer Agents with Improved Aqueous Solubility
Source: Molecules. 2023 Feb 10;28(4):1717. doi: 10.3390/molecules28041717 (PMC9963121; doi:10.3390/molecules28041717)

Supplementary data

Development of Combretastatin A-4 Analogues as Potential Anticancer Agents with Improved Aqueous Solubility

CONTENTS

1. Experimental section ······ 2

1.1. Reagents and instruments 2

*1. 2. General procedure for the synthesis of compounds **9a-d** 2*

1. 3. Procedure for the synthesis of 2-methyl-4-nitrobenzaldehyde 2

*1. 4. General procedure for the synthesis of compounds **12a-d**, **13-20** 3*

1. 5. Solubility assessment 4

1. 6. Plasma stability assessment 5

1. 7. The drug-plasma protein binding rate assessment 6

2. Identification data of compounds ······ 6

3. Refenences ······ 22

4. The spectra for the new compounds synthetized and the mass spectra ······ 23

4. 1. ^{1}H NMR 23

4. 2. ^{13}C NMR 34

4. 3. HRMS 45

1. Experimental section

1.1. Reagents and instruments

All the chemicals used in this study were purchased from Aladdin, all reagents were obtained commercially and used were of analytical grade and THF were dehydrated by 4Å molecular sieve. Melting points were determined with the SGWX-4 melting-point measurement apparatus and were uncorrected. The NMR spectra were recorded on a Bruker AV-300 spectrometer in DMSO-d6 using tetramethylsilane as the internal standard, chemical shifts were recorded as δ (ppm). High resolution mass spectra were obtained using MALDI-TOF/TOF mass spectrometer (Bruker Dartonik, Germany). Flash chromatography was carried out on silica gel (200–300 mesh), and chromatographic solvent proportions are expressed on a volume: volume basis.

1.2. General procedure for the synthesis of compounds 9a-d

Scheme 1. Synthesis of compounds **9a-d** [1-5]

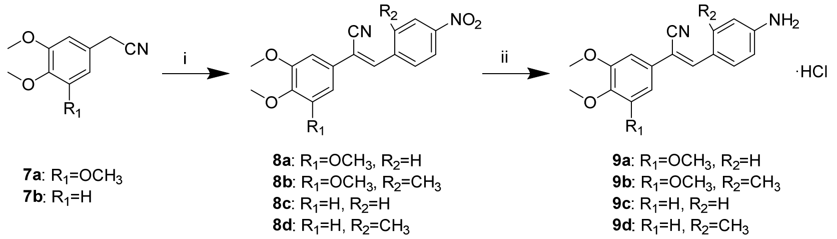

Reagents and conditions: (i) 4-Nitrobenzaldehyde or 2-methyl-4-nitrobenzaldehyde, EtOH, 5% NaOH, r.t, 0.5 h. (ii) $SnCl_2$, EtOH, 80°C, 0.5 h.

The specific synthesis steps are described in "Experimental section" in Chapter 4 of the text .

1.3. Procedure for the synthesis of 2-methyl-4-nitrobenzaldehyde

Scheme 2. Synthesis of the intermediate 2-methyl-4-nitrobenzaldehyde

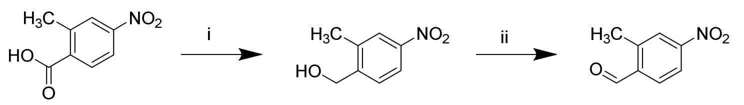

Reagents and conditions: (i) Borane dimethyl sulfide complex, THF, 70°C, 3 h. (ii) PCC, DCM, r.t., 2 h.

BH_3·DMS (10 mmol) was added dropwise to a stirred solution of nitrobenzoic acid (5 mmol) in THF (25 mL) at r.t. The solution was heated at reflux temperature for 3 h and cooled to 20°C. The reaction was quenched carefully with MeOH:H_2O (1:1, 10 mL), the mixture stirred for 10 min. HCl (5 mol/L) 5 mL was added and the mixture heated at 50°C for 30 min. The solvent was removed under reduced pressure, the residue partitioned between EtOAc and water. The organic fraction was dried, the solvent removed under reduced pressure to give (2-methyl-4-nitrophenyl)methanol [6]. Then, to a stirred CH_2Cl_2 solution (200 mL) containing pyridinium chlorochromate 2.06 g (9.56 mmol) and anhydrous magnesium sulfate 3.50 g, 1.41 g of (2-methyl-4-nitrophenyl)methanol (4.79 mmol) was added. The mixture was stirred for 2 h at ambient temperature. The organic solution was washed with water (100 mL × 3) and brine, dried over anhydrous sodium sulfate, and filtered. The filtrate was evaporated to dryness. The residue was purified on a silica gel column (PE:EA = 9:1) to afford the compound 2-methyl-4-nitrobenzaldehyde (0.73 g, 92% yield).

1.4. General procedure for the synthesis of compounds 12a-d, 13-20.

Scheme 3. Synthesis of compounds **12a-d**, **13-20** [7, 8].

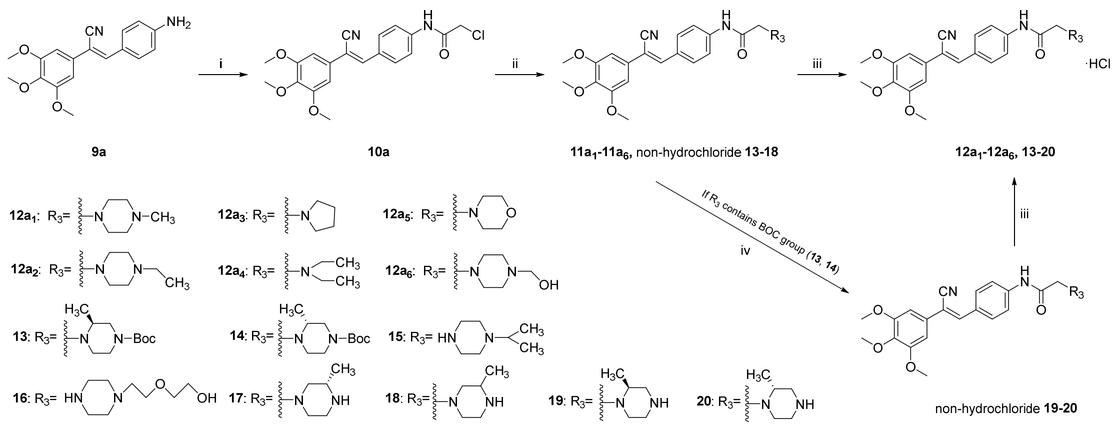

Reagents and conditions: (i) Chloroacetyl chloride, THF, tetraethylammonium hydroxide (TEA), 0°C, 2-3 h. (ii) CH_3CN, R_3, KI, K_2CO_3, 80 °C, 3 h. (iii) Hydrochloric acid, THF, rt, 8 h. (iv) 20% TFA, DCM, rt, 3 h and then hydrochloric acid, THF, rt, 8 h.

The specific synthesis steps are described in "Experimental section" in Chapter 4 of the text.

1.5. Solubility Assessment

The aqueous solubility was determined as previously reported using the high throughput solubility screens procedure published by Bruno Bard et al [9. 10]. Put simply, a certain amount of powdered compound was placed in a 2 ml centrifuge tube and mixed with an appropriate volume of aqueous phosphate buffer (0.01 M, pH 7.4) to make the whole system supersaturated. The mixtures were sonicated (KQ5200DE numerical control ultrasonic cleaner, Kunshan Ultrasonic Instrument Co., Ltd., China) for 20 min. After sonication, the mixture was allowed to equilibrate at room temperature (22 ± 2°C) for 1.5 h with vortex shaking (1200 rpm). Then the centrifuge tube was put into the centrifuge (1-14k, sigma, Germany), centrifuged with 3000 rpm for 15 minutes, 0.22 μM filter membrane filtration supernatant, then take 150 μl filtrate and add it to 96-well UV plate, with three well for each compound. Draw 150 μL blank solution were added into 96 well-plate as blank control. The 96-well UV

plate prepared above was read on a SynergyHTX Multifunctional microplate reader (BioTek, USA) at 338 nm (compounds **12a-12d** and **15-20**) or 370 nm (compounds **9a-d**). The absorbance of the sample was corrected according to the UV response of the sample and blank solution, and the corrected UV responses value were used for solubility calculations by bring into the standard curve of the compound.

1.6. Plasma stability assessment

Prepare plasma: blood was collected from SD rats (200-250g, male and female, Animal Experimental Center of Yanbian University) into heparin tubes, and supernatant plasma was collected after centrifugation.

A certain amount of **9a** and **12a$_1$** were accurately weighed and mixed with rat plasma. After incubation at 37°C for 0 h, 6 h, 12 h, 24 h and 36 h, the plasma samples were treated with methanol and analyzed by HPLC to determine their stability in plasma.

Analytical instrument and conditions:

A HITACHI Primaide HPLC liquid chromatography system equipped was used for all analyses. Chromatographic separation was performed on a Welch Ultimate XB-C18 column (3.9 mm × 150 mm, 5 μm; Welch, China). The analytes were eluted using a gradient method with a flow rate of 1 mL/min. Mobile phase A was Ultra pure water, and mobile phase B was methanol according to the following gradient: (1). The gradient elution conditions of **9a**: 0 to 8 minutes, 55% to 67% B; 8.1 to 15 minutes, 67% to 90% B; 15.1 to 25 minutes, 55% B. (2). The gradient elution conditions of **12a$_1$**: 0 to 9 minutes, 55% to 65% B; 9.1 to 12 minutes, 65% to 75% B; 12.1 to 15

minutes, 75% to 85% B. 15.1 to 20 minutes, 90% B; 20.1 to 30 minutes, 55% B. The injection volume was 3 μL. The column temperature was set at 40°C.

1.7. The drug-plasma protein binding rate assessment

Compound **12a₁** at 90 μM concentration were mixed with rats plasma. The mixtures were subjected to equilibrium dialysis versus phosphate buffer pH 7.4 buffer at 4°C for 48 hour using equilibrium dialysis. The dialysis membrane of molecular weight cutoff 1 kDa was used. Dialysis experiments were done in triplicate. On completion of the dialysis period, absorb a certain amount of buffer and plasma respectively, and add methanol to them to treat the sample. Dialysis samples were analyzed by HPLC, The plasma protein binding rate of the compound was calculated as follows: protein binding rate / % = (plasma drug concentration inside dialysis bag − dialysate drug concentration outside dialysis bag) / plasma drug concentration inside dialysis bag × 100%.

2. Identification data of compounds

(Z)-3-(4-Aminophenyl)-2-(3,4,5-trimethoxyphenyl)acrylonitrile monohydrochloride (9a)

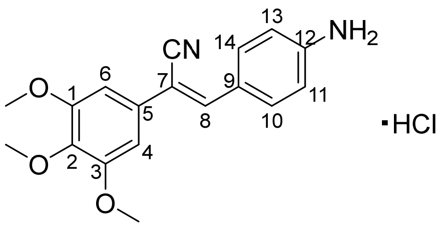

Yellow powder, mp. 203-207°C, yield 48%. ^{1}H NMR (300 MHz, DMSO-d_6): δ 7.86 (d, J = 6.0 Hz, 2H, C11, C13-H), 7.82 (s, 1H, C8-H), 7.37 (s, 3H, C12-$\underline{NH_3}^+$), 7.00 (d,

J = 6.0 Hz, 2H, C10, C14-H), 6.96 (s, 2H, C4, C6-H), 3.86 (s, 6H, C1, C3-OCH3), 3.70 (s, 3H, C2-OCH3). ^{13}C NMR (75 MHz, DMSO-d_6): δ 153.68 (s, C1, C3), 142.50 (s, C12), 138.60 (s, C2), 131.21 (s, C10, C14), 130.22 (s, C8), 128.60 (s, C5), 119.90 (s, C9, C11, C13), 118.89(s, C7-CN), 107.72 (s, C7), 103.59 (s, C4, C6), 60.62 (s, C2-OCH3), 56.57 (s, C1-OCH3, C3-OCH3). HRMS calcd. for $C_{18}H_{19}N_2O_3^+$ ([M+H]$^+$): 311.13902, found: 311.13889.

(Z)-3-(4-Amino-2-methylphenyl)-2-(3,4,5-trimethoxyphenyl)acrylonitrile monohydrochloride(9b)

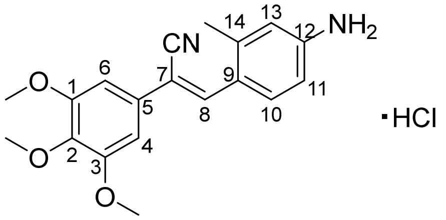

Yellow powder, mp: 224-230°C, yield 48%. ^1H NMR (300 MHz, DMSO-d_6) δ 8.02 (s, 1H, C8-H), 7.81 (d, J = 8.0 Hz, 1H, C13-H), 7.06 (s, 2H, C10, C11-H), 7.00 (s, 2H, C4, C6-H), 3.86 (s, 6H, C1, C3-OCH3), 3.71 (s, 3H, C2-OCH3), 2.38 (s, 3H, C14-CH3). ^{13}C NMR (75 MHz, DMSO-$d6$) δ 153.71 (s, C1, C3), 141.69 (s, C12), 140.11 (s, C2), 138.96 (s, C14), 130.60 (s, C8), 129.76 (s, C10), 129.71 (s, C9), 122.84 (s, C5), 119.03 (s, C7-CN), 118.29 (s, C13), 112.34 (s, C7, C11), 104.00 (s, C4, C6), 60.64 (s, C2-OCH3), 56.60 (s, C1, C3-OCH3), 20.05 (s, C14-CH3). HRMS calcd. for $C_{19}H_{21}N_2O_3^+$ ([M+H]$^+$): 325.15467, found: 325.15460.

(Z)-3-(4-Aminophenyl)-2-(3,4-dimethoxyphenyl)acrylonitrile monohydrochloride (9c)

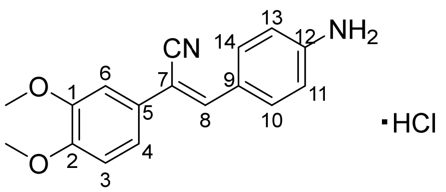

Yellow powder, mp: 214-220°C, yield 50%. ^{1}H NMR (300 MHz, DMSO-*d*$_6$) δ 7.85 (s, 1H, C8-H), 7.83 (s, 2H, C10, C14-H), 7.29 (d, *J* = 2.2 Hz, 1H, C6-H), 7.22 (dd, *J* = 8.4, 2.3 Hz, 1H, C4-H), 7.07 (s, 2H, C11, C13-H), 7.05 (s, 1H, C3-H), 3.85 (s, 3H, C2-O$\underline{CH_3}$), 3.80 (s, 3H, C1-O$\underline{CH_3}$). ^{13}C NMR (75 MHz, DMSO-*d6*) δ 150.15 (s, C1), 149.61 (s, C2), 140.58 (s, C12), 130.93 (s, C9, C10, C14), 127.03 (s, C5), 120.73 (s, C4, C7-$\underline{C}$N), 119.26 (s, C11, C13), 118.82 (s, C8), 112.30 (s, C6), 108.96 (s, C3), 108.58 (s, C7), 56.20 (s, C2-O$\underline{C}H_3$), 56.10 (s, C1-O$\underline{C}H_3$). HRMS calcd. for $C_{17}H_{17}N_2O_2^+$ ($[M+H]^+$): 281.12845, found: 281.12851.

(Z)-3-(4-Amino-2-methylphenyl)-2-(3,4-dimethoxyphenyl)acrylonitrile monohydro-chloride (9d)

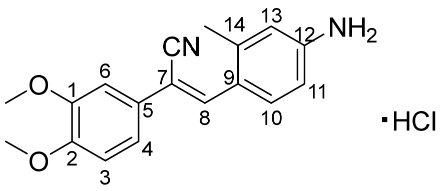

Yellow powder, mp: 236-239°C, yield 50%. ^{1}H NMR (300 MHz, DMSO-*d*$_6$) δ 7.96 (s, 1H, C8-H), 7.79 (d, *J* = 8.1 Hz, 1H, C10-H), 7.30 (s, 1H, C4-H), 7.26 (d, *J* = 8.5 Hz, 1H, C13-H), 7.06 (m, 3H, C3, C6, C11-H), 3.83 (d, *J* = 12.1 Hz, 6H, C1, C2-O$\underline{CH_3}$), 2.37 (s, 3H, C14-$\underline{CH_3}$). ^{13}C NMR (75 MHz, DMSO-*d6*) δ 150.43 (s, C1, C12), 149.61 (s, C2), 139.91 (s, C8), 139.74 (s, C14), 129.69 (s, C10), 126.64 (s, C7), 123.30 (s, C4), 119.58 (s, C5, C9, C13), 118.29 (s, C7-$\underline{C}$N), 112.72 (s, C6), 112.32 (s, C3),

109.36 (s, C11), 56.23 (s, C2-O$\underline{CH_3}$), 56.13 (s, C1-O$\underline{CH_3}$), 20.05 (s, C14-$\underline{CH_3}$). HRMS calcd. for $C_{18}H_{19}N_2O_2^+$ ($[M+H]^+$): 295.14410, found: 295.14389.

(Z)-N-(4-(2-cyano-2-(3,4,5-trimethoxyphenyl)vinyl)phenyl)-2-(4-methylpiperazin-1-yl)acetamide monohydrochloride (12a₁)

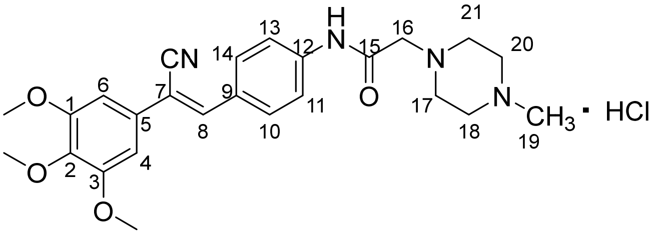

Yellow powder, mp: 230-232°C, yield 30%. ^{1}H NMR (300 MHz, DMSO-*d6*) δ11.72 (s, 1H), 11.09 (s, 1H), 8.00 (s, 1H, C8-H), 7.95 (d, 2H, C11, C13-H), 7.83(d, 2H, C10, C14-H) 7.01 (s, 2H, C4, C6-H), 4.10 (s, 2H, C16-H), 3.87 (s, 6H, C1, C3-O$\underline{CH_3}$), 3.71 (s, 3H, C2-O$\underline{CH_3}$), 3.61 (s, 4H, C17, C18, C20, C21-H), 3.40 (s, 4H, C17, C18, C20, C21-H), 2.82 (s, 3H, C19-$\underline{CH_3}$). ^{13}C NMR (75 MHz, DMSO-*d6*) δ 165.07 (s, C15), 153.70 (s, C1, C3), 142.37 (s, C12), 140.59 (s, C2), 138.81 (s, C8), 130.56 (s, C10, C14), 130.04 (s, C9), 129.68 (s, C5), 119.81 (s, C11, C13), 118.68 (s, C7-$\underline{C}$N), 109.07 (s, C7), 103.77 (s, C4, C6), 60.64 (s, C2-O$\underline{CH_3}$), 57.76 (s, C16), 56.60 (s, C1, C3-O$\underline{CH_3}$), 49.96 (d, C17, C18, C20, C21), 42.15 (s, C19). HRMS calcd. for $C_{25}H_{31}N_4O_4^+$ ($[M+H]^+$): 451.23398, found: 451.23396.

(Z)-N-(4-(2-cyano-2-(3,4,5-trimethoxyphenyl)vinyl)phenyl)-2-(4-ethylpiperazin-1-yl) acetamide monohydrochloride (12a₂)

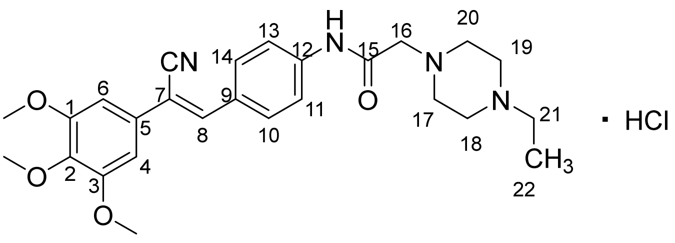

Yellow powder, mp: 232-234°C, yield 30%. ^{1}H NMR (300 MHz, DMSO-d_6) δ11.23 (s, 1H),10.78 (s, 1H), 7.99 (s, 1H, C8-H), 7.94 (d, *J* = 8.9 Hz, 2H, C11, C13-H), 7.83 (d, *J* = 8.8 Hz, 2H, C10, C14-H), 7.01 (s, 2H, C4, C6-H), 3.87 (s, 6H, C1, C3-O$\underline{CH_3}$), 3.71 (s, 3H, C2-O$\underline{CH_3}$), 3.57 (m, 2H, C16-H), 3.49 – 2.87 (m, 10H, C17, C18, C19, C20, C21-H), 1.27 (t, *J* = 7.3 Hz, 3H, C22-H). ^{13}C NMR (75 MHz, DMSO-*d6*) δ 153.70 (s, C1, C3), 142.43 (s, C12), 140.87 (s, C2), 138.77 (s, C8), 130.49 (s, C10, C14), 130.07 (s, C5), 129.40 (s, C9), 119.80 (s, C11, C13), 118.71 (s, C7-$\underline{C}$N), 108.87 (s, C7), 103.74 (s, C4, C6), 67.48 (s, C16), 60.64 (s, C2-OCH_3), 56.59 (s, C1, C3-OCH_3), 50.94 (s, C21), 49.36 (s, C17, C18, C19, C20), 9.24 (s, C22). HRMS calcd. for $C_{26}H_{33}N_4O_4^+$ ($[M+H]^+$) 465.24963, found: 465.24979.

(Z)-N-(4-(2-cyano-2-(3,4,5-trimethoxyphenyl)vinyl)phenyl)-2-(pyrrolidin-1-yl) acetamide monohydrochloride (12a$_3$)

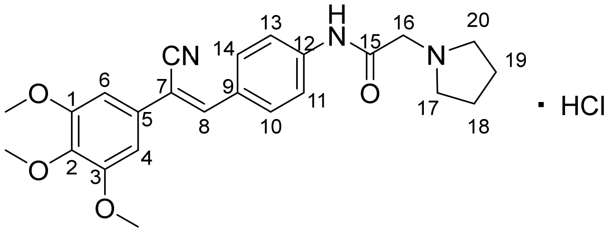

Yellow powder, mp: 238-240°C, yield 30%. ^{1}H NMR (300 MHz, DMSO-d_6) δ 11.21 (s, 1H), 10.38 (s, 1H), 7.99 (s, 1H, C8-H), 7.95(d, 2H, C11, C13-H), 7.80 (d, 2H, C10, C14-H), 7.01 (s, 2H, C4, C6-H), 4.32 (s, 2H, C16-H), 3.87 (s, 6H, C1, C3-O$\underline{CH_3}$), 3.71 (s, 3H, C2-O$\underline{CH_3}$), 3.27 (d, 4H, C17, C20-H), 1.98 (s, 4H, C18, C19-H). ^{13}C NMR (75 MHz, DMSO-*d6*) δ 164.46 (s, C15), 153.69 (s, C1, C3), 142.35 (s, C12), 140.60 (s, C2), 138.78 (s, C8), 130.58 (s, C10, C14), 130.03 (s, C5), 129.68 (s, C9), 119.70 (s, C11, C13), 118.68 (s, C7-$\underline{C}$N), 109.05 (s, C7), 103.75 (s, C4, C6), 60.62 (s,

C2-O<u>CH3</u>), 56.59 (s, C1, C3-O<u>CH3</u>), 56.16 (s, C16), 54.45 (s, C17, C20), 23.31 (s, C18, C19). HRMS calcd. for $C_{24}H_{28}N_3O_4^+$ ($[M+H]^+$): 422.20743, found: 422.20746.

(Z)-N-(4-(2-cyano-2-(3,4,5-trimethoxyphenyl)vinyl)phenyl)-2-(diethylamino) acetamide monohydrochloride (12a₄)

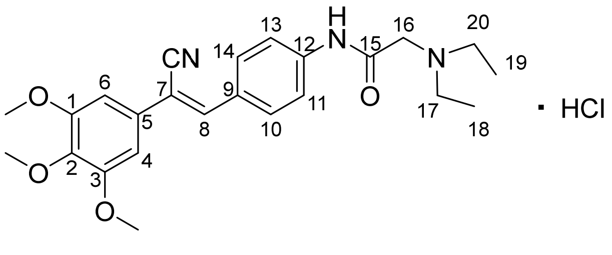

Yellow powder, mp: 230-232°C, yield 30%. ^{1}H NMR (300 MHz, DMSO-d_6) δ11.51 (s, 1H), 9.95 (s, 1H), 8.00 (s, 1H, C8-H), 7.96 (d, 2H, C11, C13-H), 7.83(d, 2H, C10, C14-H), 7.02 (s, 2H, C4, C6-H), 4.25 (s, 2H, C16-H), 3.87 (s, 6H, C1, C3-O<u>CH3</u>), 3.71 (s, 3H, C2-O<u>CH3</u>), 3.27 (s, 4H, C17, C20-H), 1.27 (t, J = 7.2 Hz, 6H, C18, C19-H). ^{13}C NMR (75 MHz, DMSO-*d6*) δ 164.27 (s, C15), 153.69 (s, C1, C3), 142.33 (s, C12), 140.52 (s, C2), 138.79 (s, C8), 130.55 (s, C10, C14), 130.01 (s, C5), 129.85 (s, C9), 119.80 (s, C11, C13), 118.66 (s, C7-<u>C</u>N), 109.17 (s, C7), 103.76 (s, C4, C6), 60.62 (s, C2-O<u>CH3</u>), 56.59 (s, C1, C3-O<u>CH3</u>), 53.20 (s, C16), 48.89 (s, C17, C20), 9.41 (s, C18, C19). HRMS calcd. for $C_{24}H_{30}N_3O_4^+$ ($[M+H]^+$): 424.22308, found: 424.22314.

(Z)-N-(4-(2-cyano-2-(3,4,5-trimethoxyphenyl)vinyl)phenyl)-2-morpholinoacetamide monohydrochloride (12a₅)

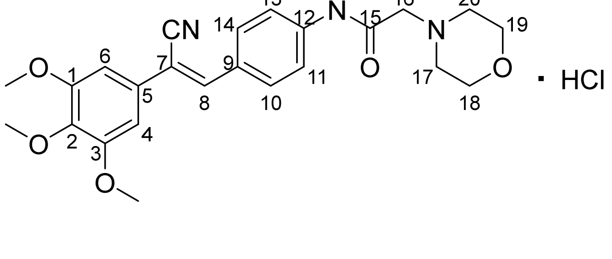

Yellow powder, mp: 140-142°C, yield 30%. ^{1}H NMR (300 MHz, DMSO-*d$_6$*): δ11.34 (s, 1H), 10.81 (s, 1H), 8.00 (s, 1H, C8-H), 7.96 (d, 2H, C11, C13-H), 7.82(d, 2H, C10, C14-H), 7.01 (s, 2H, C4, C6-H), 4.25 (s, 2H, C16-H), 3.87 (s, 6H, C1, C3-O$\underline{CH_3}$), 3.71 (s, 3H, C2-O$\underline{CH_3}$), 3.38 (s, 8H, C17, C18, C19, C20-H). ^{13}C NMR (75 MHz, DMSO-*d6*) δ 163.72 (s, C15), 153.71 (s, C1, C3), 142.34 (s, C12), 140.45 (s, C8), 138.82 (s, C2), 130.59 (s, C10, C14), 130.03 (s, C5), 129.85 (s, C9), 119.84 (s, C7-$\underline{C}$N, C11, C13), 109.19 (s, C7), 103.78 (s, C4, C6), 63.59 (s, C18, C19), 60.64 (s, C16), 57.50 (s, C2-O$\underline{CH_3}$), 56.60 (s, C1, C3-O$\underline{CH_3}$), 52.29 (s, C17, C20). HRMS calcd. for $C_{24}H_{28}N_3O_5^+$ ($[M+H]^+$): 438.20235, found: 438.20209.

(Z)-N-(4-(2-cyano-2-(3,4,5-trimethoxyphenyl)vinyl)phenyl)-2-(4-(hydroxymethyl) piperizin-1-yl)acetamide monohydrochloride (12a$_6$)

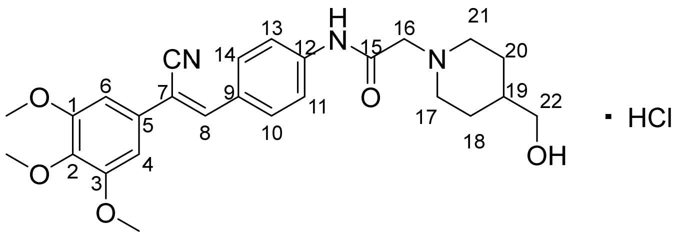

Yellow powder, mp: 94-96°C, yield 28%. ^{1}H NMR (300 MHz, DMSO-d$_6$) δ 11.27 (s, 1H), 9.93 (s, 1H), 7.99 (s, 1H, C8-H), 7.96 (d, 2H, C10, C14-H), 7.82 (d, 2H, C11, C13-H), 7.02 (s, 2H, C4, C6-H), 4.22 (s, 2H, C16-H), 3.87 (s, 6H, C1, C3-O$\underline{CH_3}$), 3.71 (s, 3H, C2-O$\underline{CH_3}$), 3.57 (d, J = 10.9 Hz, 2H, C22-H), 3.28 (d, J = 5.6 Hz, 2H, C17, C21-H), 3.12 (d, J = 11.5 Hz, 2H, C17, C21-H), 1.85 (d, J = 12.5 Hz, 2H, C18, C20-H), 1.58 (m, 3H, C18, C19, C20-H). ^{13}C NMR (75 MHz, DMSO-*d6*) δ 163.87 (s, C15), 153.69 (s, C1, C3), 142.34 (s, C12), 140.49 (s, C2), 138.80 (s, C8), 130.57 (s, C10, C14), 130.02 (s, C5), 129.82 (s, C9), 119.81 (s, C11, C13), 118.67 (s, C7-$\underline{C}$N),

109.16 (s, C7), 103.76 (s, C4, C6), 65.34 (s, C22), 60.63 (s, C2-O$\underline{CH_3}$), 56.59 (s, C1, C3-O$\underline{CH_3}$), 53.03 (s, C17, C21), 36.16 (s, C19), 26.19 (s, C18, C20). HRMS calcd. for $C_{26}H_{32}N_3O_5^+$ ([M+H]$^+$): 466.23365, found: 466.23389.

(Z)-N-(4-(2-cyano-2-(3,4,5-trimethoxyphenyl)vinyl)-3-methylphenyl)-2-(4-methylpiperazin-1-yl)acetamide monohydrochloride (12b$_1$)

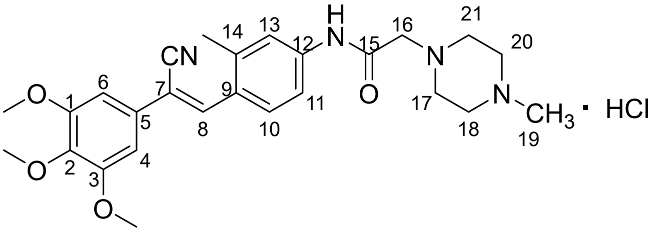

Yellow powder, mp: 232-237°C, yield 35%. ^{1}H NMR (300 MHz, DMSO-*d$_6$*) δ11.63 (s, 1H),10.89 (s, 1H), 8.06 (s, 1H, C8-H), 7.80 (d, *J* = 8.2 Hz, 1H, C10-H), 7.63 (d, *J* = 8.4 Hz, 2H, C11, C13-H), 7.01 (s, 2H, C4, C6-H), 4.08 (s, 2H, C16-H), 3.87 (s, 6H, C1, C3-O$\underline{CH_3}$), 3.71 (s, 3H, C2-O$\underline{CH_3}$), 3.59 (s, 4H, C17, C18, C20, C21-H), 3.38 (s, 4H, C17, C18, C20, C21-H), 2.82 (s, 3H, C19-H), 2.39 (s, 3H, C14-$\underline{CH_3}$). ^{13}C NMR (75 MHz, DMSO-*d6*) δ 153.71 (s, C1, C3), 141.93 (s, C9), 140.24 (s, C12), 139.29 (s, C14), 138.96 (s, C2), 129.86 (s, C5), 129.33 (s, C8), 129.09 (s, C10), 121.17 (s, C13), 118.37 (s, C7-$\underline{C}$N), 117.28 (s, C11), 112.30 (s, C7), 104.02 (s, C4, C6), 60.65 (s, C2-O$\underline{CH_3}$), 56.60 (s, C1, C3-O$\underline{CH_3}$), 49.09 (s, C19), 20.29 (s, C14-$\underline{CH_3}$). HRMS calcd. for $C_{26}H_{33}N_4O_4^+$ ([M+H]$^+$): 465.24963, found: 465.24985.

(Z)-N-(4-(2-cyano-2-(3,4,5-trimethoxyphenyl)vinyl)-3-methylphenyl)-2-(4-ethylpiperazin-1-yl)acetamide monohydrochloride (12b$_2$)

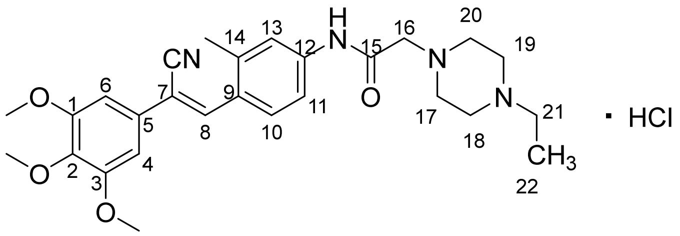

Yellow powder, mp: 226-230°C, yield 35%. ^{1}H NMR (300 MHz, DMSO-d_6) δ11.68 (s, 1H),10.95 (s, 1H), 8.06 (s, 1H, C8-H), 7.80 (d, J = 8.3 Hz, 1H, C10-H), 7.63 (d, J = 8.1Hz, 2H, C11, C13-H), 7.01 (s, 2H, C4, C6-H), 4.11 (s, 2H, C16-H), 3.87 (s, 6H, C1, C3-O$\underline{CH_3}$), 3.71 (s,3H, C2-O$\underline{CH_3}$), 3.67 (d, J = 11.5 Hz, 4H, C17, C18, C19, C20-H), 3.39 (d, 4H, C17, C18, C19, C20-H), 3.19 (d, J = 7.3 Hz, 2H, C21-H), 2.39 (s, 3H, C14-$\underline{CH_3}$), 1.28 (t, J = 7.2 Hz, 3H, C22-CH_3). ^{13}C NMR (75 MHz, DMSO-$d6$) δ 164.13 (s, C15), 153.73 (s, C1, C3), 141.93 (s, C14), 139.33 (s, C2, C12), 129.50 (s, C5, C10), 129.12 (s, C8, C9), 117.29 (s, C7-$\underline{C}$N, C11), 112.38 (s, C7, C13), 104.03 (s, C4, C6), 60.62 (s, C2-OCH_3), 56.63 (s, C1, C3-OCH_3), 50.80 (s, C17, C20), 48.95 (s, C21), 47.70 (d, C18, C19), 20.30 (s, C14-$\underline{CH_3}$), 9.18 (s, C22). HRMS calcd. for $C_{27}H_{35}N_4O_4^+$ ($[M+H]^+$): 479.26528, found: 479.26563.

(Z)-N-(4-(2-cyano-2-(3,4-dimethoxyphenyl)vinyl)phenyl)-2-(4-methylpiperazin-1-yl) acetamide monohydrochloride (12c$_1$)

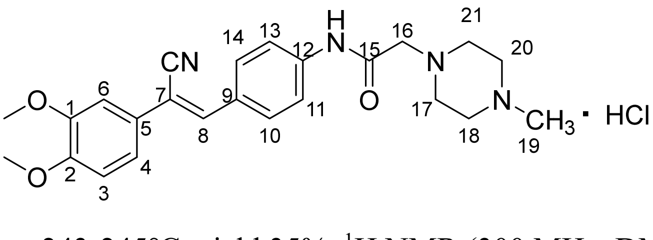

Yellow powder, mp: 243-245°C, yield 35%. ^{1}H NMR (300 MHz, DMSO-d_6) δ11.48 (s, 1H), 10.89 (s, 1H), 7.94 (s, 1H, C8-H), 7.91 (s, 2H, C11, C13-H), 7.80 (d, J = 8.8 Hz, 2H, C10, C14-H), 7.33 (d, J = 2.3 Hz, 1H, C6-H), 7.25 (dd, J = 8.4, 2.2 Hz, 1H, C4-H), 7.08 (d, J = 8.5 Hz, 1H, C3-H), 3.97 (s, 2H, C16-H), 3.86 (s, 3H, C1-O$\underline{CH_3}$),

3.81 (s, 3H, C2-O<u>CH</u>$_3$), 3.54 (s, 4H, C17, C18, C20, C21-H), 3.33 (s, 4H, C17, C18, C20, C21-H), 2.82 (s, 3H, C19-CH$_3$). ^{13}C NMR (75 MHz, DMSO-*d6*) δ 150.24 (s, C1), 149.61 (s, C2), 140.56 (s, C12), 140.30 (s, C8), 130.34 (s, C10, C14), 129.93 (s, C5), 126.97 (s, C9), 119.80 (s, C11, C13), 119.34 (s, C4), 118.76 (s, C7-<u>C</u>N), 112.30 (s, C7), 109.09 (s, C3), 109.00 (s, C6), 56.22 (s, C2-O<u>CH</u>$_3$), 56.11 (s, C1-O<u>CH</u>$_3$), 48.90 (s, C17, C18, C20, C21), 42.22 (s, C19). HRMS calcd. for $C_{24}H_{29}N_4O_3^+$ ([M+H]$^+$): 421.22342, found: 421.22333.

(Z)-N-(4-(2-cyano-2-(3,4-dimethoxyphenyl)vinyl)phenyl)-2-(4-ethylpiperazin-1-yl) acetamide monohydrochloride (12c$_2$)

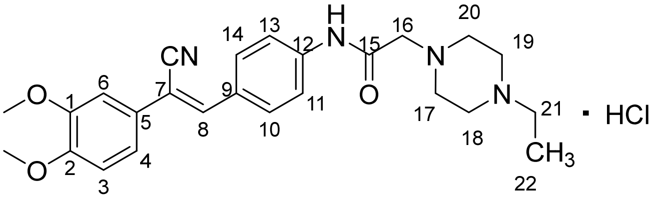

Yellow powder, mp: >250°C, yield 35%. ^{1}H NMR (300 MHz, DMSO-*d$_6$*) δ 11.39 (s, 1H), 10.90 (s, 1H), 7.94 (s, 1H, C8-H), 7.92 (s, 2H, C11, C13-H), 7.80 (d, *J* = 8.9 Hz, 2H, C10, C14-H), 7.33 (d, *J* = 2.3 Hz, 1H, C6-H), 7.25 (dd, *J* = 8.4, 2.2 Hz, 1H, C4-H), 7.08 (d, *J* = 8.6 Hz, 1H, C3-H), 4.01 (s, 2H, C16-H), 3.86 (s, 3H, C1-O<u>CH</u>$_3$), 3.81 (s, 3H, C2-O<u>CH</u>$_3$), 3.68 – 3.57 (m, 6H, C17, C20, C21-H), 3.17 (s, 4H, C18, C19-H), 1.28 (t, *J* = 7.2 Hz, 3H, C22-<u>CH</u>$_3$). ^{13}C NMR (75 MHz, DMSO-*d6*) δ 150.25 (s, C1), 149.62 (s, C2), 140.57 (s, C12), 140.32 (s, C8), 130.35 (s, C10, C14), 129.90 (s, C5), 126.97 (s, C9), 119.81 (s, C11, C13), 119.35 (s, C4), 118.76 (s, C7-<u>C</u>N), 112.32 (s, C7), 109.10 (s, C3), 109.01 (s, C6), 56.21 (s, C2-O<u>CH</u>$_3$), 56.11 (s, C1-O<u>CH</u>$_3$), 49.05 (s, C21), 9.23 (s, C22). HRMS calcd. for $C_{25}H_{31}N_4O_3^+$ ([M+H]$^+$): 435.23907, found: 435.23883.

(Z)-N-(4-(2-cyano-2-(3,4-dimethoxyphenyl)vinyl)-3-methylphenyl)-2-(4-methylpiperazin-1-yl)acetamide monohydrochloride (12d$_1$)

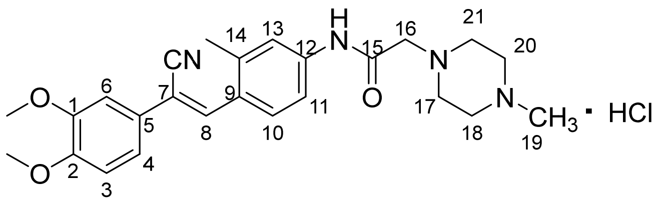

Yellow powder, mp: 234-240°C, yield 35%. ^{1}H NMR (300 MHz, DMSO-*d$_6$*) δ11.10 (s, 1H),10.52 (s, 1H), 7.98 (s, 1H, C8-H), 7.78 (d, *J* = 8.4 Hz, 1H, C10-H), 7.61 (d, *J* = 8.3 Hz, 2H, C11, C13-H), 7.32 (d, *J* = 2.2 Hz, 1H, C6-H), 7.27 (dd, *J* = 8.3, 2.3 Hz, 1H, C4-H), 7.08 (d, *J* = 8.3 Hz, 1H, C3-H), 3.86 (s, 3H, C1-O$\underline{CH_3}$), 3.82 (s, 3H, C2-O$\underline{CH_3}$), 3.76 (s, 2H, C16-H), 3.43 (d, *J* = 42.3 Hz, 4H, C17, C18, C19, C20-H), 3.20 (d, *J* = 39.1 Hz, 4H, C17, C18, C19, C20-H), 2.80 (s, 3H, C19-H), 2.39 (s, 3H, C14-$\underline{CH_3}$). ^{13}C NMR (75 MHz, DMSO-*d6*) δ 150.33 (s, C1), 149.60 (s, C2), 140.18 (s, C12), 140.05 (s, C14), 139.04 (s,C8), 129.36 (s, C5), 128.94 (s, C9), 126.84 (s, C10), 121.17 (s, C11), 119.48 (s, C4), 118.49 (s, C13), 117.27 (s, C7-$\underline{C}$N), 112.32 (s, C7), 112.11 (s, C3), 109.36 (s, C6), 56.21 (s, C2-O$\underline{CH_3}$), 56.13(s, C1-O$\underline{CH_3}$), 51.60(s, C16), 49.28 (s, C18, C20), 49.04 (s, C17, C21), 42.35(s, C19), 20.29(s, C14-CH_3). HRMS calcd. for $C_{25}H_{31}N_4O_3^+$ ([M+H]$^+$): 435.23907, found: 435.23877.

(Z)-N-(4-(2-cyano-2-(3,4-dimethoxyphenyl)vinyl)-3-methylphenyl)-2-(4-ethylpiperazin-1-yl)acetamide monohydrochloride (12d$_2$)

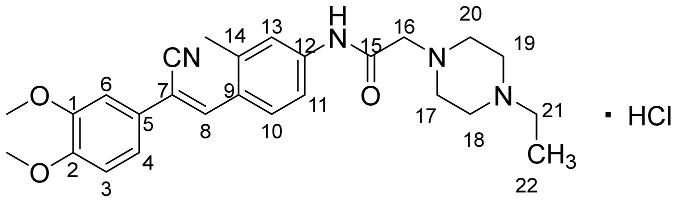

White powder, mp: 248-250°C, yield 40%. ^{1}H NMR (300 MHz, DMSO-*d6*) δ 11.51 (s, 1H), 10.84 (s, 1H), 7.99 (s, 1H, C8-H), 7.79 (d, *J* = 8.8 Hz, 1H, C10-H), 7.61 (d, *J* = 7.7 Hz, 2H, C11, C13-H), 7.32 (d, *J* = 2.2 Hz, 1H, C6-H), 7.27 (dd, *J* = 8.3, 2.2 Hz,1H, C4-H), 7.08 (d, *J* = 8.5 Hz, 1H, C3-H), 4.06 (s, 2H, C16-H), 3.86 (s, 3H, C1-O$\underline{CH_3}$), 3.82 (s, 3H, C2-O$\underline{CH_3}$), 3.63 (s, 6H, C17, C18, C19, C20, C21-H), 3.37 (s, 4H, C17, C18, C19, C20-H), 2.39 (s, 3H, C14-$\underline{CH3}$), 1.28 (t, *J* = 7.2 Hz, 3H, C22-H). ^{13}C NMR (75 MHz, DMSO-*d6*) δ 150.38 (s, C1), 149.61 (s, C2), 139.99 (s, C12), 139.88 (s, C14), 139.14 (s, C8), 129.68 (s, C5), 129.07 (s, C9), 126.79 (s, C10), 121.16 (s, C4), 119.52 (s, C13), 118.45 (s, C7-$\underline{C}$N), 117.28 (s, C11), 112.33 (s, C7), 109.38 (s, C3, C6), 56.23 (s, C2-O$\underline{CH_3}$), 56.14 (s, C1-O$\underline{CH_3}$), 49.05 (s, C21), 20.31 (s, C14-CH_3), 9.20 (s, C22). HRMS calcd for $C_{26}H_{33}N_4O_3^+$ ($[M+H]^+$): 449.25472, found: 449.25452.

(Z)-N-(4-(2-cyano-2-(3,4,5-trimethoxyphenyl)vinyl)phenyl)-2-(4-isopropylpiperazin-1-yl)acetamide monohydrochloride (15)

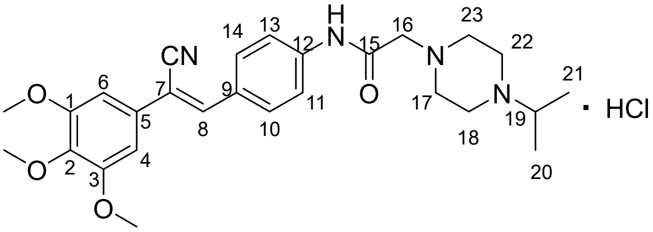

Yellow powder, m.p.: 234-236°C, yield 40%. ^{1}H NMR (300 MHz, DMSO-*d*$_6$) δ11.48 (s, 1H),10.99 (s, 1H), 7.99 (s, 1H,C8-H), 7.95 (d, *J* = 8.5 Hz, 2H, C11, C13-H), 7.82 (d, *J* = 8.6Hz, 2H, C10, C14-H), 7.01 (s, 2H, C4, C6-H), 4.08 (s, 2H, C16-H), 3.87 (s, 6H, C1, C3-OCH_3), 3.71 (s, 3H, C2-OCH_3), 3.53 (q, *J* = 32.4, 17.4 Hz, 9H, C17, C18, C19, C22, C23-H), 1.32 (d, *J* = 6.5 Hz, 6H, C20, C21-CH_3). ^{13}C NMR (75 MHz,

DMSO-*d6*) δ 164.49 (s, C15), 153.19 (s, C1, C3), 142.02 (s, C12), 139.35 (s, C2), 138.16 (s, C8), 130.38 (s, C10, C14), 129.86 (s, C5), 129.75 (s, C9), 120.09 (s, C11, C13), 118.62 (s, C7-CN), 109.09 (s, C7), 103.19 (s, C4, C6), 60.77 (s, C2-OCH3), 58.68 (s, C16), 57.90 (s, C19), 56.24 (s, C1-OCH3, C3-OCH3), 49.61 (s, C17, C23), 45.55 (s, C18, C22), 16.45 (s, C20, C21). HRMS calcd. for $C_{27}H_{35}N_4O_4^+$ ([M+H]$^+$): 479.26528, found: 479.26569.

(Z)-N-(4-(2-cyano-2-(3,4,5-trimethoxyphenyl)vinyl)phenyl)-2-(4-(2-(2-hydroxyethoxy)ethyl)piperazin-1-yl)acetamide monohydrochloride (16)

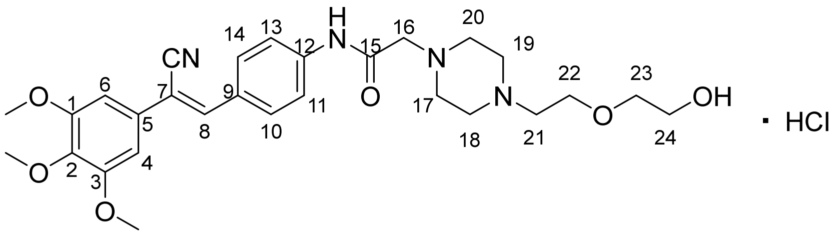

Yellow powder, mp: 240-243°C, yield 40%. ^{1}H NMR (300 MHz, DMSO-*d6*) δ11.30 (s, 1H), 10.98 (s, 1H), 7.99 (s, 1H, C8-H), 7.95 (d, *J* = 8.3 Hz, 2H, C11, C13-H), 7.82 (d, *J* = 8.4Hz, 2H, C10, C14-H), 7.01 (d, *J* = 1.2 Hz, 2H, C4, C6-H), 4.11 (s, 1H, C24-OH), 4.03 (s, 2H, C16-H), 3.88 – 3.86 (m, 6H, C1, C3-OCH3), 3.83 (s, 2H, C22), 3.71 (d, *J* = 1.0 Hz, 3H, C2-OCH3), 3.57 – 3.47 (m, 8H, C17, C18, C19, C20-H), 3.39 (s, 6H, C21, C23, C24-H). ^{13}C NMR (75 MHz, DMSO-*d6*) δ 164.68 (s, C15), 153.24 (s, C1, C3), 142.10 (s, C2, C12), 139.41 (s, C8), 138.22 (s, C5), 130.40 (s, C10, C14), 129.87 (s, C9), 120.09 (s, C11, C13), 118.64 (s, C7-CN), 109.11 (s, C7), 103.24 (s, C4, C6), 72.05 (s, C24), 63.67 (s, C22),60.77 (s, C2-OCH3), 60.29 (s, C23), 57.95 (s, C16), 56.27 (s, C1, C3-OCH3), 55.84 (s, C21), 49.41 (s, C17, C20), 49.18 (s, C18, C19). HRMS calcd. for $C_{28}H_{37}N_4O_6^+$ ([M+H]$^+$): 525.27076, found: 525.27106.

(S,Z)-N-(4-(2-cyano-2-(3,4,5-trimethoxyphenyl)vinyl)phenyl)-2-(3-methylpiperazin-1-yl)acetamide monohydrochloride (17)

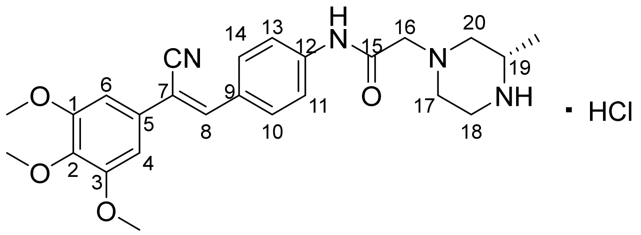

Yellow powder, mp: 196-200°C, yield 40%. ^{1}H NMR (300 MHz, DMSO-*d6*) δ10.92 (s, 1H), 9.87 (s, 1H), 9.60 (s, 1H), 7.99 (s, 1H, C8-H), 7.95 (d, *J* = 8.3 Hz, 2H, C10, C14-H), 7.82 (d, *J* = 8.4 Hz, 2H, C11, C13-H), 7.01 (d, *J* = 1.3 Hz, 2H, C4, C6-H), 4.01 (s, 2H, C16-H), 3.87 (d, *J* = 1.3 Hz, 6H, C1, C3-O$\underline{CH_3}$), 3.71 (d, *J* = 1.4 Hz, 3H, C2-O$\underline{CH_3}$), 3.61 (d, *J* = 6.6 Hz, 2H, C18-H), 3.48 (s, 2H, C17-H), 3.28 (s, 2H, C20-H), 3.07 (s, 1H, C19-H), 1.30 (d, *J* = 6.4 Hz, 3H, C19-$\underline{CH_3}$). ^{13}C NMR (75 MHz, DMSO-*d6*) δ 164.08 (s, C15), 153.69 (s, C1, C3), 142.32 (s, C12), 140.45 (s, C2), 138.79 (s, C8), 130.56 (s, C10, C14), 130.02 (s, C9), 129.90 (d, *J* = 16.8 Hz, C5), 119.83 (s, C11, C13), 118.66 (s, C7-$\underline{C}$N), 109.13 (s, C7), 103.75 (s, C4, C6), 67.48 (s, C16), 60.63 (s, C2-O$\underline{CH_3}$), 56.59 (s, C1, C3-O$\underline{CH_3}$), 54.03 (s, C17, C18), 48.53 (s, C19, C20), 15.74 (s, C19-CH_3). HRMS calcd. for $C_{25}H_{31}N_4O_4^+$ ([M+H]$^+$): 451.23398, found: 451.23395.

(Z)-N-(4-(2-cyano-2-(3,4,5-trimethoxyphenyl)vinyl)phenyl)-2-(3-methylpiperazin-1-yl)acetamide monohydrochloride (18)

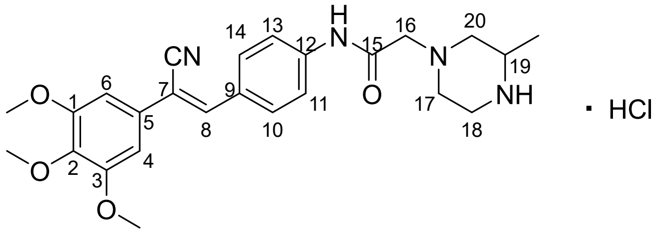

Yellow powder, mp: 195-200°C, yield 40%. ^{1}H NMR (300 MHz, DMSO-*d*$_6$) δ10.92 (s, 1H), 9.89 (s, 1H), 9.62 (s, 1H), 7.99 (s, 1H, C8-H), 7.95 (d, *J* = 8.4 Hz, 2H, C10, C14-H), 7.82 (d, *J* = 8.3 Hz, 2H, C11, C13-H), 7.01 (d, *J* = 1.4 Hz, 2H, C4, C6-H), 4.01 (s, 2H, C16-H), 3.87 (d, *J* = 1.3 Hz, 6H, C1, C3-O$\underline{CH_3}$), 3.71 (d, *J* = 1.4 Hz, 3H, C2-O$\underline{CH_3}$), 3.62 (s, 2H, C18-H), 3.49 (s, 2H, C17-H), 3.29 (s, 2H, C20-H), 3.07 (s, 1H, C19-H), 1.30 (d, *J* = 6.3 Hz, 3H, C19-$\underline{CH_3}$). ^{13}C NMR (75 MHz, DMSO-*d6*) δ164.17 (s, C15), 153.68 (s, C1, C3), 142.32 (s, C2), 140.46 (s, C8), 138.78 (s, C5), 130.56 (s, C10, C14), 130.01 (s, C9), 129.78 (s, C12), 119.82 (s, C11, C13), 118.66 (s, C7-$\underline{C}$N), 109.12 (s, C7), 103.75 (s, C4, C6), 60.63 (s, C2-O$\underline{CH_3}$), 56.59 (s, C1, C3-O$\underline{CH_3}$), 54.07 (s, C19), 48.53 (s, C17, C18, C20), 15.75 (s, C19-CH_3). HRMS calcd. for $C_{25}H_{31}N_4O_4^+$ ([M+H]$^+$): 451.23398, found: 451.23407.

(S,Z)-N-(4-(2-cyano-2-(3,4,5-trimethoxyphenyl)vinyl)phenyl)-2-(2-methylpiperazin-1-yl)acetamide monohydrochloride (19)

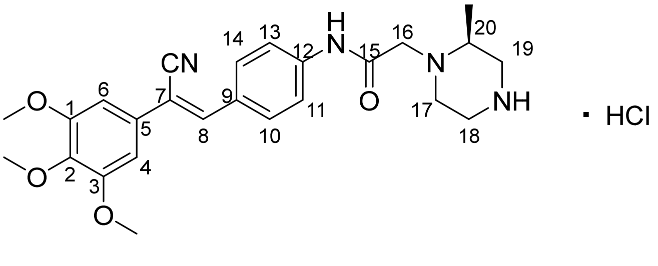

Yellow powder, mp: 200-202°C, yield 40%. ^{1}H NMR (300 MHz, DMSO-*d*$_6$) δ11.07 (s, 1H),9.74 (s, 3H), 7.99 (s, 1H, C8-H), 7.95 (d, *J* = 8.6 Hz, 2H, C11, C13-H), 7.82 (d, *J* = 8.6 Hz, 2H, C10, C14-H), 7.01 (s, 2H, C4, C6-H), 3.87 (s, 6H, C1, C3-O$\underline{CH_3}$), 3.71 (s, 3H, C2-O$\underline{CH_3}$), 3.54 (s, 7H, C17, C18, C19, C20-H), 1.30 (d, *J* = 7.1 Hz, 3H, C20-$\underline{CH_3}$). ^{13}C NMR (75 MHz, DMSO-*d6*) δ153.68 (s, C1, C3), 142.31 (s, C8), 140.42 (s, C2), 138.78 (s, C12), 130.55 (s, C10, C14), 130.00 (s, C9), 129.86 (s, C5),

119.86 (s, C11, C13), 118.65 (s, C7-CN), 109.17 (s, C7), 103.75 (s, C4, C6), 60.63 (s, C2-OCH3), 56.59 (s, C1, C3-OCH3), 55.11 (s, C16), 53.78 (s, C20), 49.04 (s, C17, C19), 45.59 (s, C18), 14.40 (s, C20-CH3). HRMS calcd. for $C_{25}H_{31}N_4O_4^+$ ($[M+H]^+$): 451.23398, found: 451.23419.

(R,Z)-N-(4-(2-cyano-2-(3,4,5-trimethoxyphenyl)vinyl)phenyl)-2-(2-methylpiperazin-1-yl)acetamide monohydrochloride (20)

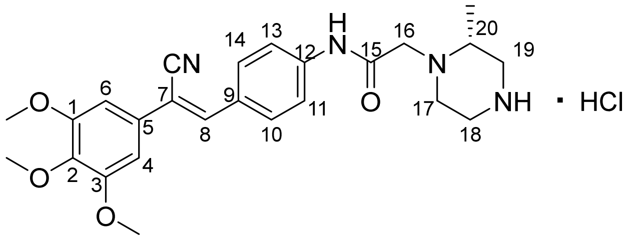

Yellow powder, mp: 200-202°C, yield 40%. ^{1}H NMR (300 MHz, DMSO-*d*$_6$) δ11.04 (s, 1H), 9.73 (s, 2H), 7.99 (s, 1H, C8-H), 7.98 – 7.92 (m, 2H, C11, C13-H), 7.82 (dd, *J* = 8.8, 1.8 Hz, 2H, C10, C14-H), 7.01 (d, *J* = 1.8 Hz, 2H, C4, C6-H), 4.12 (d, *J* = 43.9 Hz, 2H, C16-H), 3.87 (d, *J* = 1.8 Hz, 6H, C1, C3-OCH3), 3.71 (s, 3H, C2-OCH3), 3.46 (d, *J* = 13.6 Hz, 7H, C17, C18, C19, C20-H), 1.32 (d, *J* = 6.4 Hz, 3H, C20-CH3). ^{13}C NMR (75 MHz, DMSO-*d6*) δ165.13 (s, C15), 154.49 (s, C1, C3), 143.11 (s, C2, C12), 141.20 (s, C8), 139.60 (s, C5), 131.37 (s, C10, C14), 130.75 (d, *J* = 8.7 Hz, C9), 120.67 (s, C11, C13), 119.46 (s, C7-CN), 110.00 (s, C7), 104.56 (s, C4, C6), 61.44 (s, C2-OCH3), 57.40 (s, C1, C3-OCH3), 55.98 (s, C16), 54.64 (s, C20), 49.82 (s, C17, C19), 46.42 (s, C18), 15.19 (s, C20-CH3). HRMS calcd. for $C_{25}H_{31}N_4O_4^+$ ($[M+H]^+$): 451.23398, found: 451.23404.

3. Refenences

1. Srivastava, V.; Darokar, M.P.; Fatima, A.; Kumar, J.K.; Chowdhury, C.; Saxena, H. O.; Dwivedi, G.R.; Shrivastava, K.; Gupta, V.; Chattopadhyay, S.K.; Luqman, S.; Gupta, M.M.; Negi, A.S.; Khanuja, S.P. Synthesis of diverse analogues of oenostacin and their antibacterial activities. *Bioorg Med Chem*. **2007**, 15, 518–525. https://doi.org/10.1016/j.bmc.2006.09.034.

2. Chakraborty, S.; Das, U.K.; Ben-David, Y.; Milstein, D. Manganese catalyzed α-olefination of nitriles by primary alcohols. *J Am Chem Soc*. **2017**, 139, 11710–11713. https://doi.org/10.1021/jacs.7b06993.

3. Petch, D.; Anderson, R.J.; Cunningham, A.; George, S.E.; Hibbs, D.E.; Liu, R.; Mackay, S.P.; Paul, A.; Small, D.A.; Groundwater, P.W. Design and synthesis of EGFR dimerization inhibitors and evaluation of their potential in the treatment of psoriasis. *Bioorg Med Chem*. **2012**, 20, 5901–5914. https://doi.org/10.1016/j.bmc.2012.07.048.

4. Lieber, S.; Scheer, F.; Meissner, W.; Naruhn, S.; Adhikary, T.; Müller-Brüss elbach, S.; Diederich, W.E.; Müller, R. (Z)-2-(2-bromophenyl)-3-{[4-(1-methyl -piperazine)amino]phenyl}-acrylonitrile (DG172): An orally bioavailable PPAR β/δ-selective ligand with inverse agonistic properties. *J Med Chem*. **2012**, 55, 2858–2868. https://doi.org/10.1021/jm2017122.

5. Tanpure, R.P.; Strecker, T.E.; Chaplin, D.J.; Siim, B.G.; Trawick, M.L.; Pinney, K.G. Regio- and stereospecific synthesis of mono-beta-d-glucuronic acid derivatives of combretastatin A-1. *J Nat Prod*. **2010**, 73, 1093–1101. https://doi.org/10.1021/np100108e.

6. Fujikawa Y.; Urano Y.; Inoue H.; Hanaoka, K.; Kojima, H.; Terai, T.; Inoue, H.; Nagano, T. Design and synthesis of highly sensitive fluorogenic substrates for glutathione s-transferase and application for activity imaging in living cells. *J Am Chem Soc*. **2008**, 130, 14533–14543. https://doi.org/10.1021/np100108e.

7. Nurpelin, S.B.; Sinem, I.; Yusuf, O. Synthesis of new donepezil analogues and investigation of their effects on cholinesterase enzymes. *Eur J Med Chem*. **2016**, 124, 1026–1040. https://doi.org/10.1016/j.ejmech.2016.10.042.

8. Du, P.; Xu, L.; Huang, J.; Yu, K.; Zhao, R.; Gao, B.; Jiang, H.; Zhao, W.; Zhen, X.; Fu, W. Design, synthesis, and evaluation of indolebutylamines as a novel class of

selective dopamine D3 receptor ligands. *Chem Biol & Drug Des*. **2013**, 82, 326–335. https://doi.org/10.1111/cbdd.12158.

9. Bard, B.; Martel, S.; Carrupt, P.A. High throughput UV method for the estimation of thermodynamic solubility and the determination of the solubility in biorelevant media. *Eur J Pharm Sci*. **2008**, 33(3), 230–240. https://doi.org/10.1016/j.ejps.2007.12.002.

10. Chen, T.M.; Shen, H.; Zhu, C. Evaluation of a method for high throughput solubility determination using a multi-wavelength UV plate reader. *Comb Chem High Throughput Screen*. **2002**, 5, 575–581. https://doi.org/10.2174/1386207023330075.

4. The spectra for the new compounds synthetized and the mass spectra

4.1. ^{1}H NMR

9a.

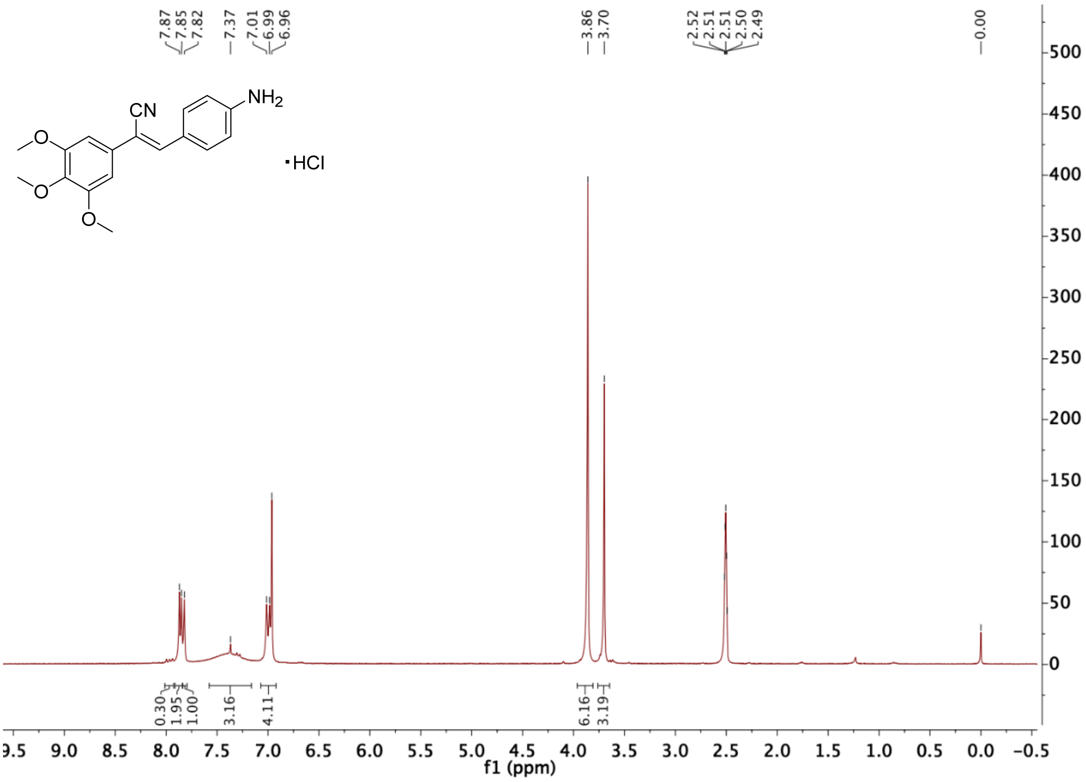

9b.

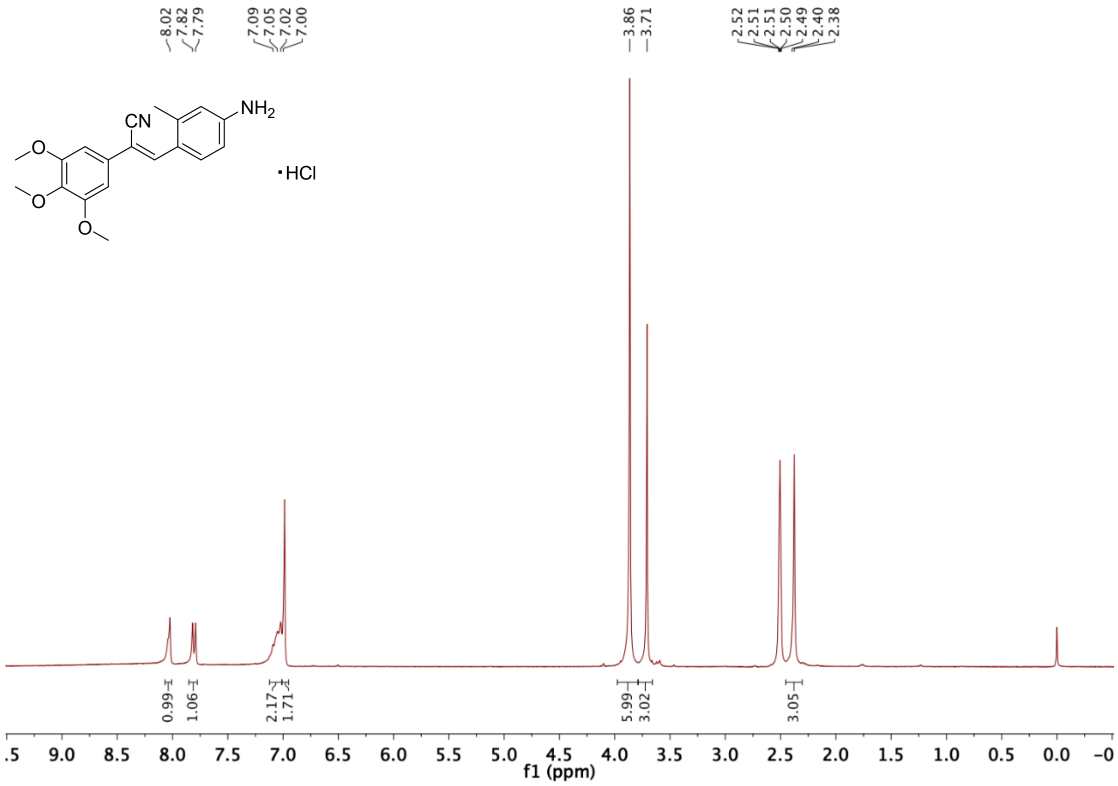
8.02
7.82
7.79
7.09
7.05
7.02
7.00
3.86
3.71
2.52
2.51
2.51
2.50
2.49
2.40
2.38
CN
NH2
•HCl
0.99
1.06
2.17
1.71
5.99
3.02
3.05
f1 (ppm)

9c.

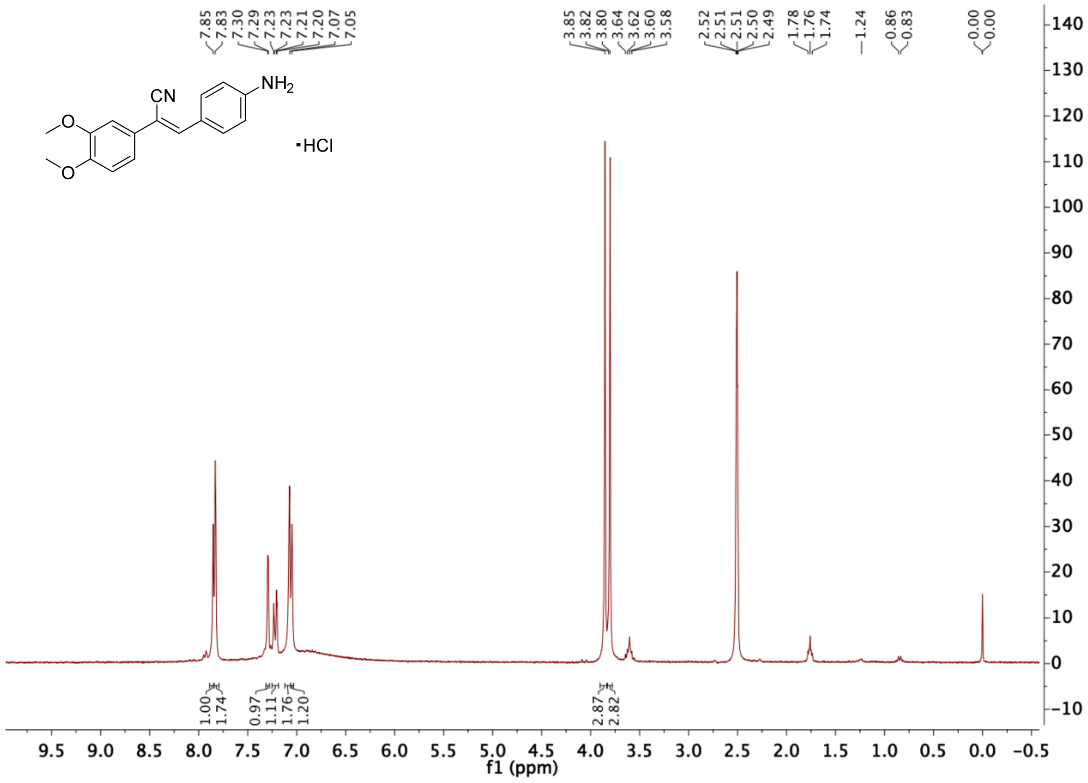
7.85
7.83
7.30
7.29
7.23
7.23
7.21
7.20
7.07
7.05
3.85
3.82
3.80
3.64
3.62
3.60
3.58
2.52
2.51
2.51
2.50
2.49
1.78
1.76
1.74
1.24
0.86
0.83
0.00
0.00
CN
NH2
•HCl
1.00
1.74
0.97
1.11
1.76
1.20
2.87
2.82
f1 (ppm)

9d.

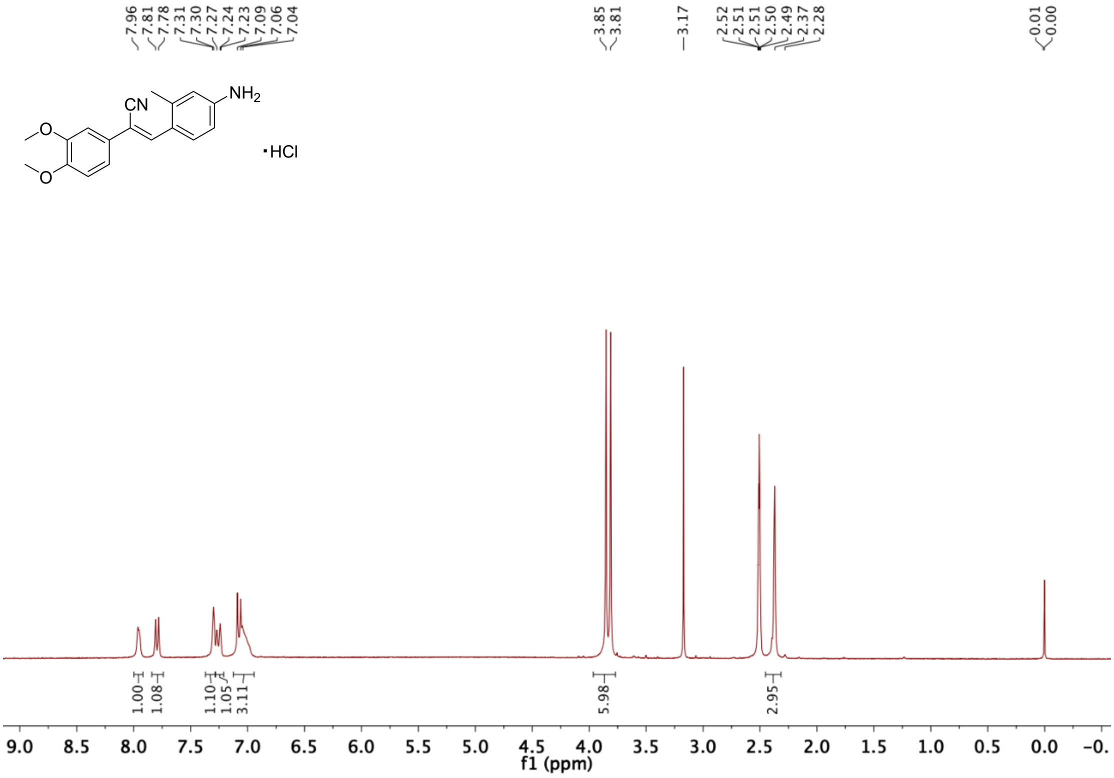
7.96
7.81
7.78
7.31
7.30
7.27
7.24
7.23
7.09
7.06
7.04
3.85
3.81
3.17
2.52
2.51
2.51
2.50
2.49
2.37
2.28
0.01
0.00
CN
NH2
O
O
·HCl
1.00
1.08
1.10
1.05
3.11
5.98
2.95
9.0 8.5 8.0 7.5 7.0 6.5 6.0 5.5 5.0 4.5 4.0 3.5 3.0 2.5 2.0 1.5 1.0 0.5 0.0 -0.
f1 (ppm)

12a$_1$.

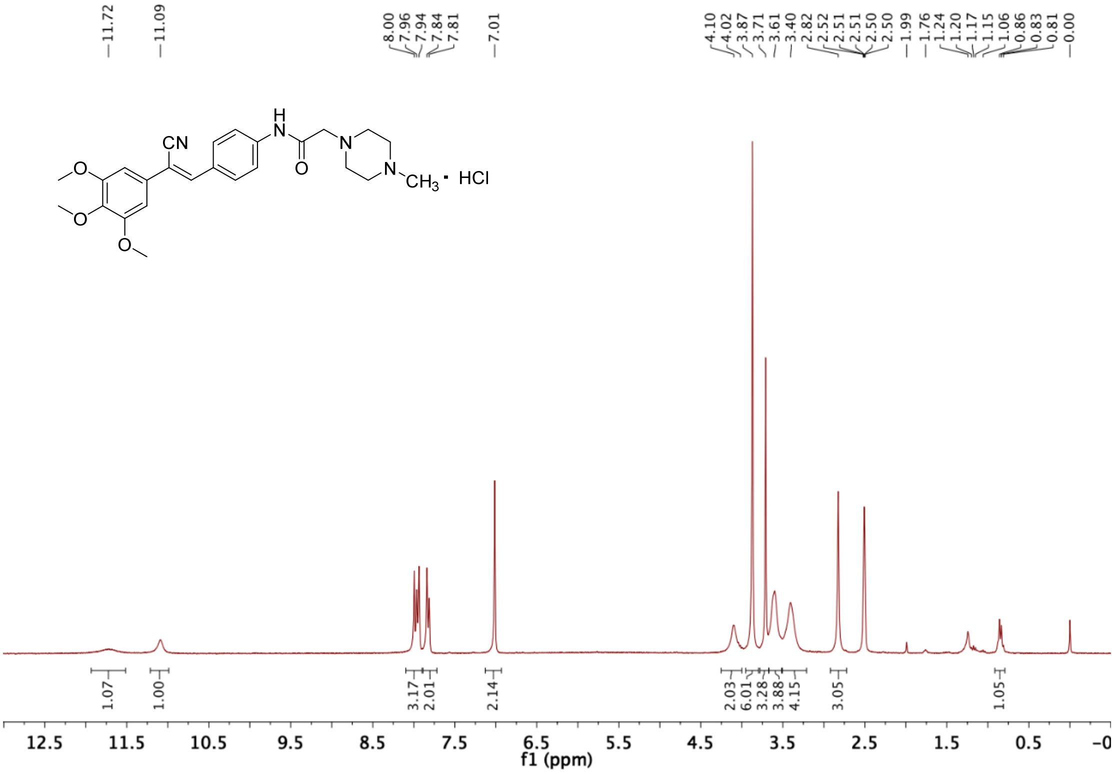
11.72
11.09
8.00
7.96
7.94
7.84
7.81
7.01
4.10
4.02
3.87
3.71
3.61
3.40
2.82
2.52
2.51
2.51
2.50
2.50
1.99
1.76
1.24
1.20
1.17
1.15
1.06
0.86
0.83
0.81
0.00
CN
H
N
O
N
N
CH3 · HCl
O
O
O
1.07
1.00
3.17
2.01
2.14
2.03
6.01
3.28
3.88
4.15
3.05
1.05
12.5 11.5 10.5 9.5 8.5 7.5 6.5 5.5 4.5 3.5 2.5 1.5 0.5 -0
f1 (ppm)

12a$_2$.

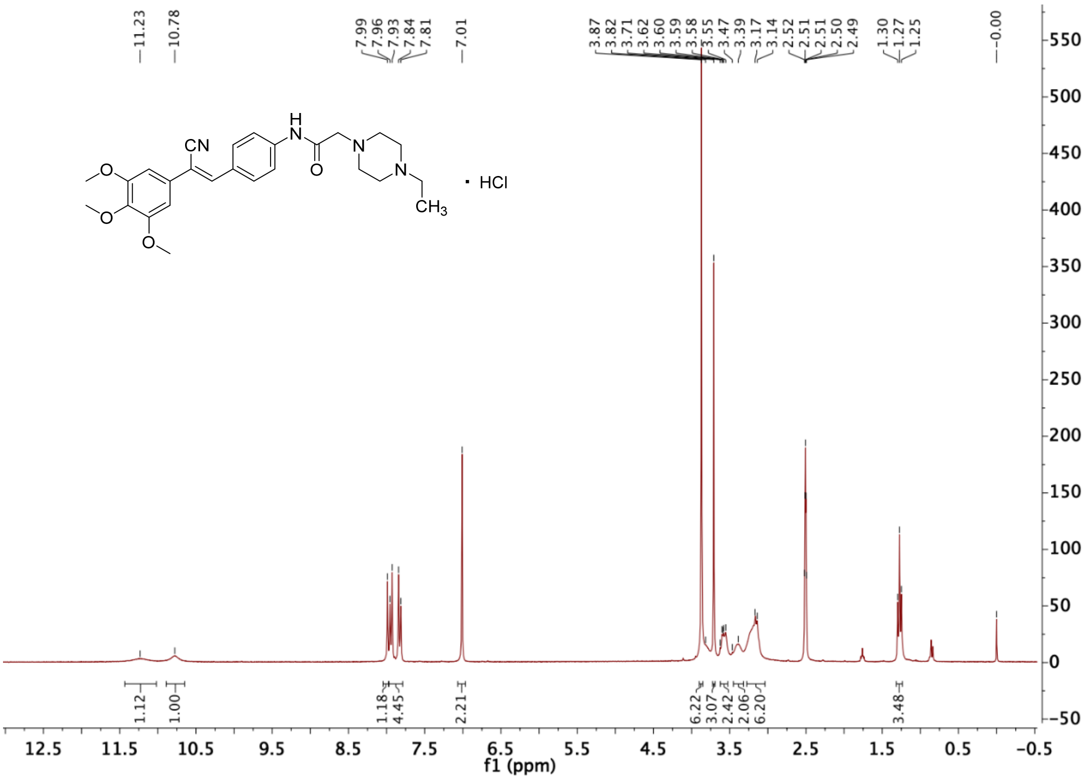

12a$_3$.

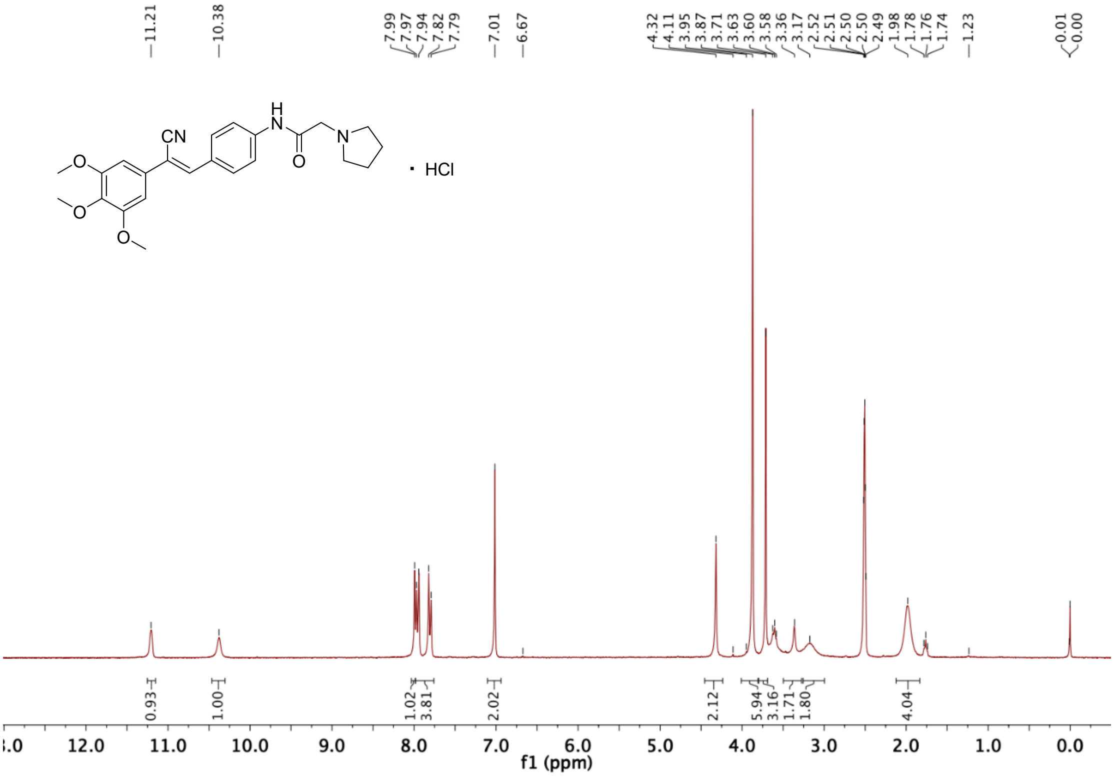

12a₄.

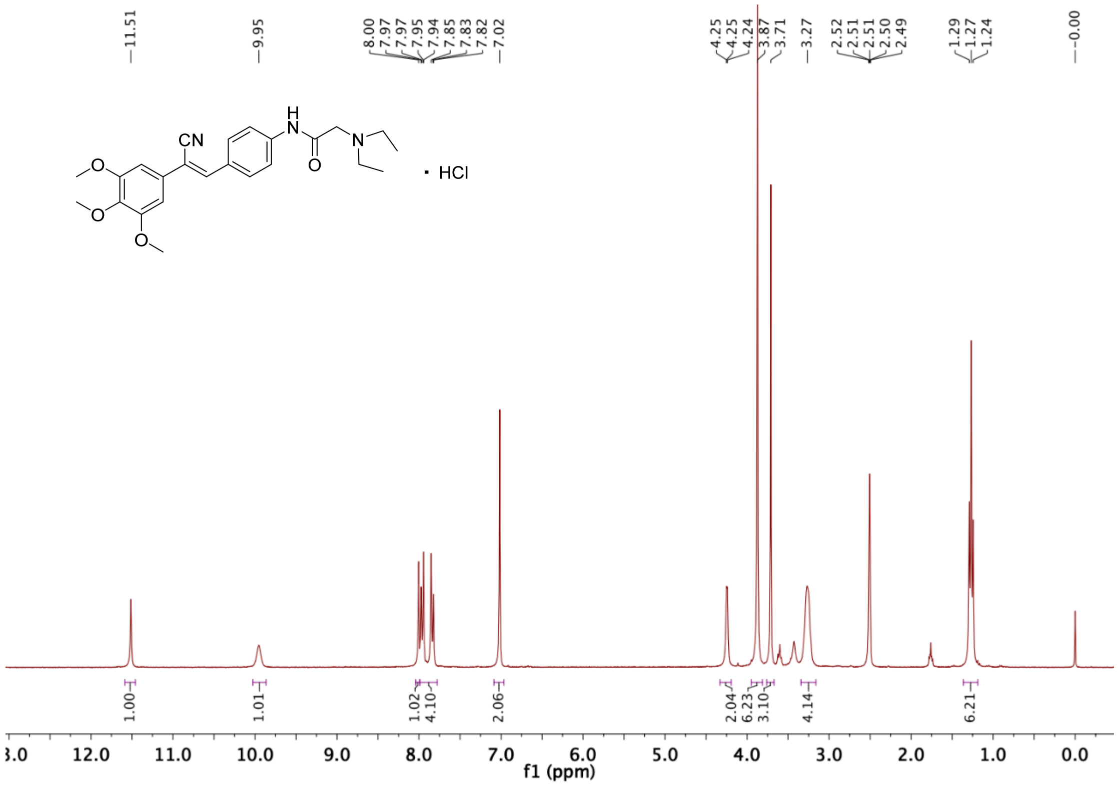

11.51
9.95
8.00
7.97
7.97
7.95
7.94
7.85
7.83
7.82
7.02
4.25
4.25
4.24
3.87
3.71
3.27
2.52
2.51
2.51
2.50
2.49
1.29
1.27
1.24
0.00
CN
H
N
O
N
O
O
O
· HCl
1.00
1.01
1.02
4.10
2.06
2.04
6.23
3.10
4.14
6.21
f1 (ppm)

12a₅.

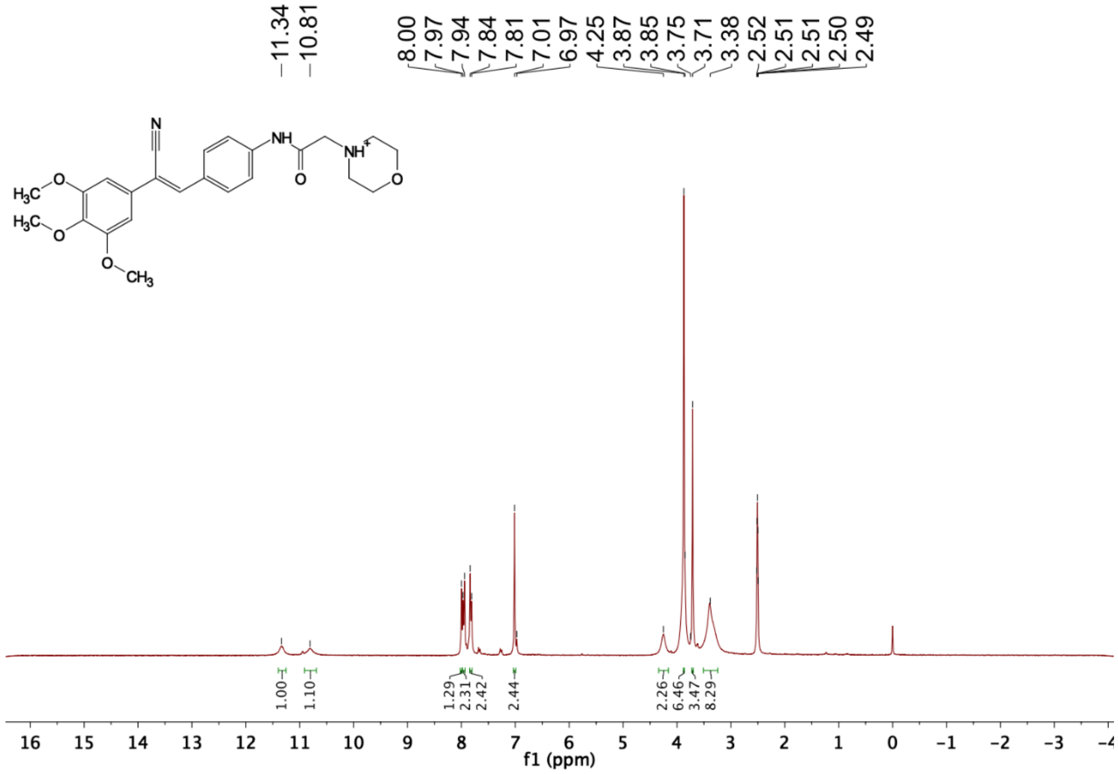

11.34
10.81
8.00
7.97
7.94
7.84
7.81
7.01
6.97
4.25
3.87
3.85
3.75
3.71
3.38
2.52
2.51
2.51
2.50
2.49
N
NH
NH+
O
O
H3C
H3C
CH3
1.00
1.10
1.29
2.31
2.42
2.44
2.26
6.46
3.47
8.29
f1 (ppm)

12a$_6$.

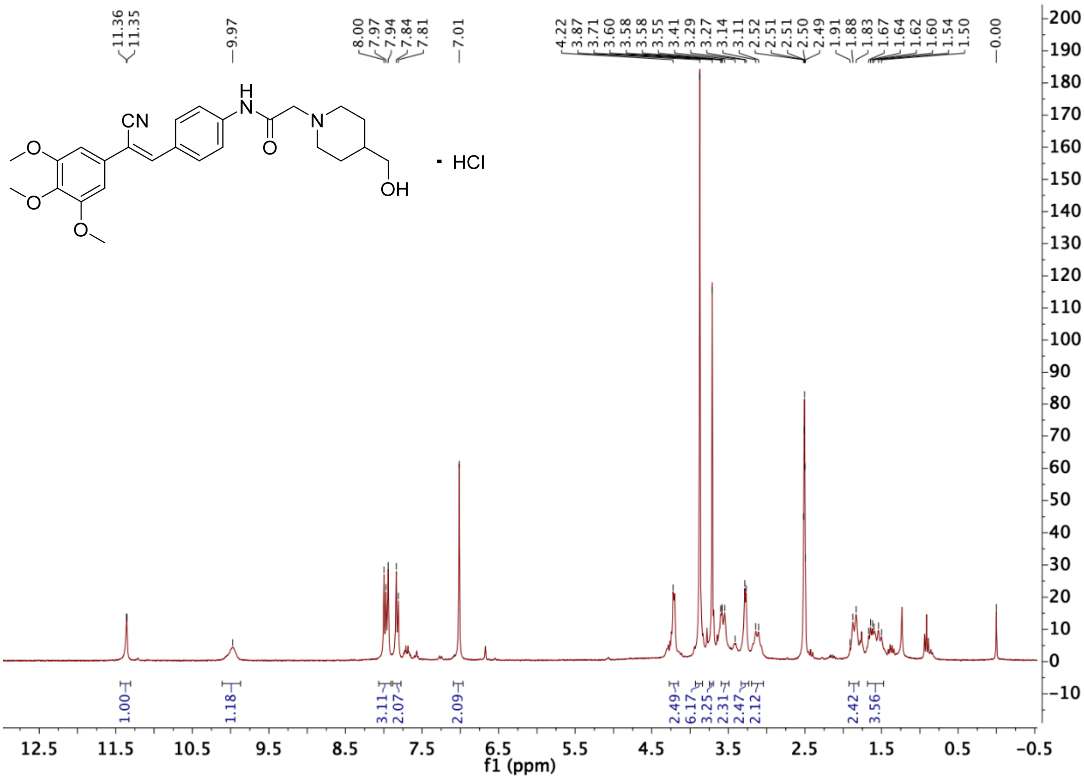

12b$_1$.

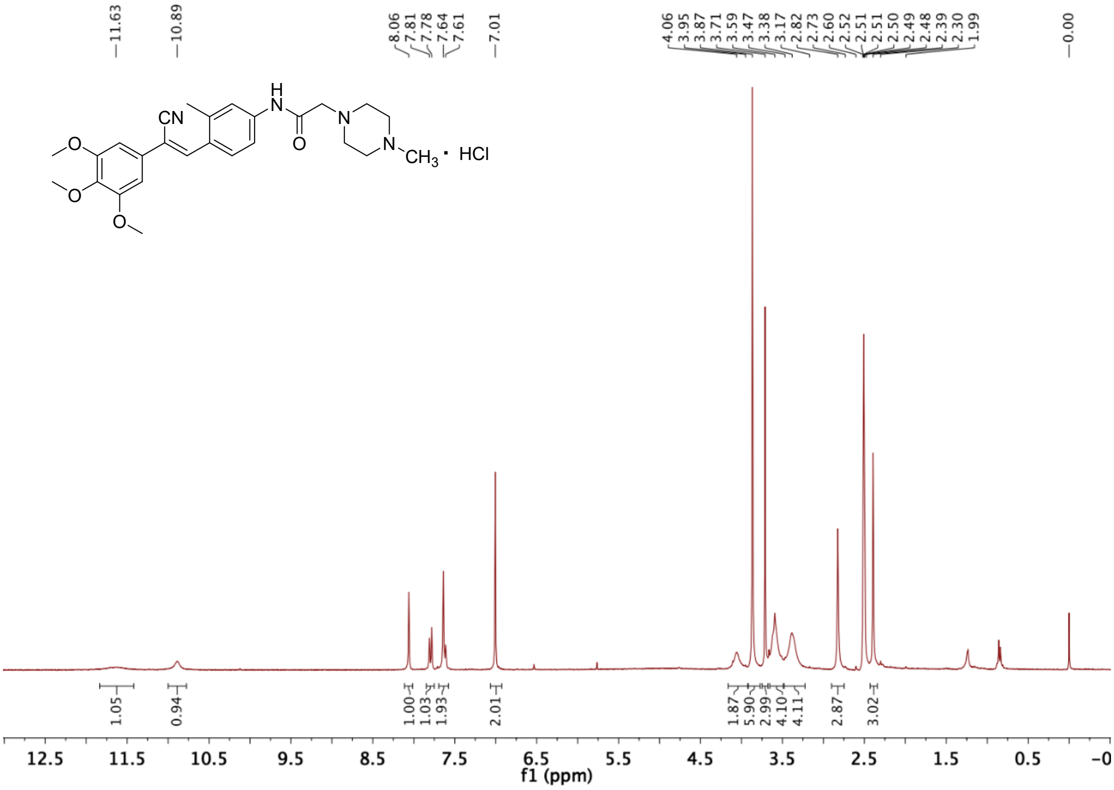

$12b_2$.

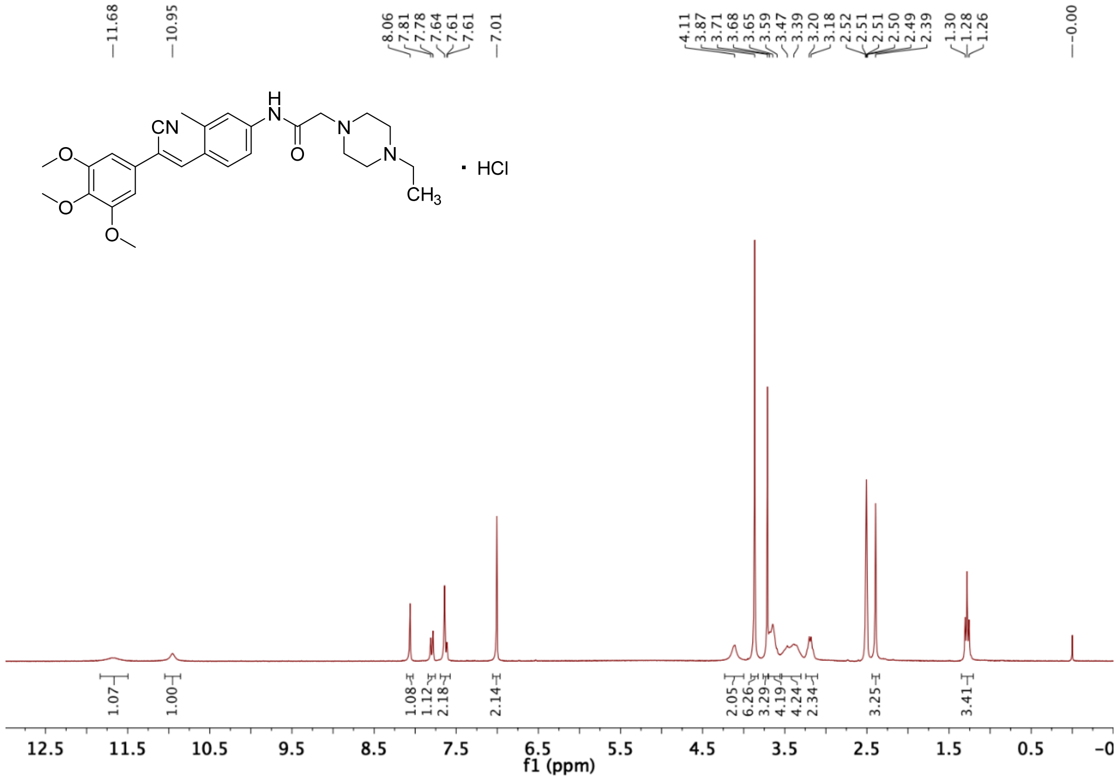

$12c_1$.

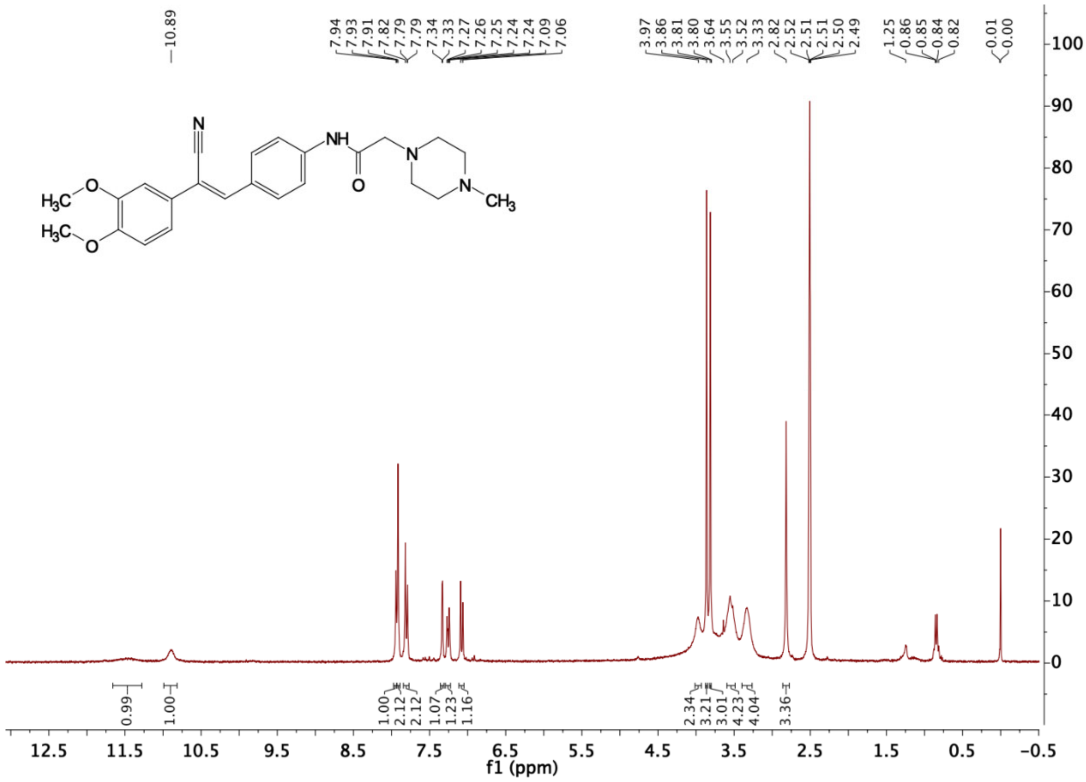

12c₂.

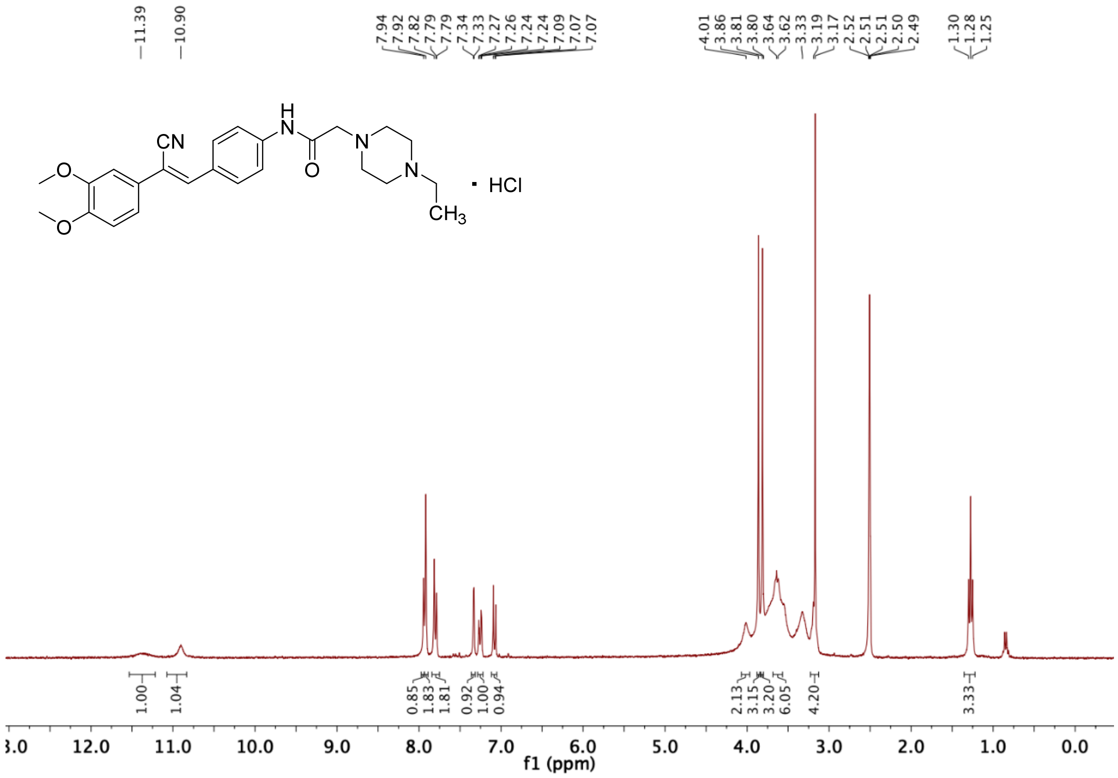

12d₁.

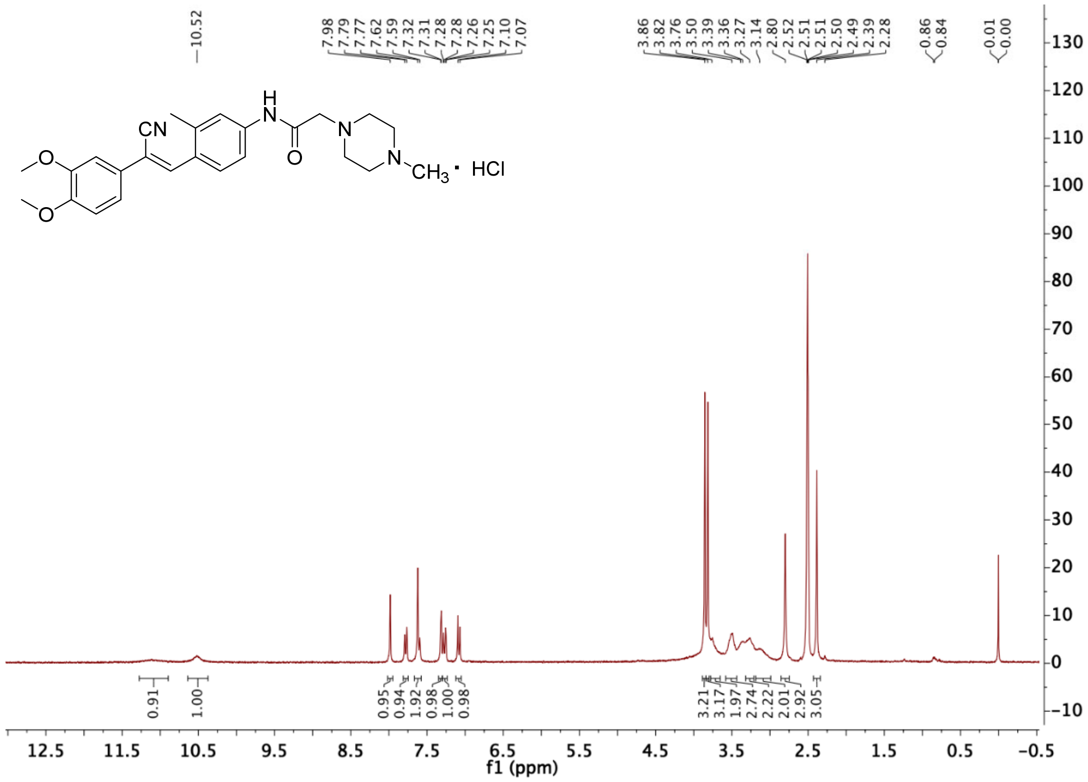

12d$_2$.

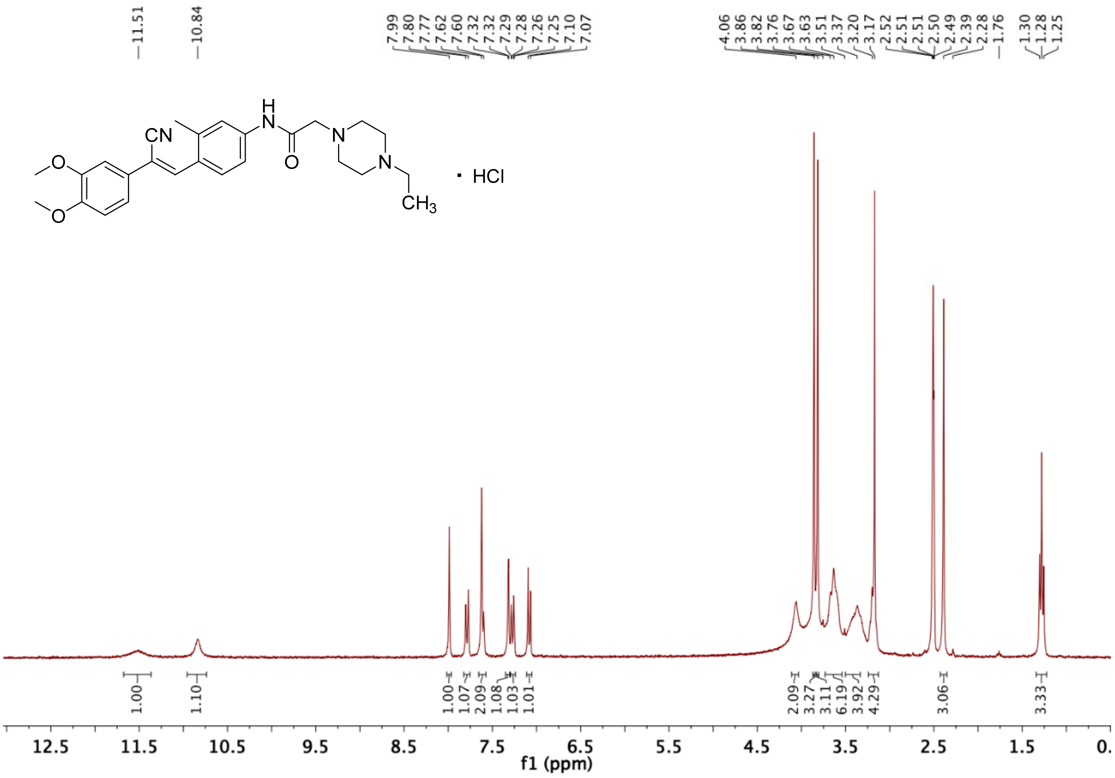
11.51
10.84
7.99 7.80 7.77 7.62 7.60 7.32 7.32 7.29 7.28 7.26 7.25 7.10 7.07
4.06 3.86 3.82 3.76 3.67 3.63 3.51 3.37 3.20 3.17 2.52 2.51 2.51 2.50 2.49 2.39 2.28 1.76 1.30 1.28 1.25
CN
H
N
O
O
O
N
N
CH3
· HCl
1.00
1.10
1.00 1.07 2.09 1.08 1.03 1.01
2.09 3.27 3.11 6.19 3.92 4.29
3.06
3.33
12.5 11.5 10.5 9.5 8.5 7.5 6.5 5.5 4.5 3.5 2.5 1.5 0.
f1 (ppm)

15.

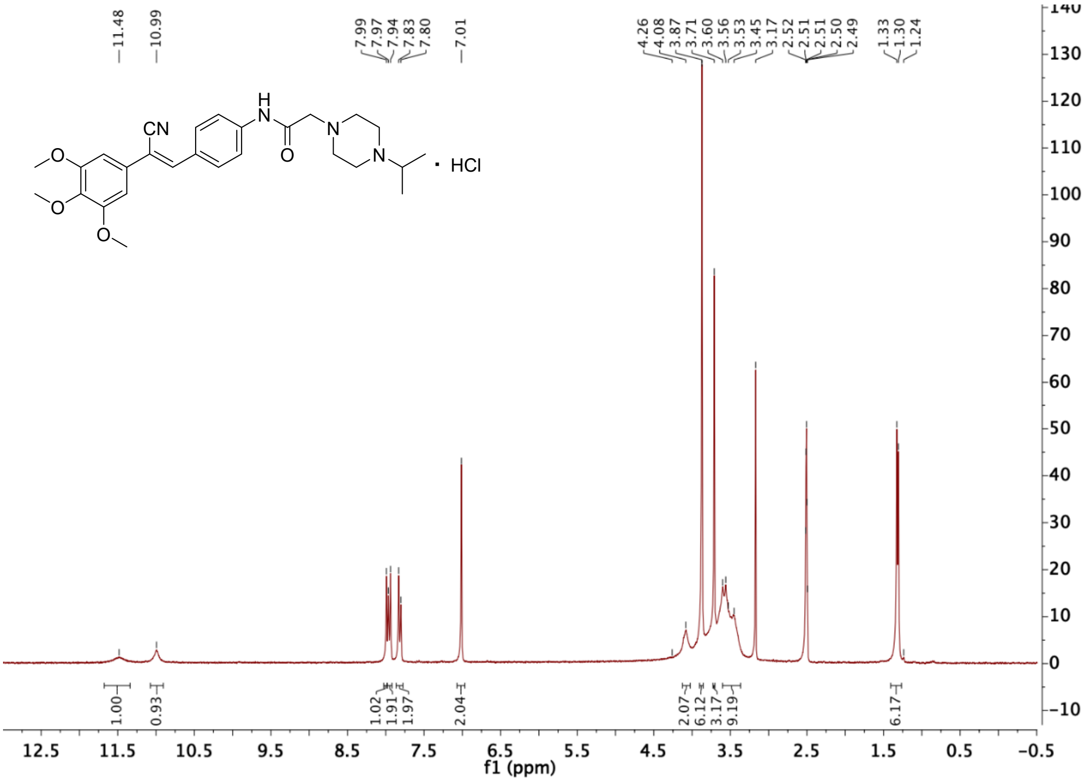
11.48
10.99
7.99 7.97 7.94 7.83 7.80
7.01
4.26 4.08 3.87 3.71 3.60 3.56 3.53 3.45 3.17 2.52 2.51 2.51 2.50 2.49
1.33 1.30 1.24
CN
H
N
O
O
O
O
N
N
· HCl
1.00
0.93
1.02 1.91 1.97
2.04
2.07 6.12 3.17 9.19
6.17
140 130 120 110 100 90 80 70 60 50 40 30 20 10 0 −10
12.5 11.5 10.5 9.5 8.5 7.5 6.5 5.5 4.5 3.5 2.5 1.5 0.5 −0.5
f1 (ppm)

16.

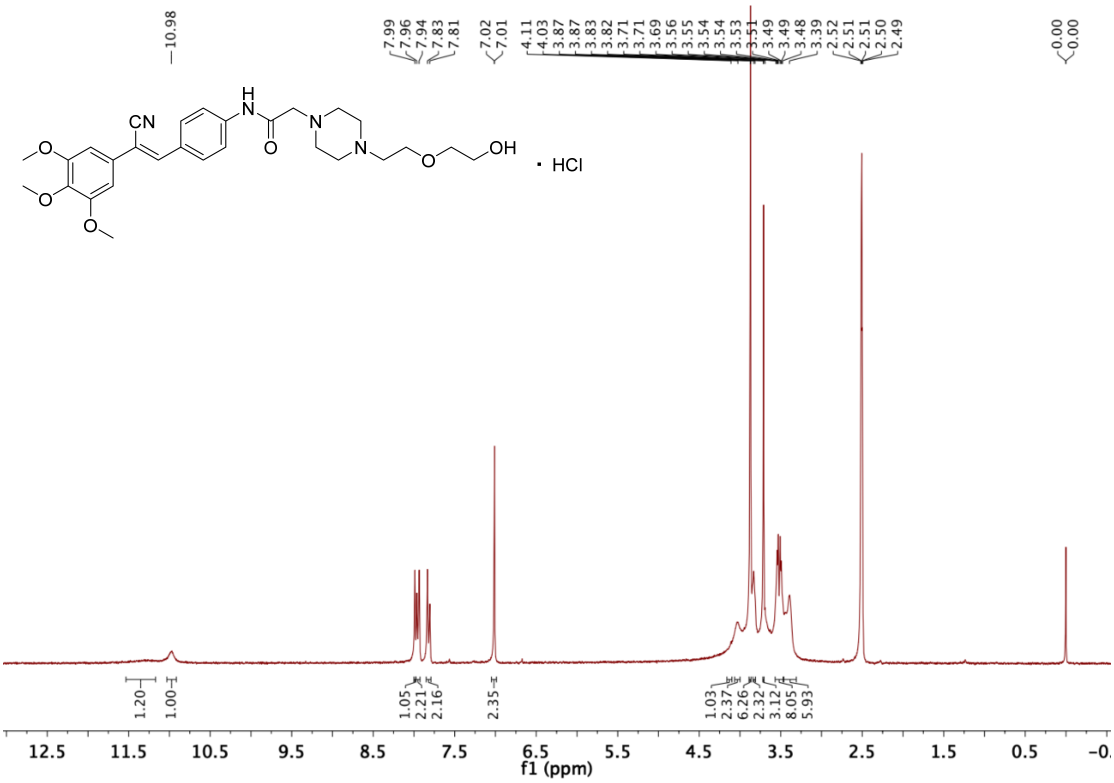

17.

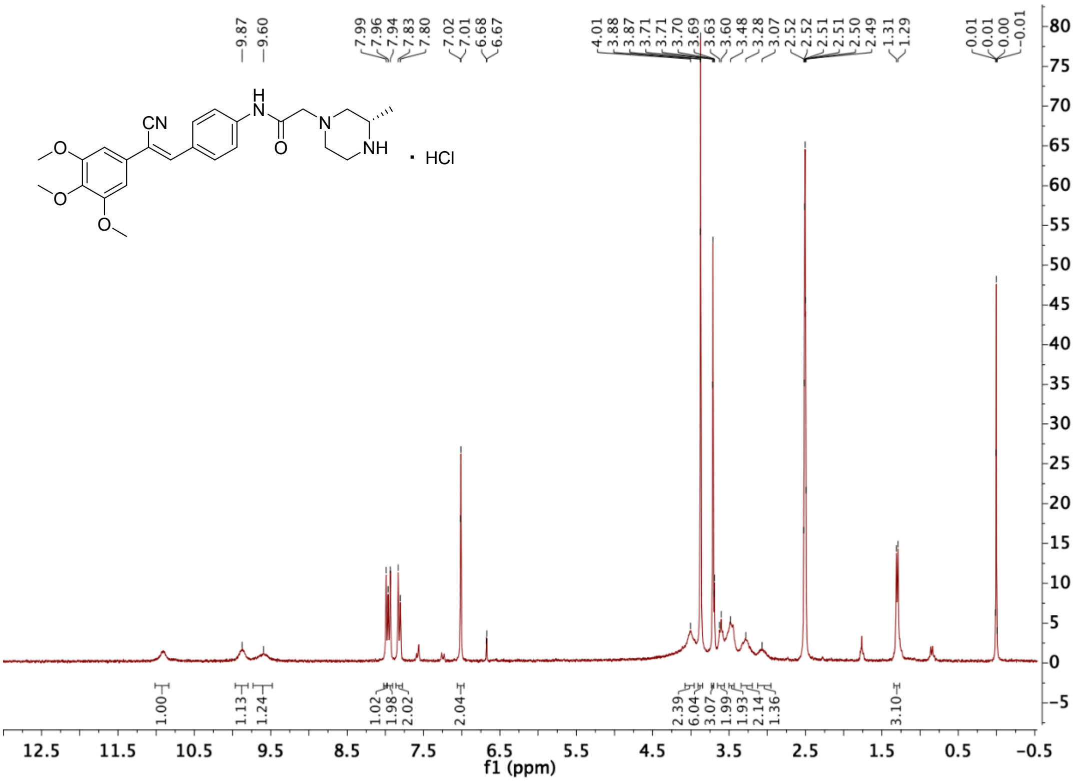

18.

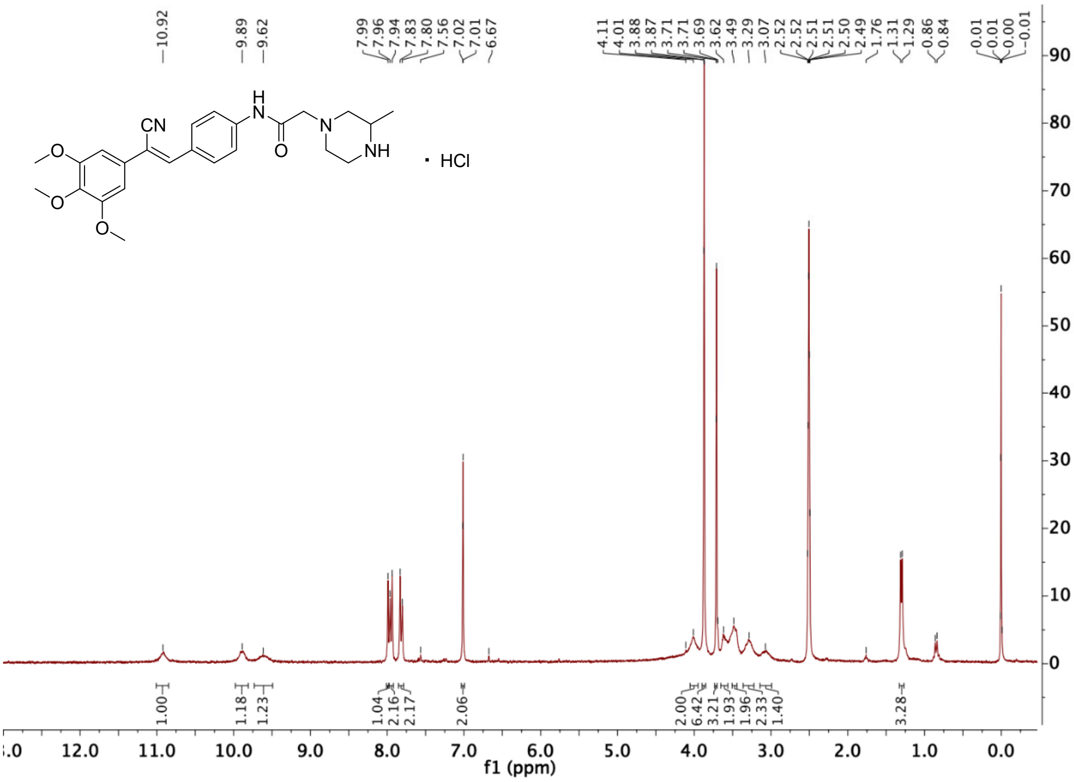

19.

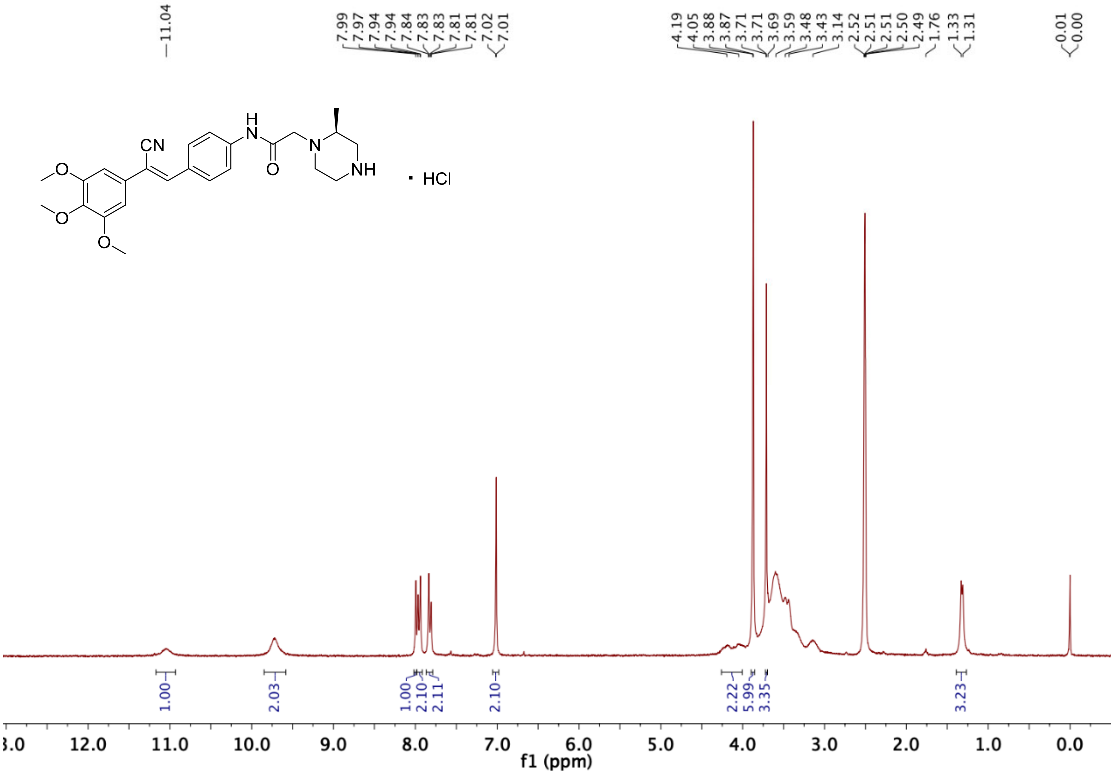

20.

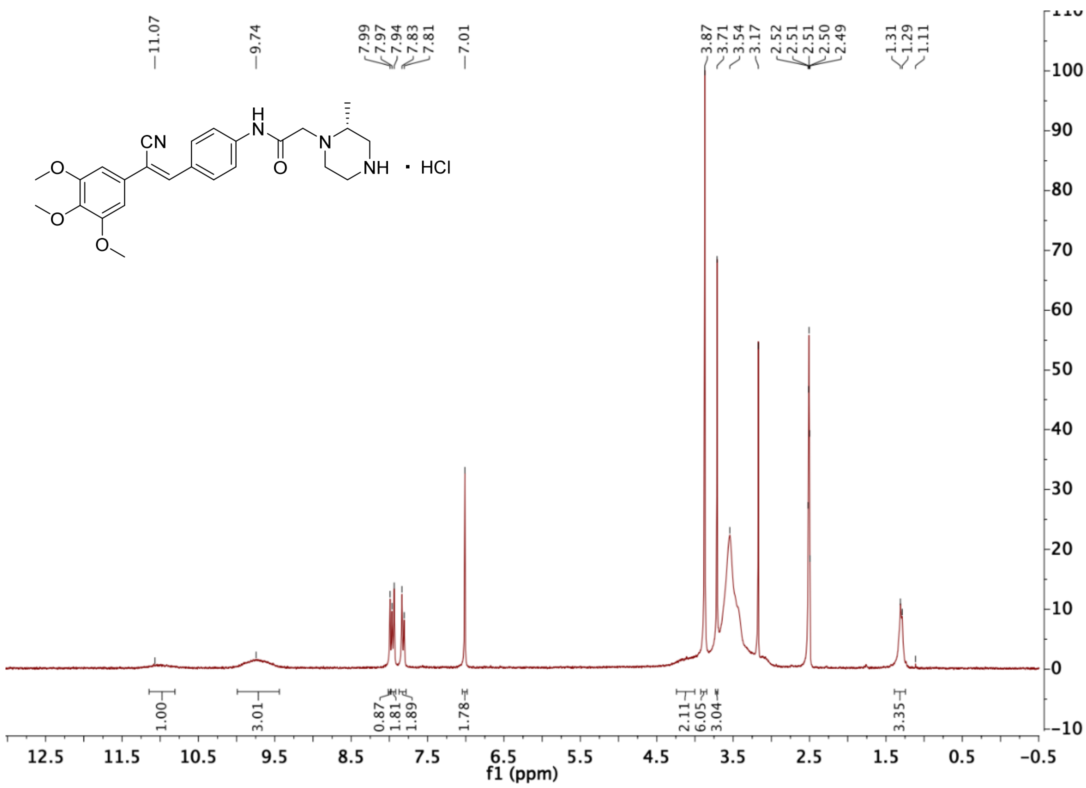

4.2. ^{13}C NMR

9a.

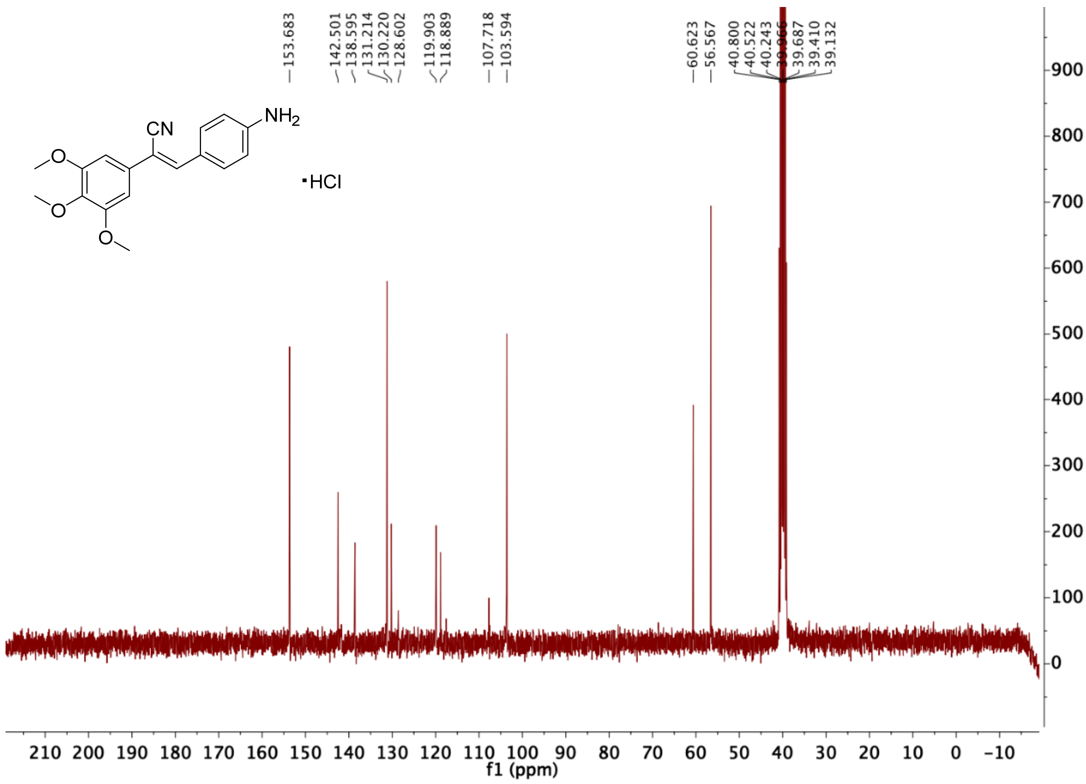

9b.

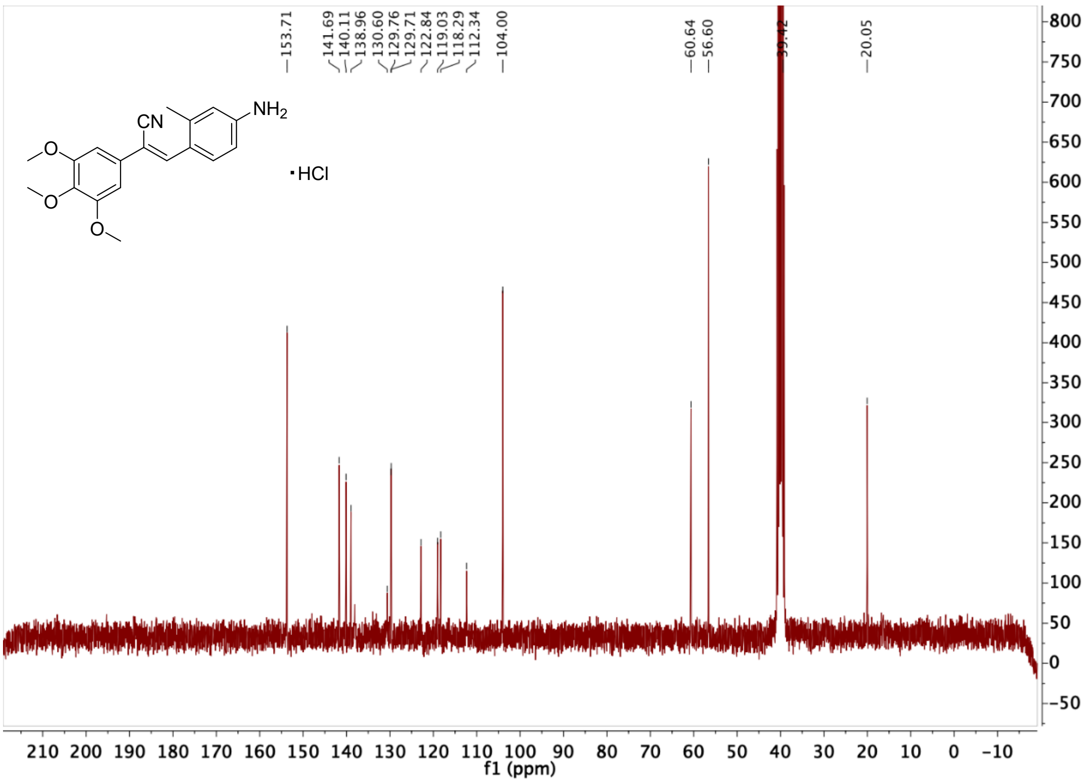

153.71
141.69
140.11
138.96
130.60
129.76
129.71
122.84
119.03
118.29
112.34
104.00
60.64
56.60
20.05
CN
NH2
O
O
O
•HCl
f1 (ppm)

9c.

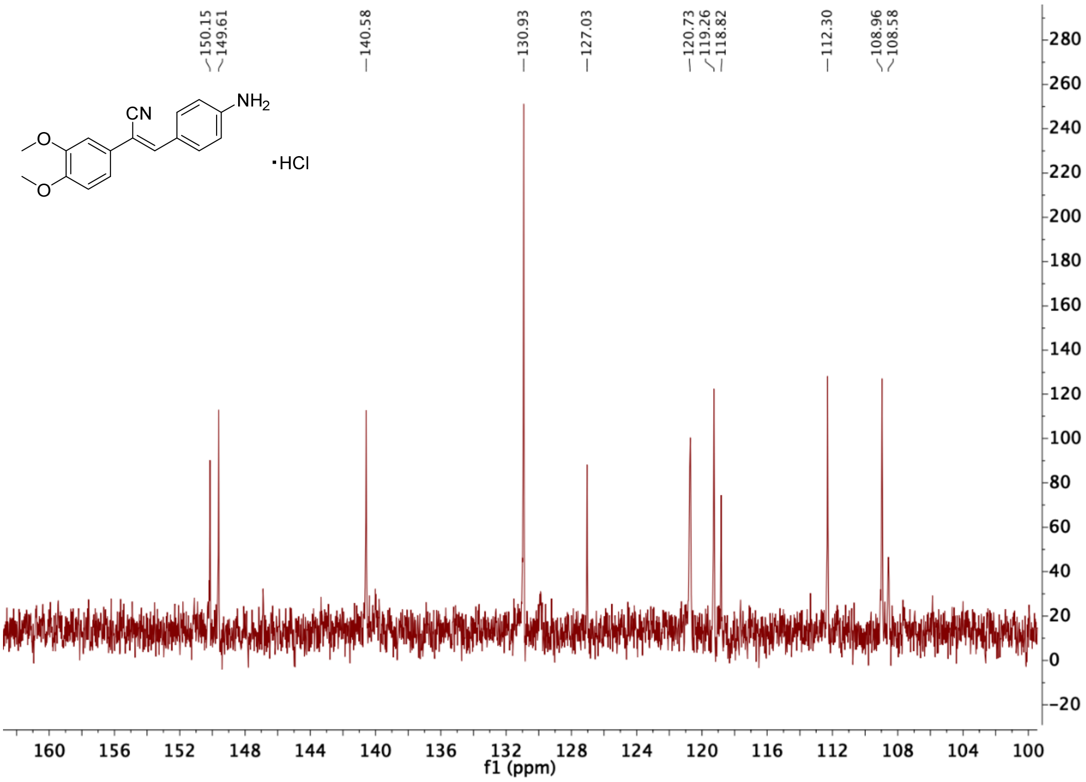

150.15
149.61
140.58
130.93
127.03
120.73
119.26
118.82
112.30
108.96
108.58
CN
NH2
O
O
•HCl
f1 (ppm)

9d.

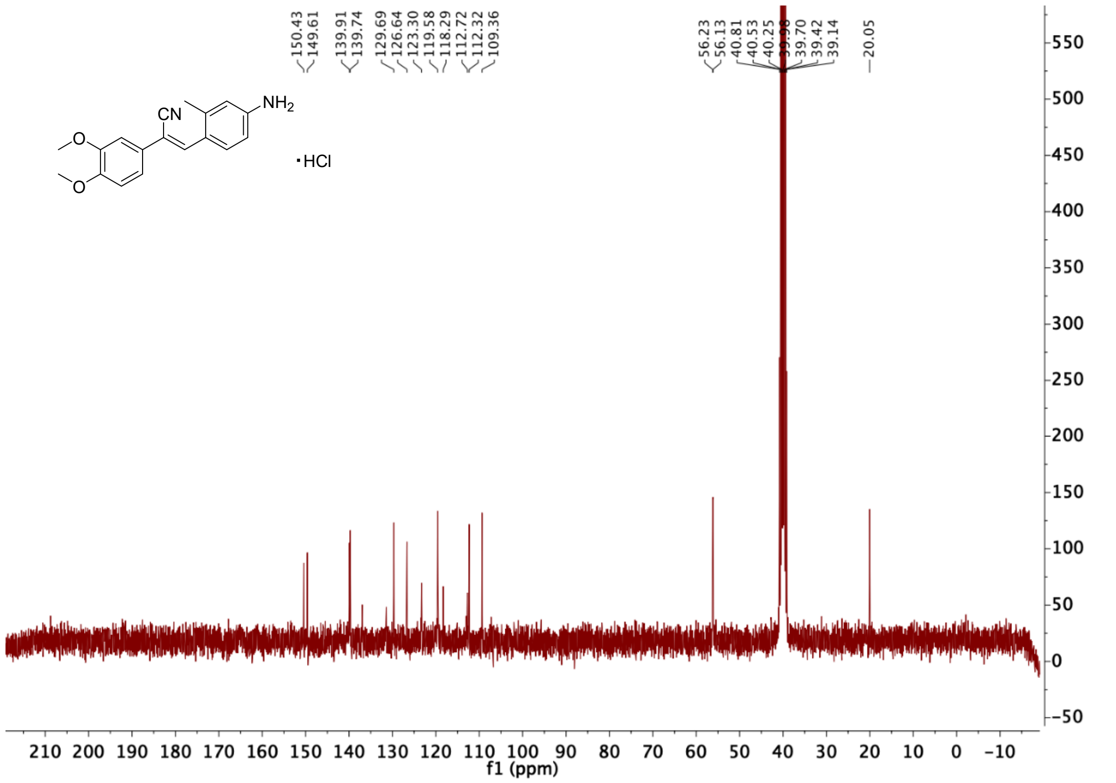
150.43
149.61
139.91
139.74
129.69
126.64
123.30
119.58
118.29
112.72
112.32
109.36
56.23
56.13
40.81
40.53
40.25
[illegible]
39.70
39.42
39.14
20.05
CN
NH2
•HCl
f1 (ppm)

12a$_1$.

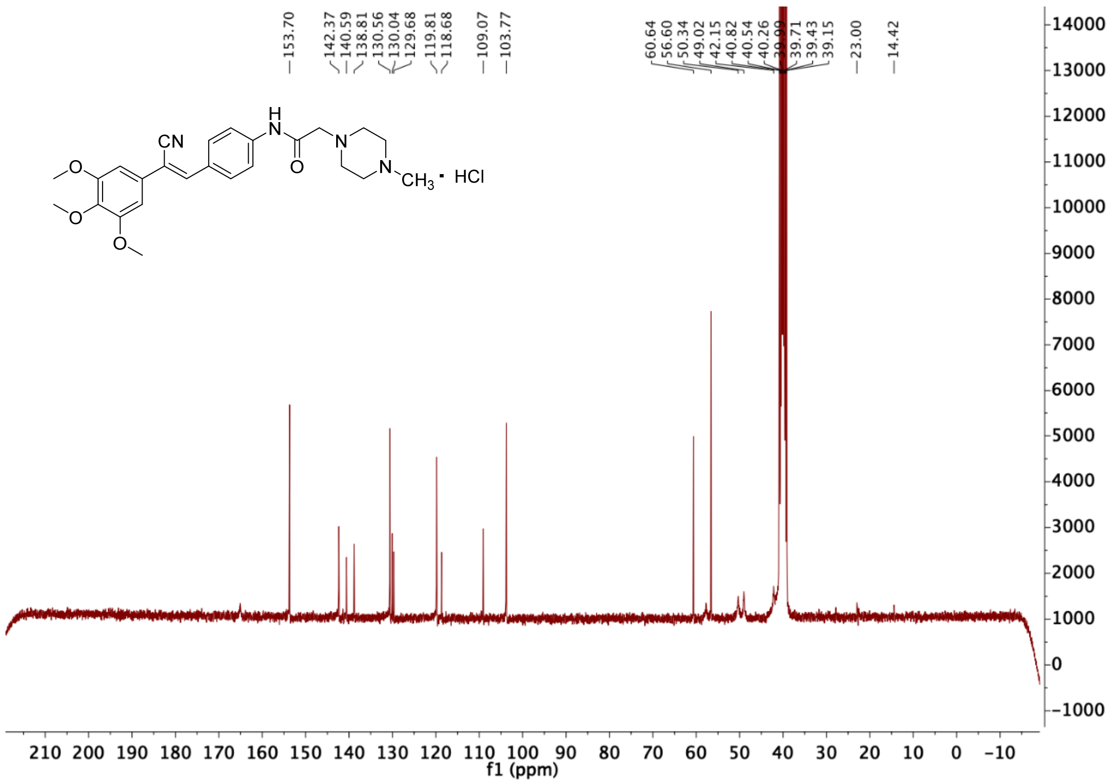
153.70
142.37
140.59
138.81
130.56
130.04
129.68
119.81
118.68
109.07
103.77
60.64
56.60
50.34
49.02
42.15
40.82
40.54
40.26
[illegible]
39.71
39.43
39.15
23.00
14.42
CN
H
N
O
N
N
CH3
HCl
f1 (ppm)

12a2.

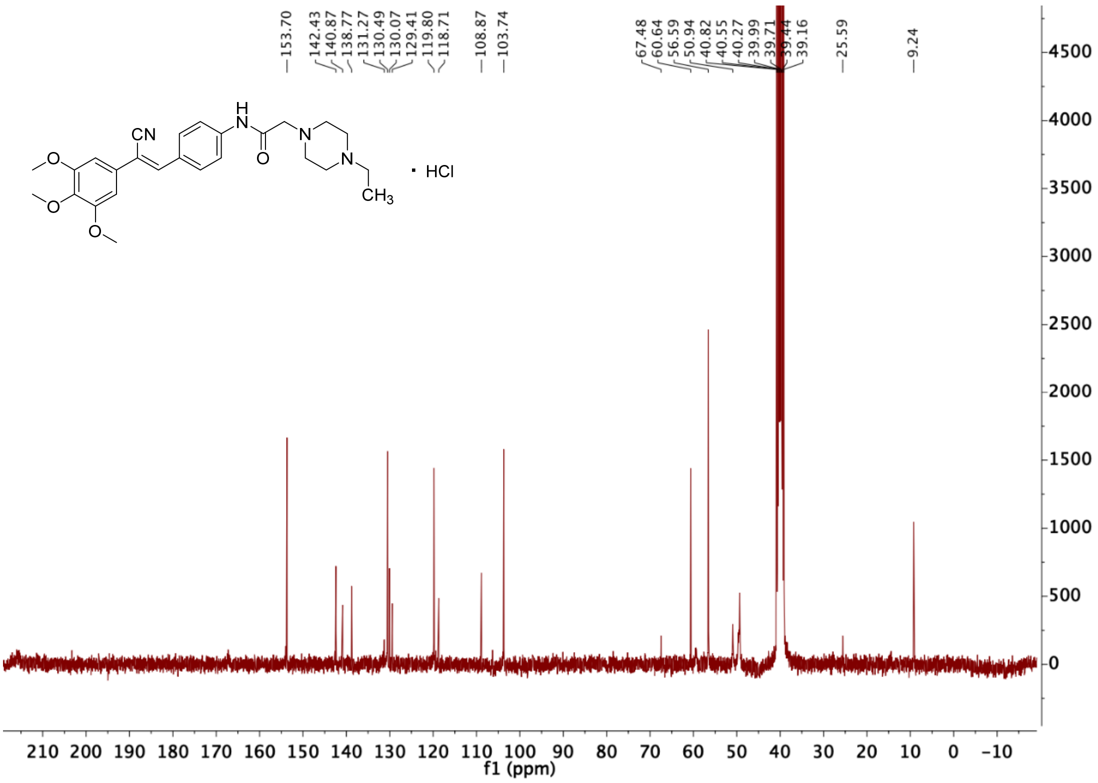

153.70
142.43
140.87
138.77
131.27
130.49
130.07
129.41
119.80
118.71
108.87
103.74
67.48
60.64
56.59
50.94
40.82
40.55
40.27
39.99
39.71
39.44
39.16
25.59
9.24
CN
H
N
O
N
N
CH3
O
O
O
· HCl
f1 (ppm)

12a3.

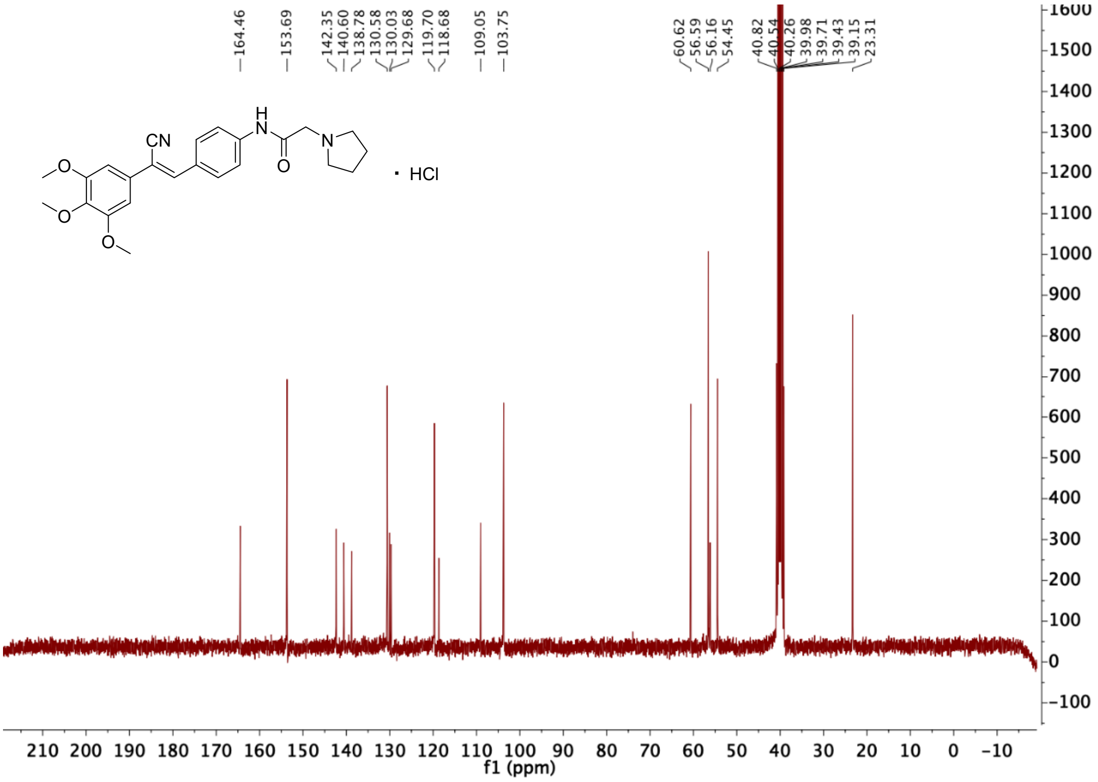

164.46
153.69
142.35
140.60
138.78
130.58
130.03
129.68
119.70
118.68
109.05
103.75
60.62
56.59
56.16
54.45
40.82
40.54
40.26
39.98
39.71
39.43
39.15
23.31
CN
H
N
O
N
O
O
O
· HCl
f1 (ppm)

12a$_4$.

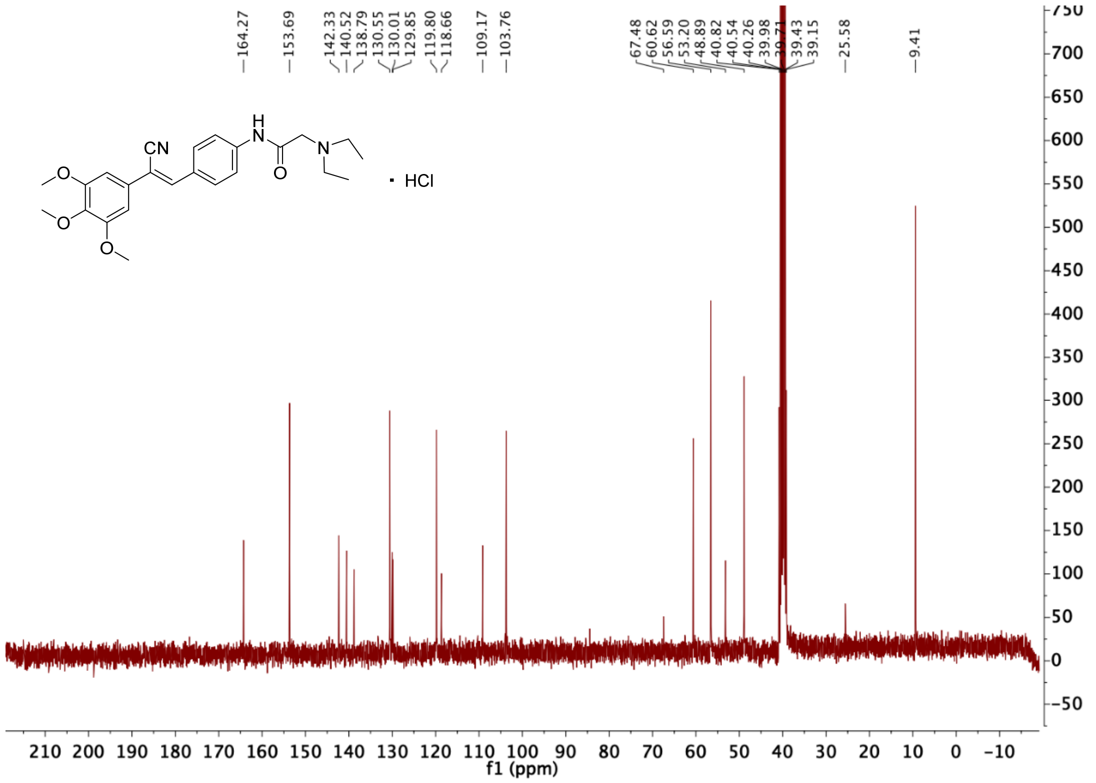

12a$_5$.

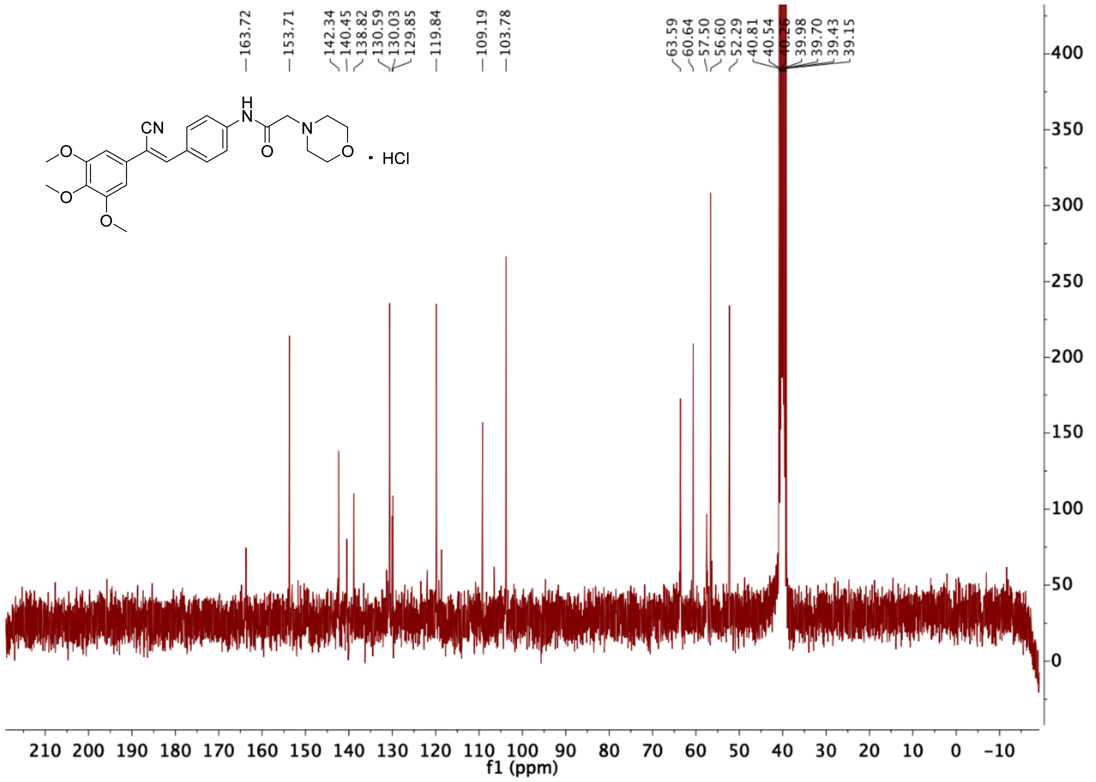

12a₆.

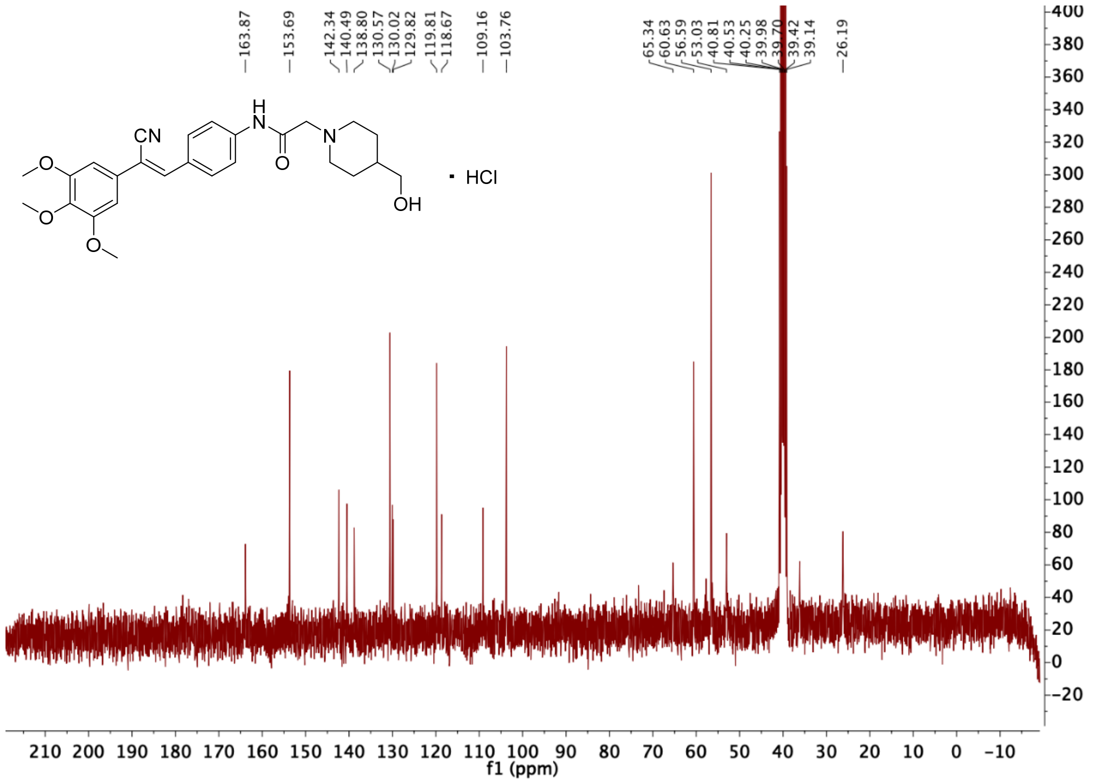

12b₁.

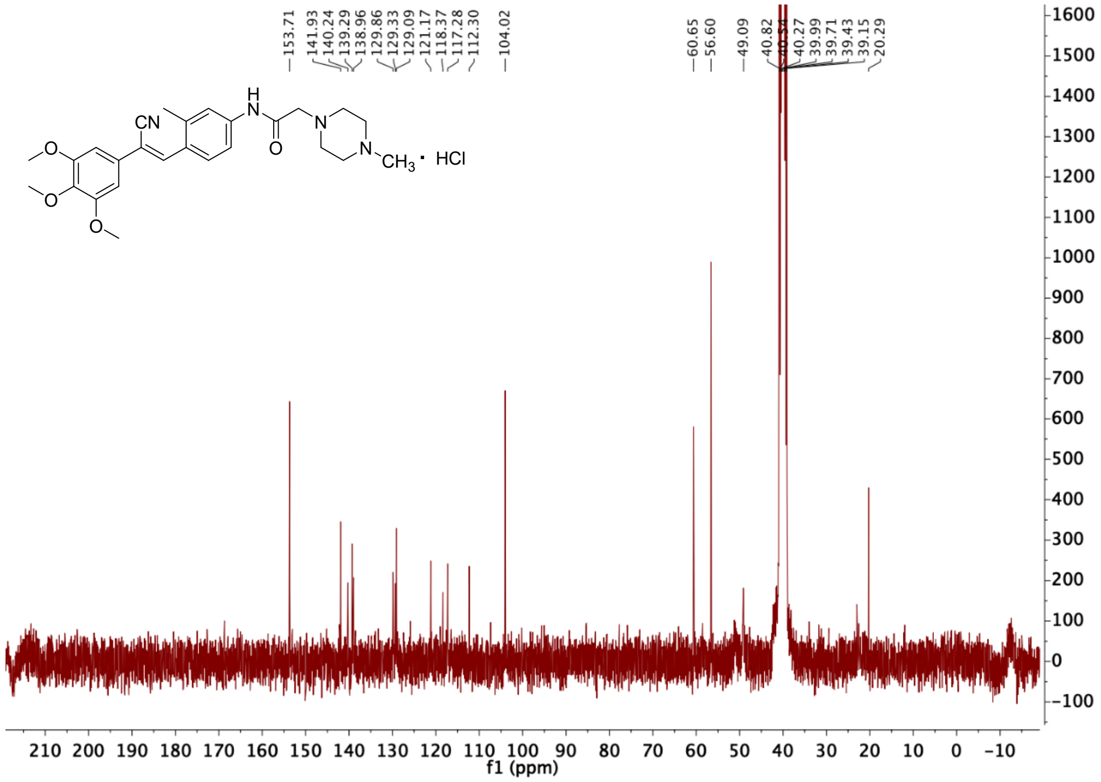

12b₂.

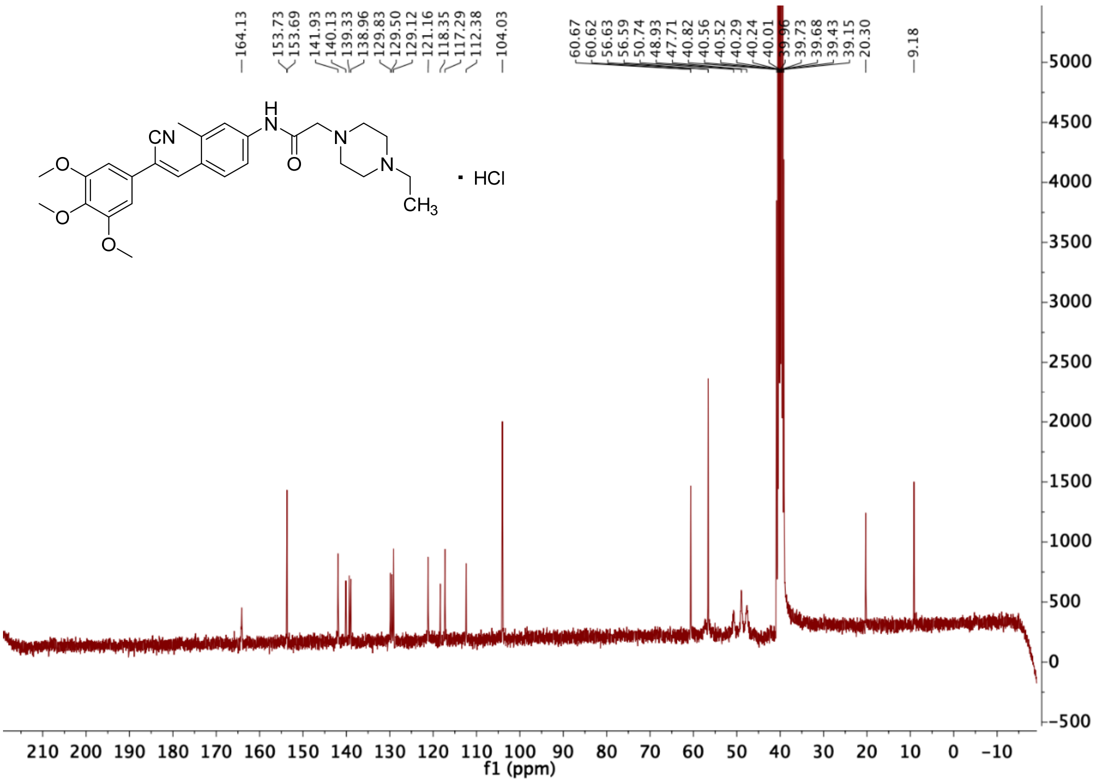

12c₁.

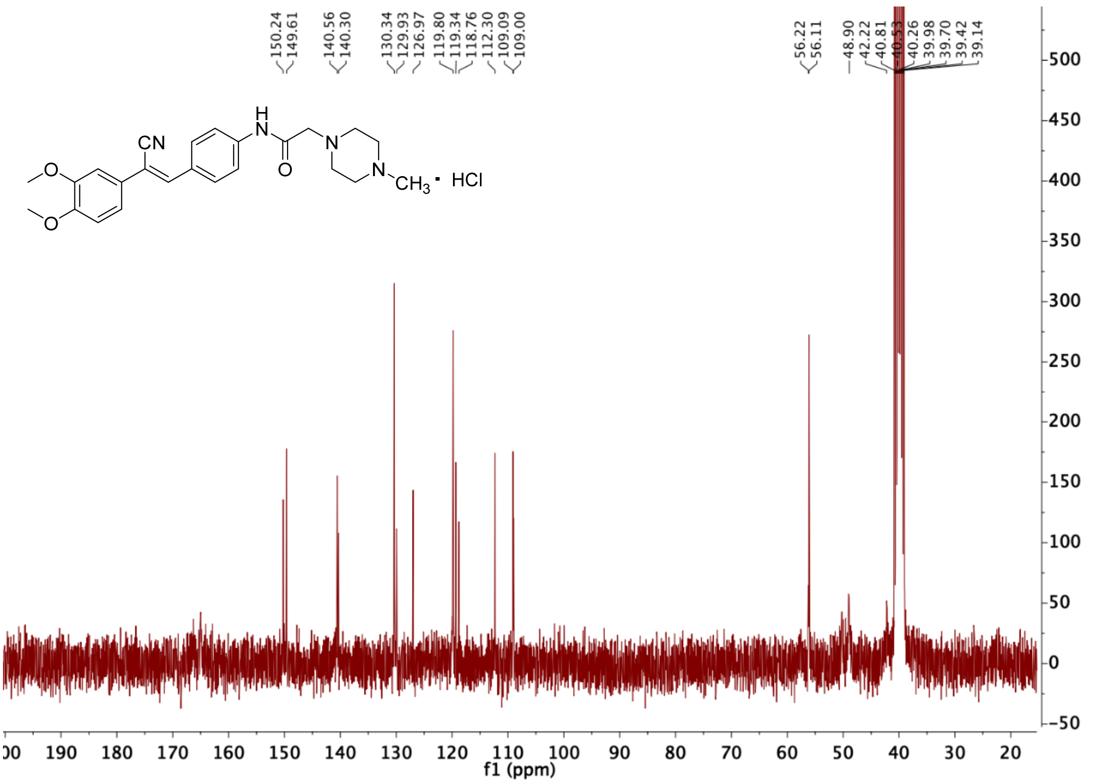

12c₂.

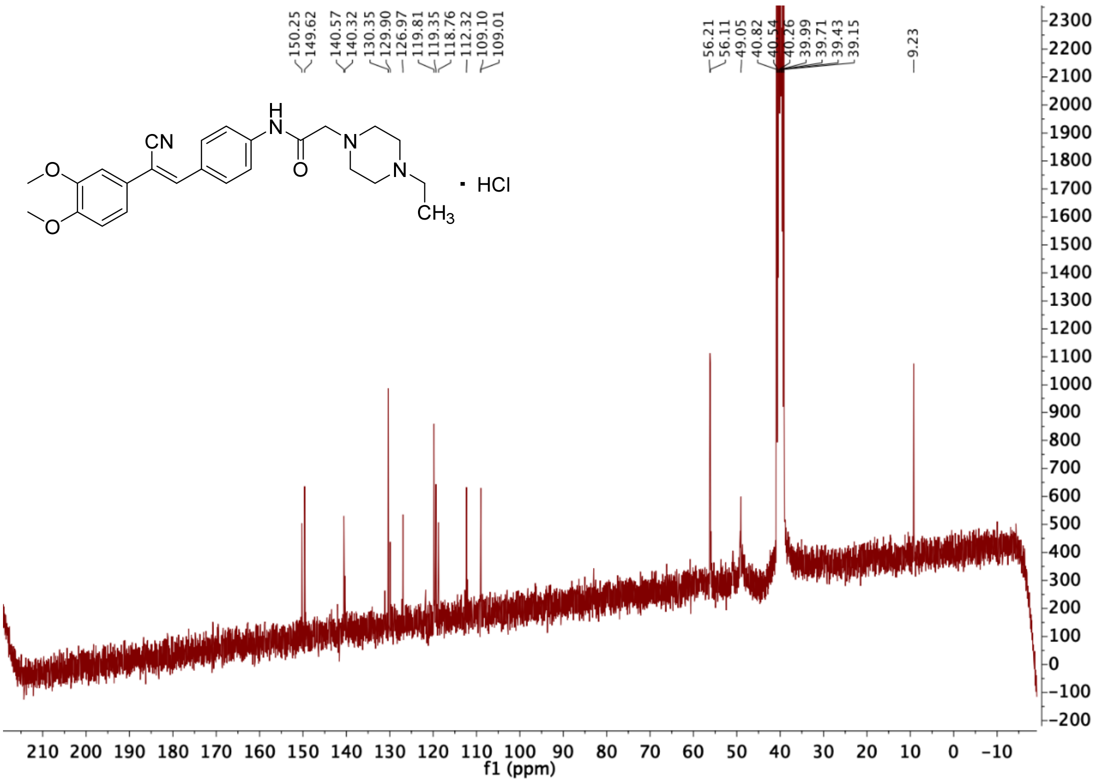

12d₁.

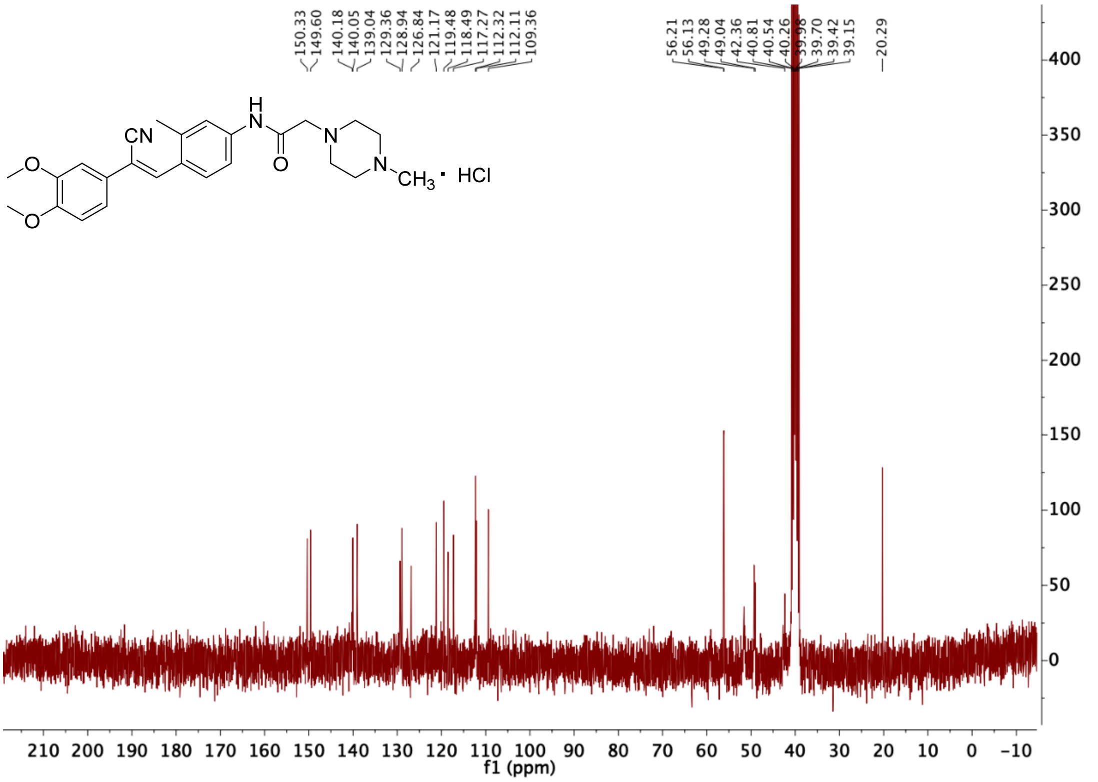

12d₂.

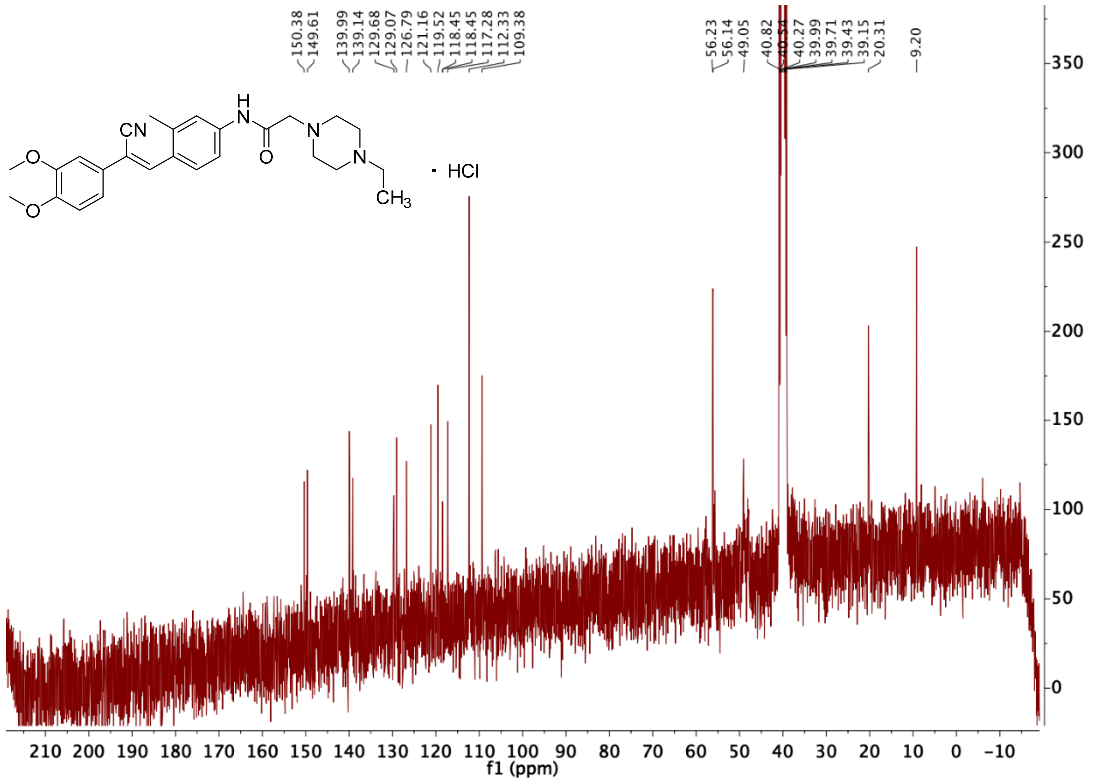

15.

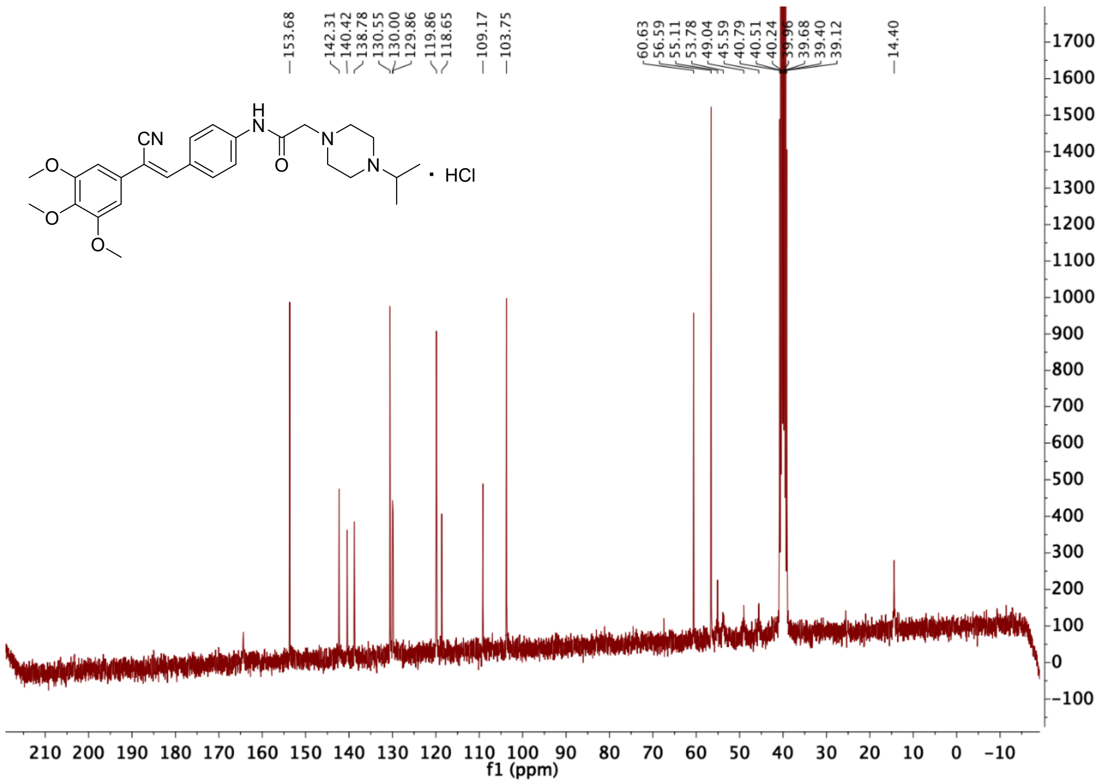

16.

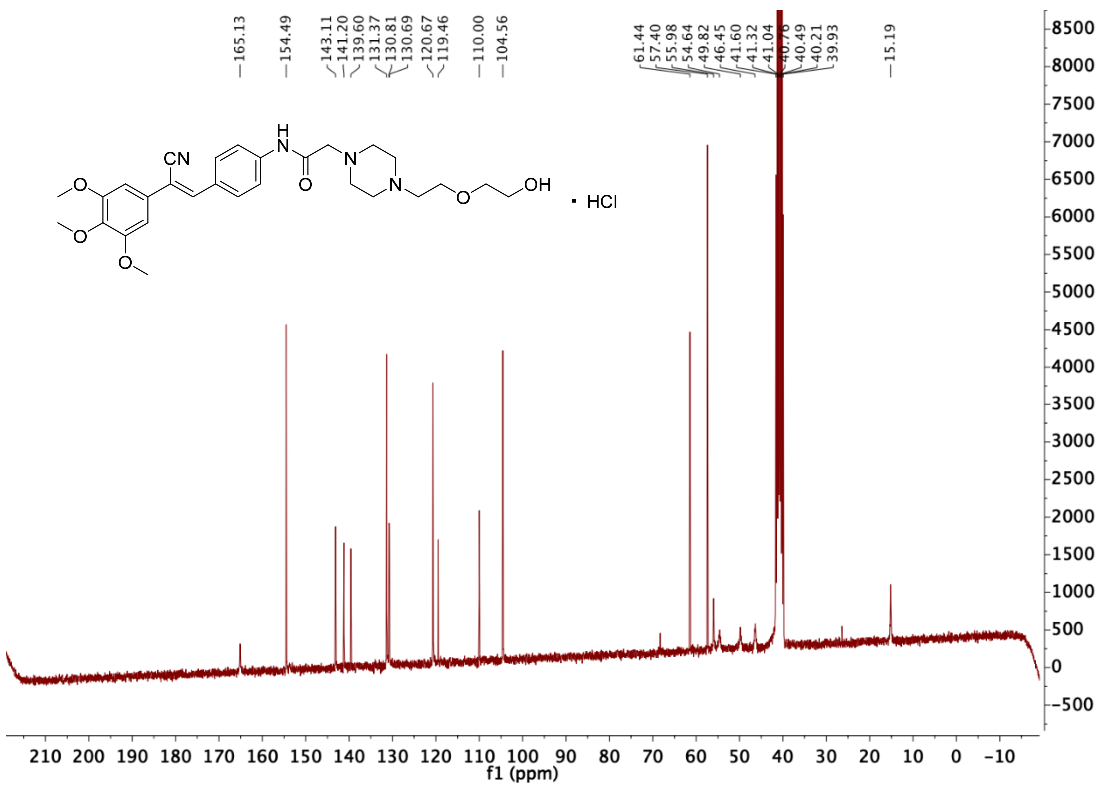

17.

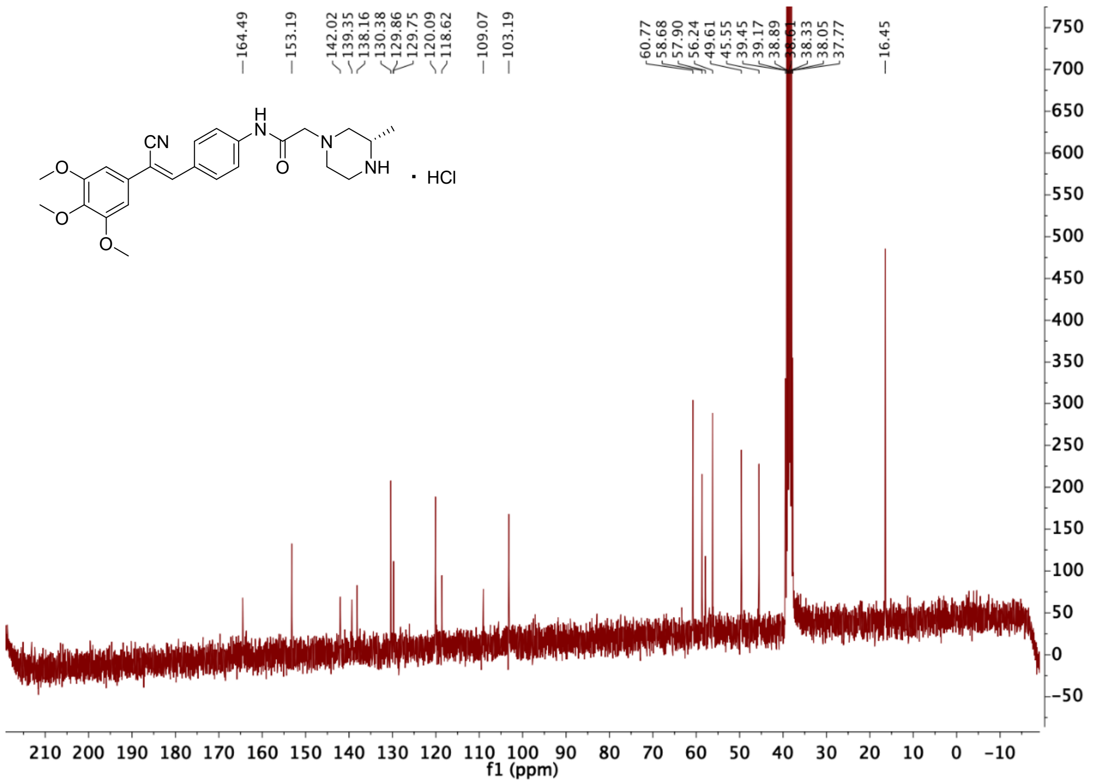

18.

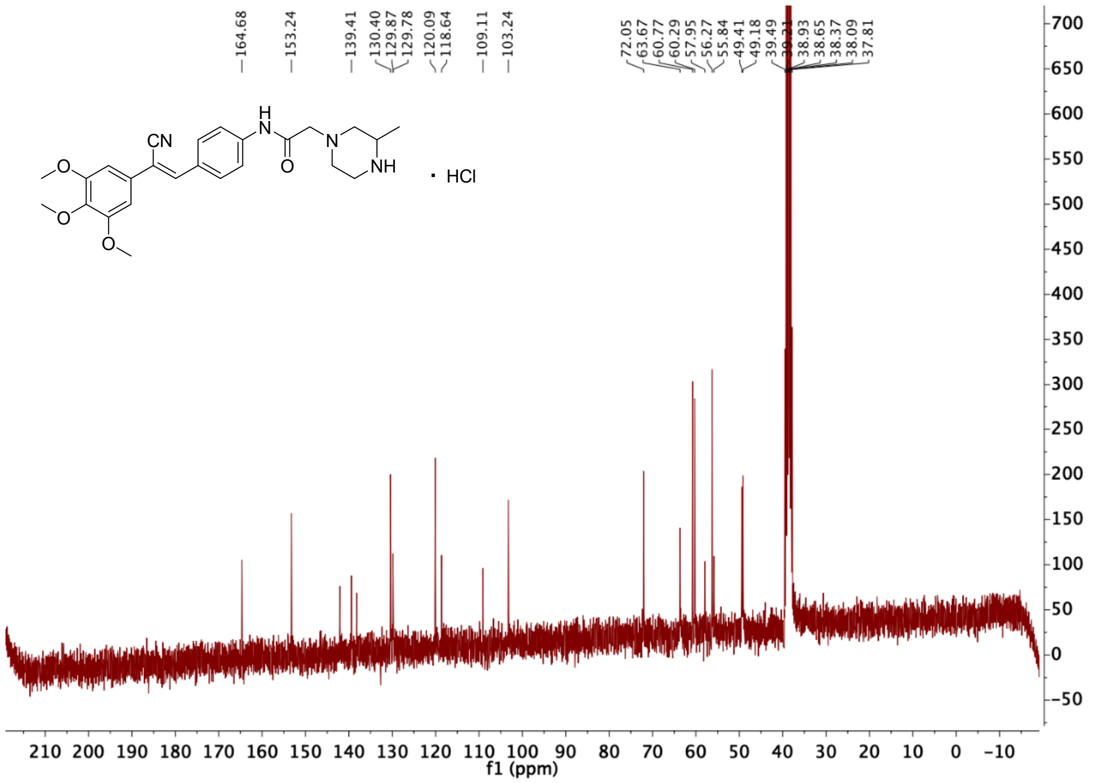

19.

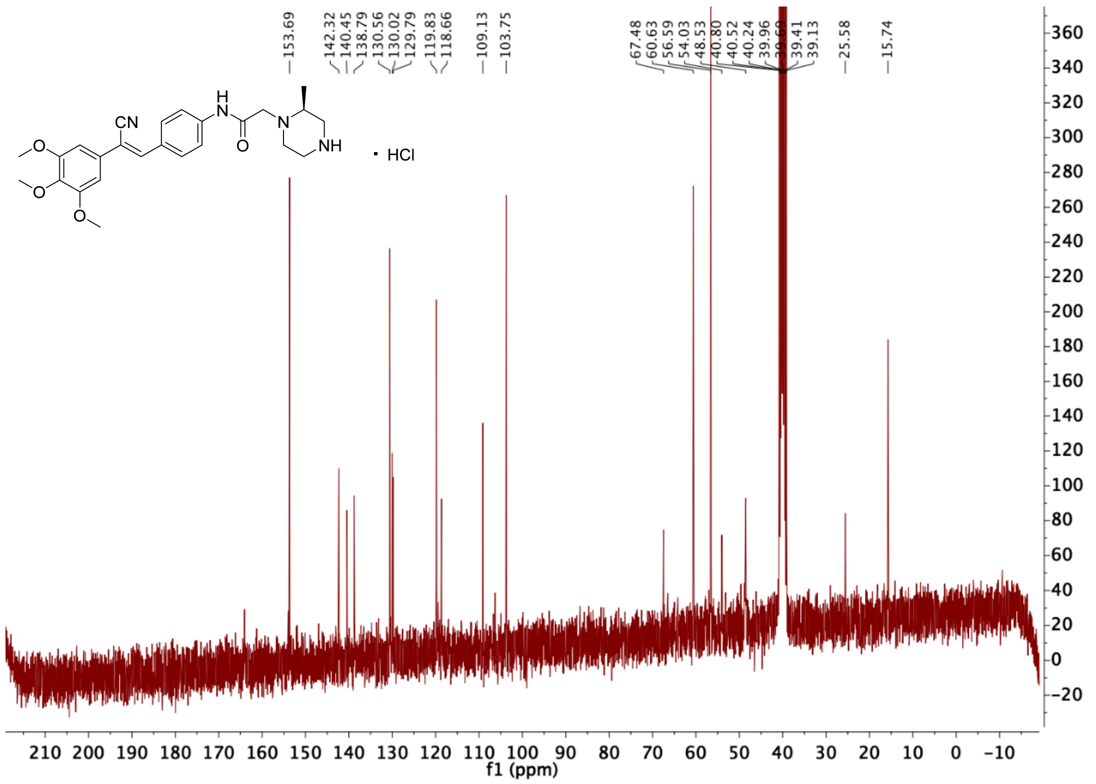

20.

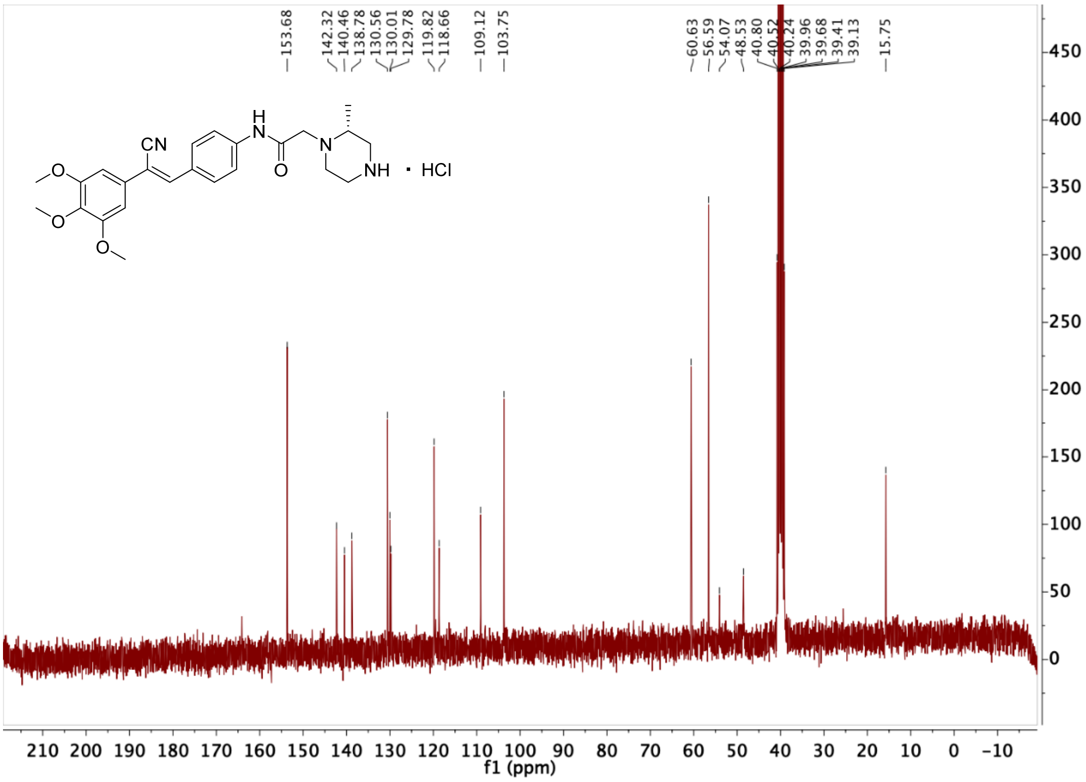

4.3. HRMS

9a. HRMS (ESI) m/z calcd for $C_{18}H_{19}N_2O_3^+$ $(M+H)^+$ 311.13902, found 311.13889.

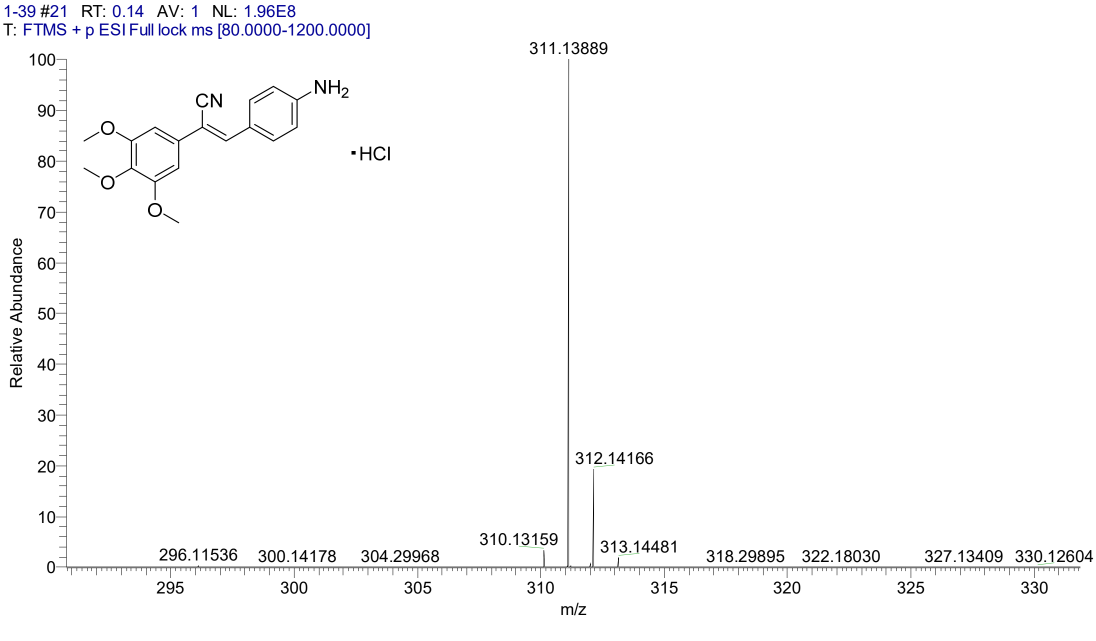

9b. HRMS (ESI) m/z calcd for $C_{19}H_{21}N_2O_3^+$ $(M+H)^+$ 325.15467, found 325.15460.

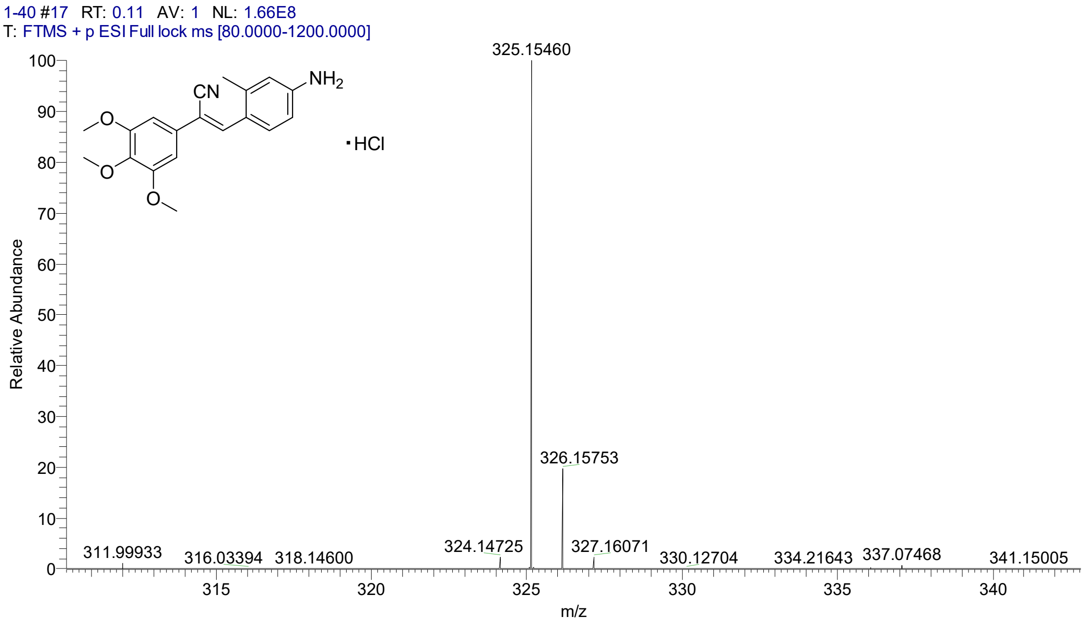

9c. HRMS (ESI) m/z calcd for $C_{17}H_{17}N_2O_2^+$ $(M+H)^+$ 281.12845, found 281.12851.

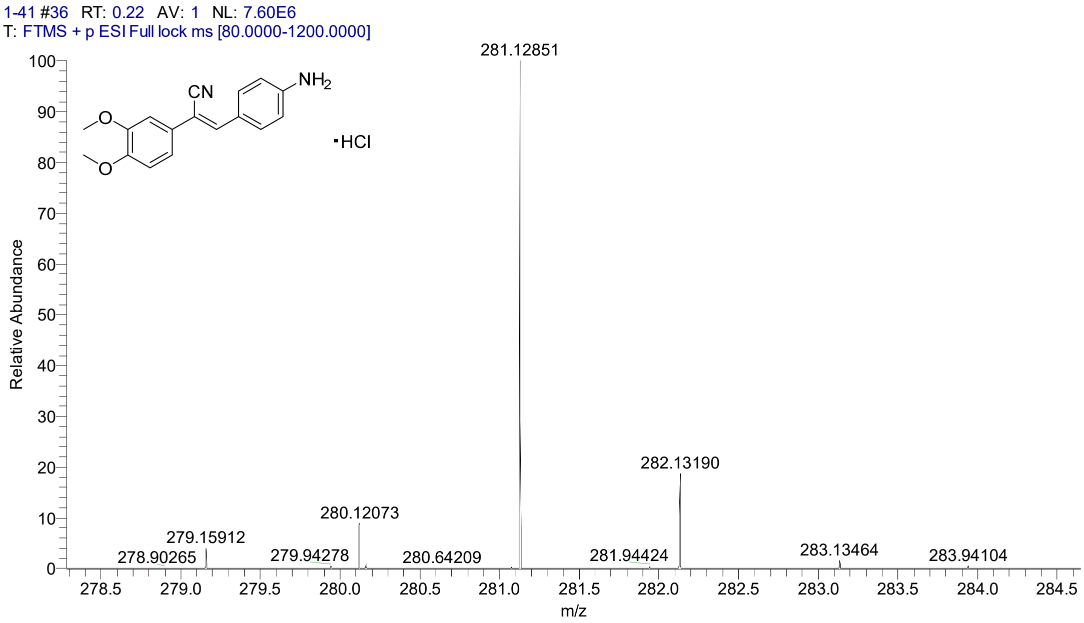

9d. HRMS (ESI) m/z calcd for $C_{18}H_{19}N_2O_2^+$ $(M+H)^+$ 295.14410, found 295.14389.

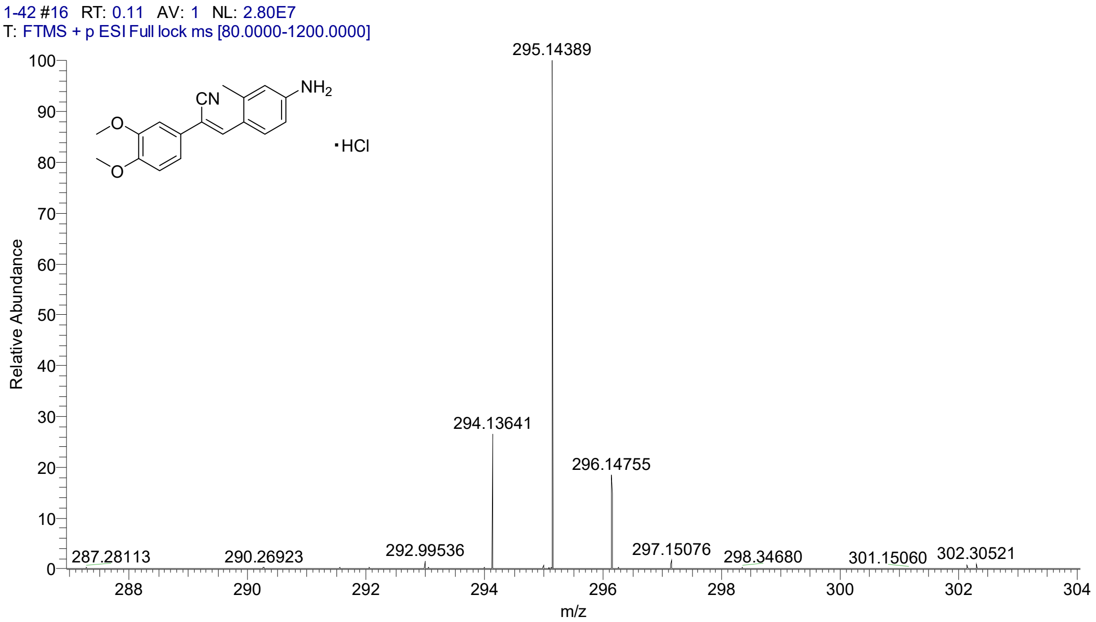

12a₁. HRMS (ESI) m/z calcd for $C_{25}H_{31}N_4O_4^+$ $(M+H)^+$ 451.23398, found 451.23396.

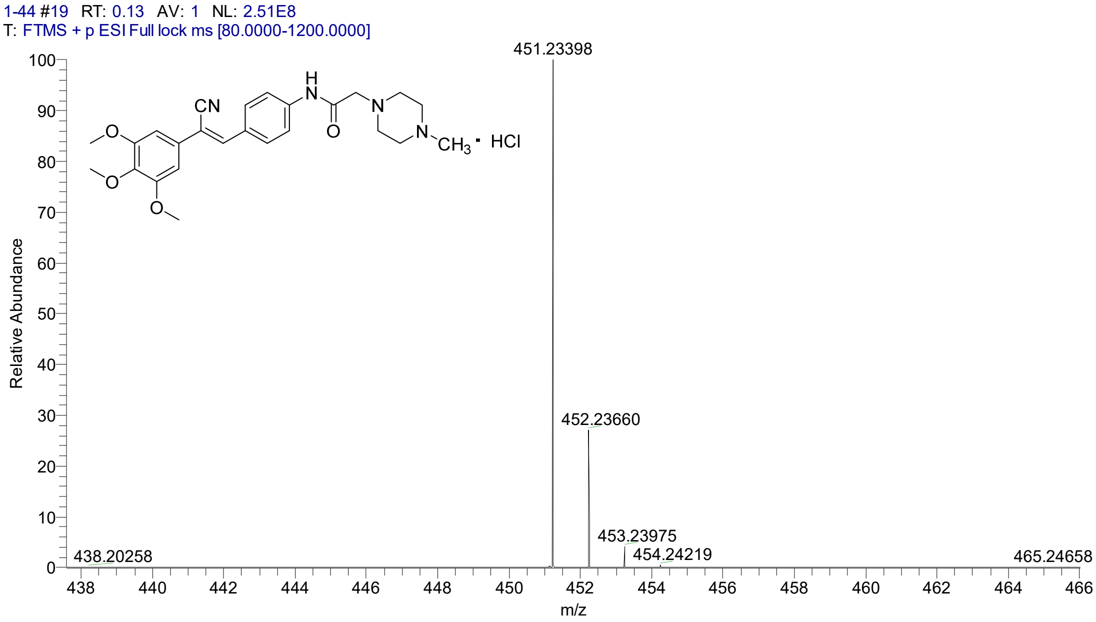

12a2. HRMS (ESI) m/z calcd for $C_{26}H_{33}N_4O_4^+$ $(M+H)^+$ 465.24963, found 465.24979.

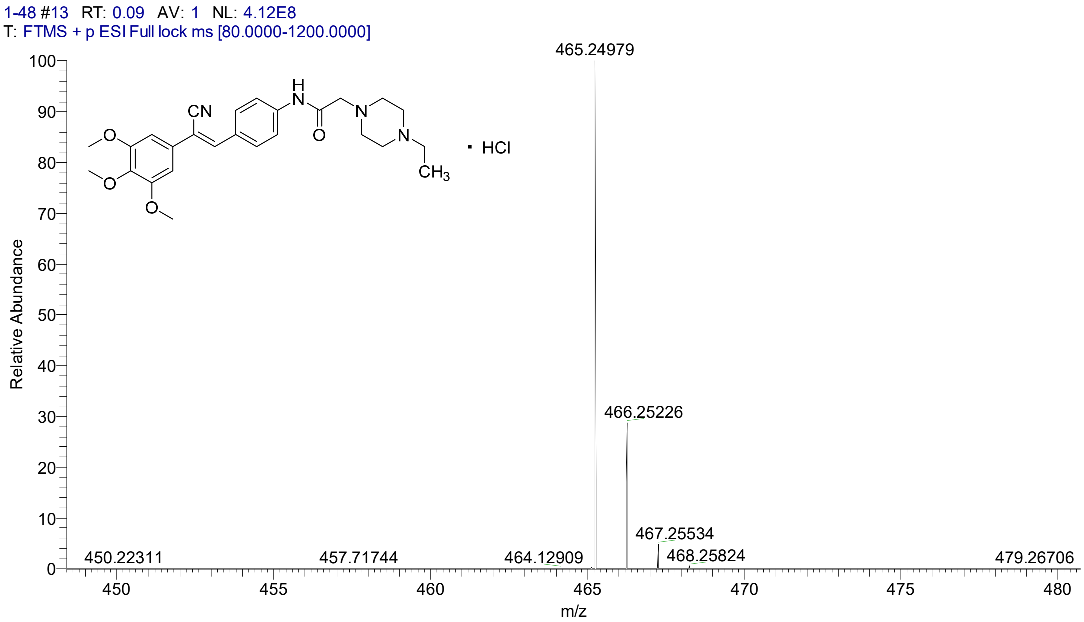

12a3. HRMS (ESI) m/z calcd for $C_{24}H_{28}N_3O_4^+$ $(M+H)^+$ 422.20743, found 422.20746.

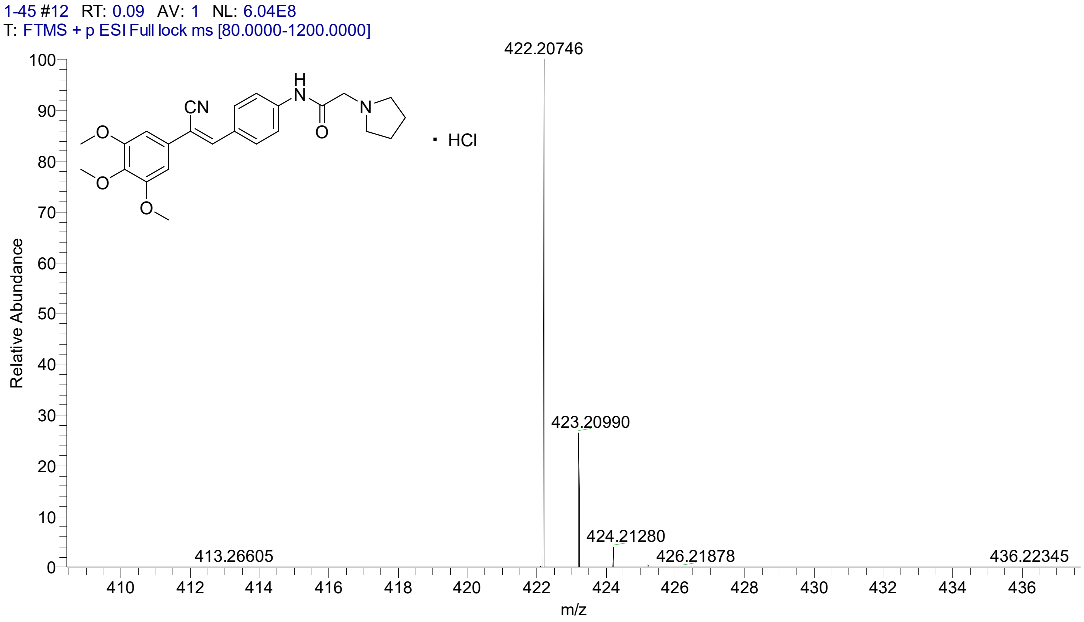

12a$_4$. HRMS (ESI) m/z calcd for $C_{24}H_{30}N_3O_4^+$ $(M+H)^+$ 424.22308, found 424.22314.

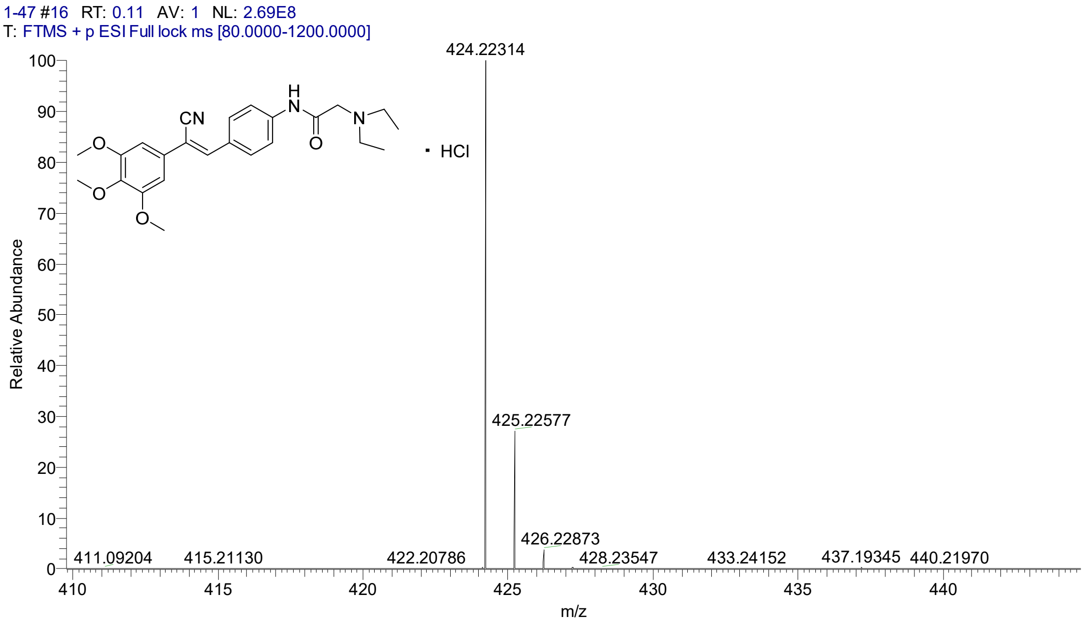

12a$_5$. HRMS (ESI) m/z calcd for $C_{24}H_{28}N_3O_5^+$ $(M+H)^+$ 438.20235, found 438.20209.

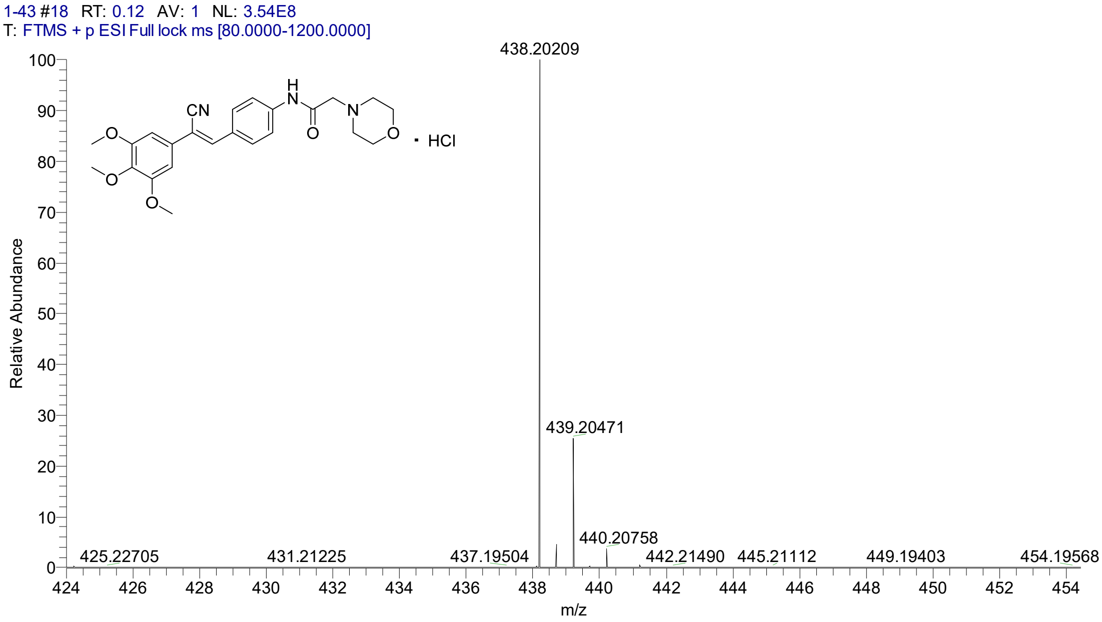

12a$_6$. HRMS (ESI) m/z calcd for $C_{26}H_{32}N_3O_5^+$ (M+H)$^+$ 466.23365, found 466.23389.

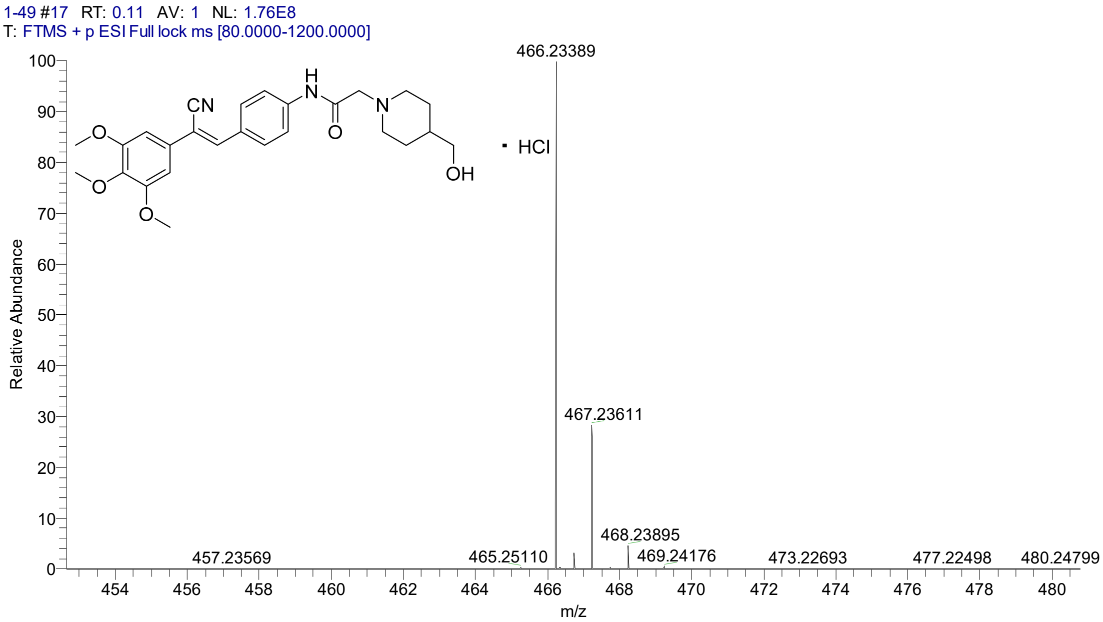

12b$_1$. HRMS (ESI) m/z calcd for $C_{26}H_{33}N_4O_4^+$ (M+H)$^+$ 465.24963, found 465.24985.

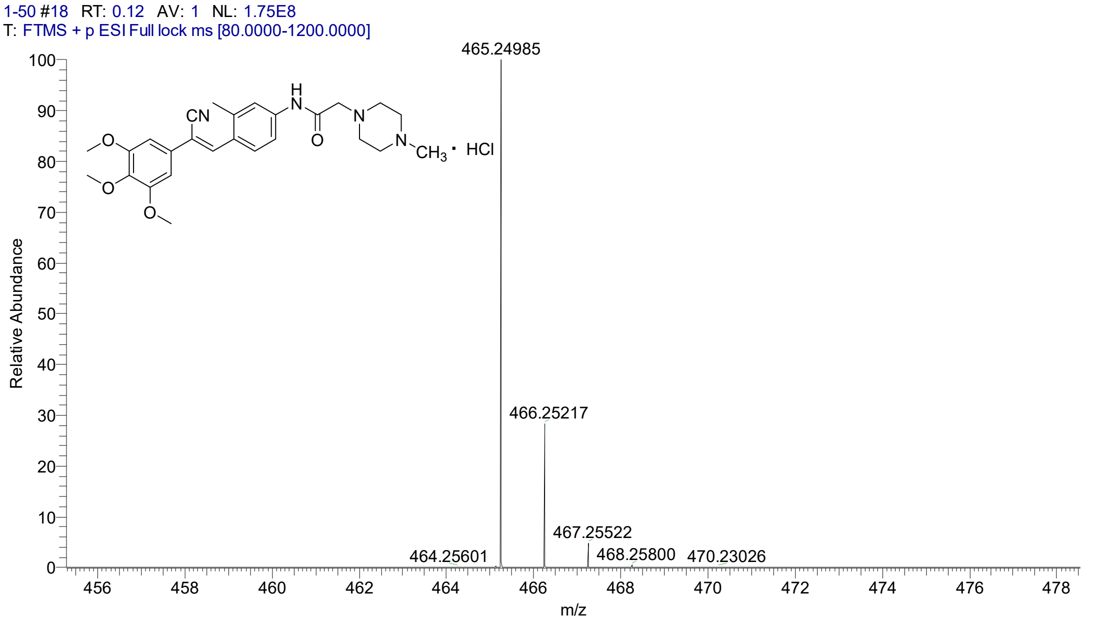

12b₂. HRMS (ESI) m/z calcd for $C_{27}H_{35}N_4O_4^+$ $(M+H)^+$ 479.26528, found 479.26563.

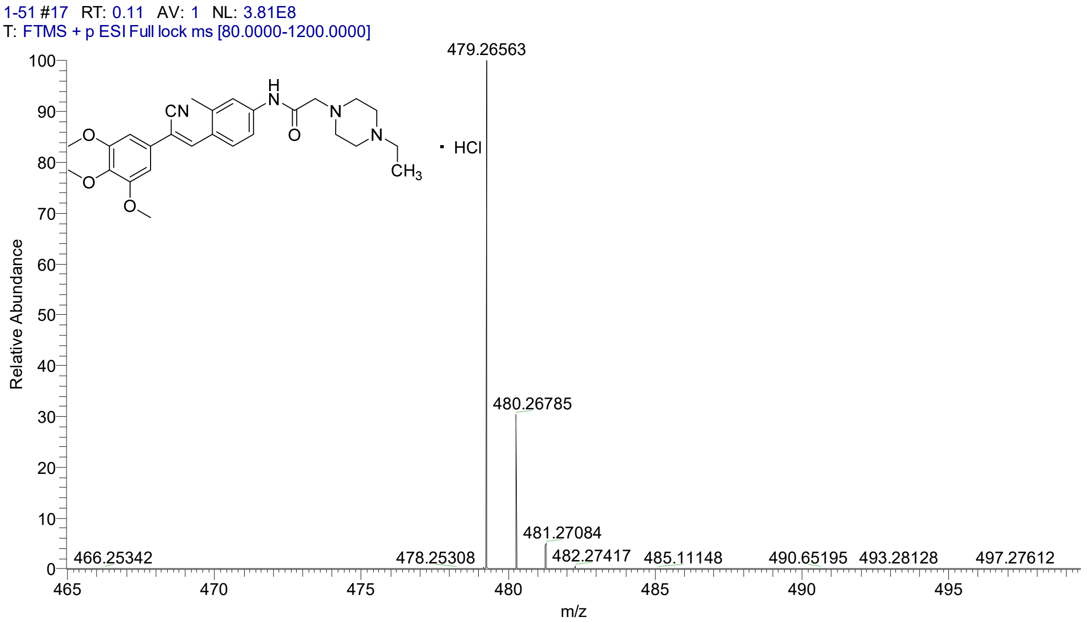

12c₁. HRMS (ESI) m/z calcd for $C_{24}H_{29}N_4O_3^+$ $(M+H)^+$ 421.22342, found 421.22333.

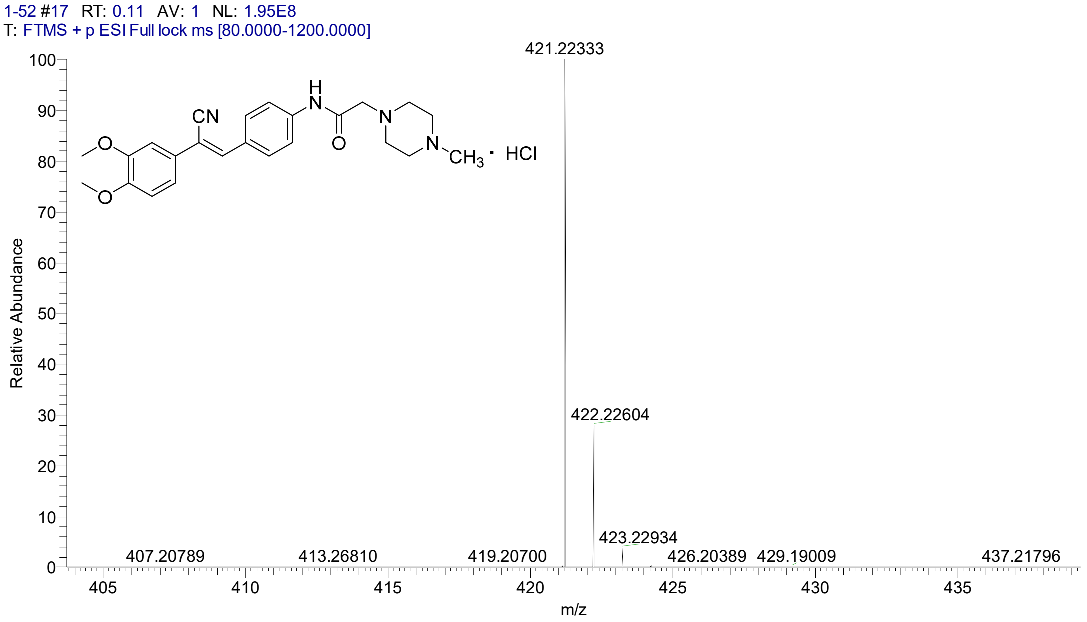

12c₂. HRMS (ESI) m/z calcd for $C_{25}H_{31}N_4O_3^+$ $(M+H)^+$ 435.23907, found 435.23883.

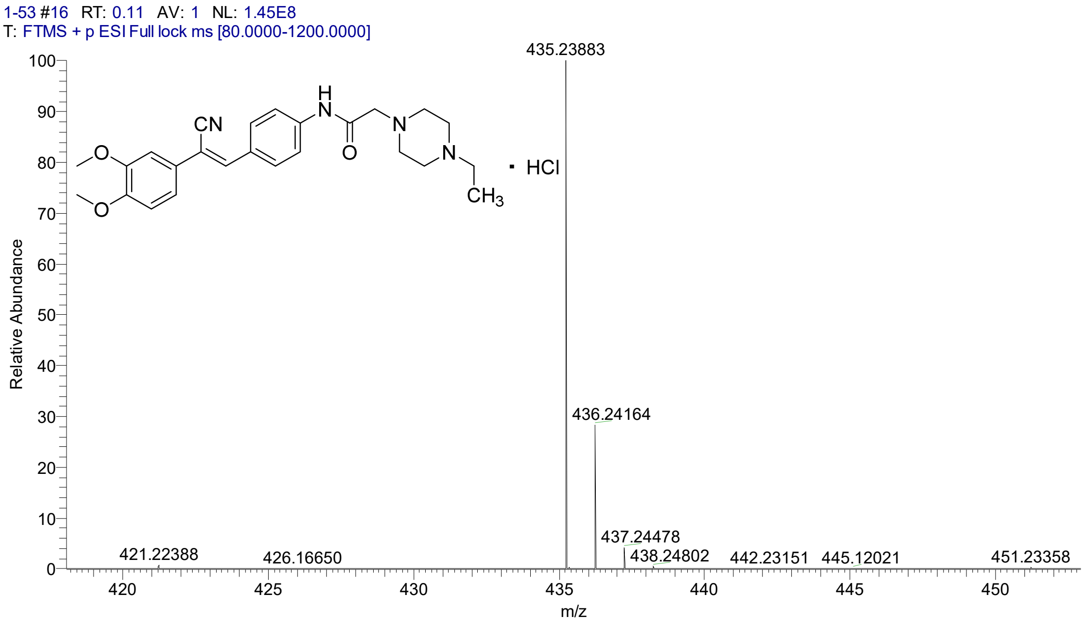

12d₁. HRMS (ESI) m/z calcd for $C_{25}H_{31}N_4O_3^+$ $(M+H)^+$ 435.23907, found 435.23877.

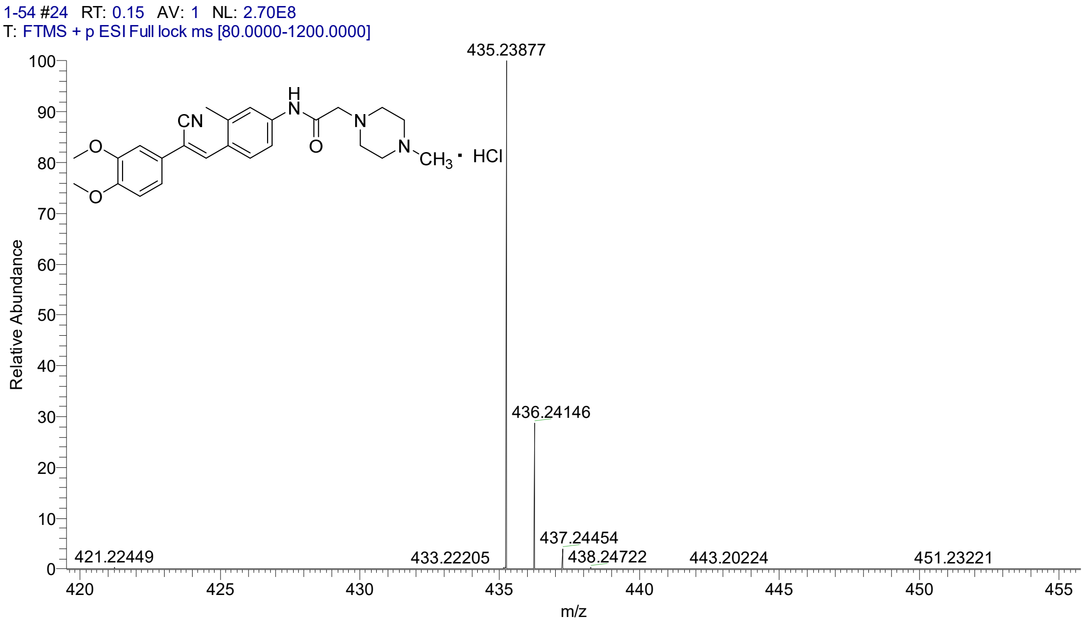

12d$_2$. HRMS (ESI) m/z calcd for $C_{26}H_{33}N_4O_3^+$ $(M+H)^+$ 449.25472, found 449.25452.

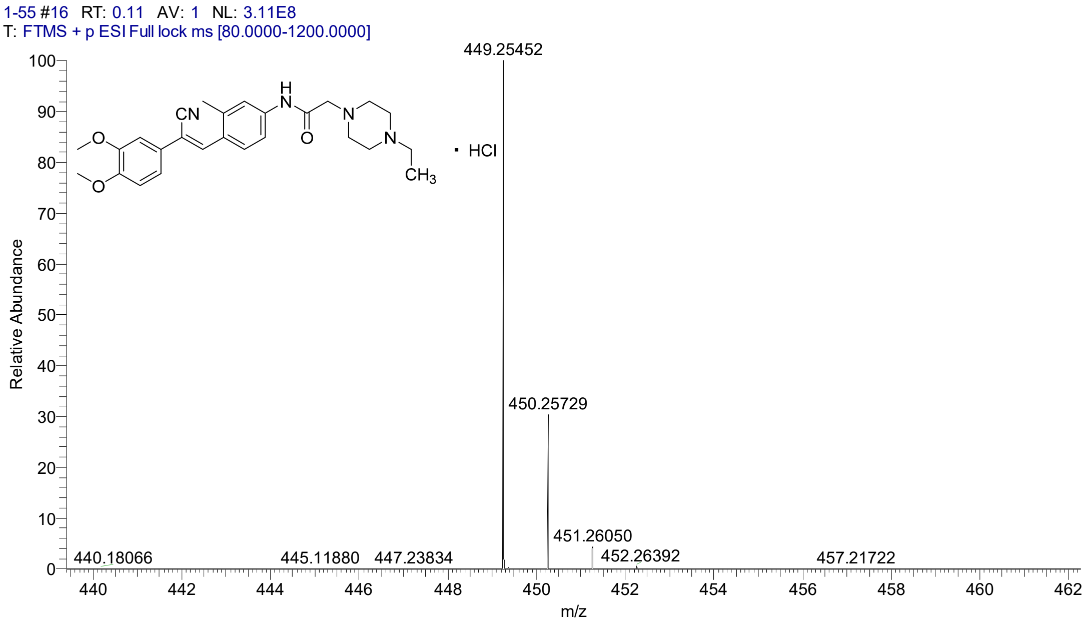

15. HRMS (ESI) m/z calcd for $C_{27}H_{35}N_4O_4^+$ $(M+H)^+$ 479.26528, found 479.26569.

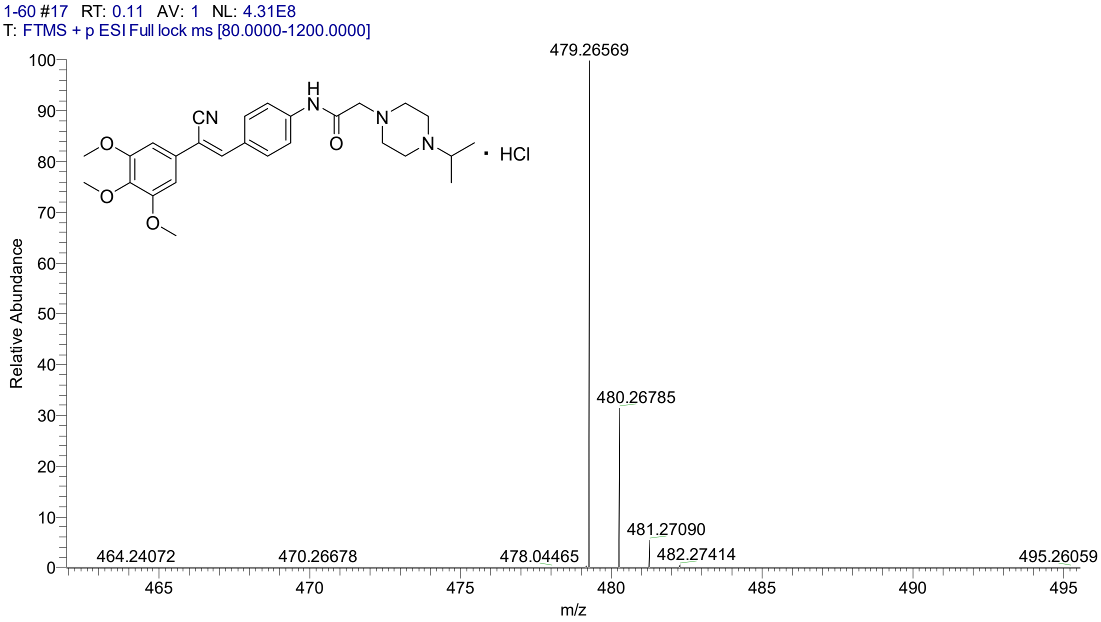

16. HRMS (ESI) m/z calcd for $C_{28}H_{37}N_4O_6^+$ $(M+H)^+$ 525.27076, found 525.27106.

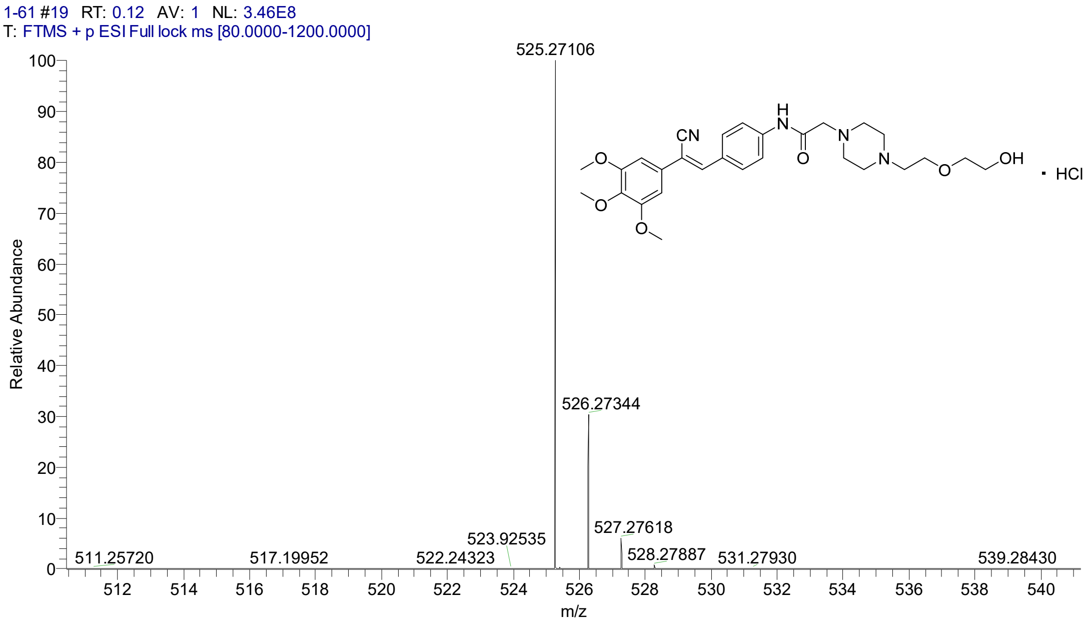

17. HRMS (ESI) m/z calcd for $C_{25}H_{31}N_4O_4^+$ $(M+H)^+$ 451.23398, found 451.23395.

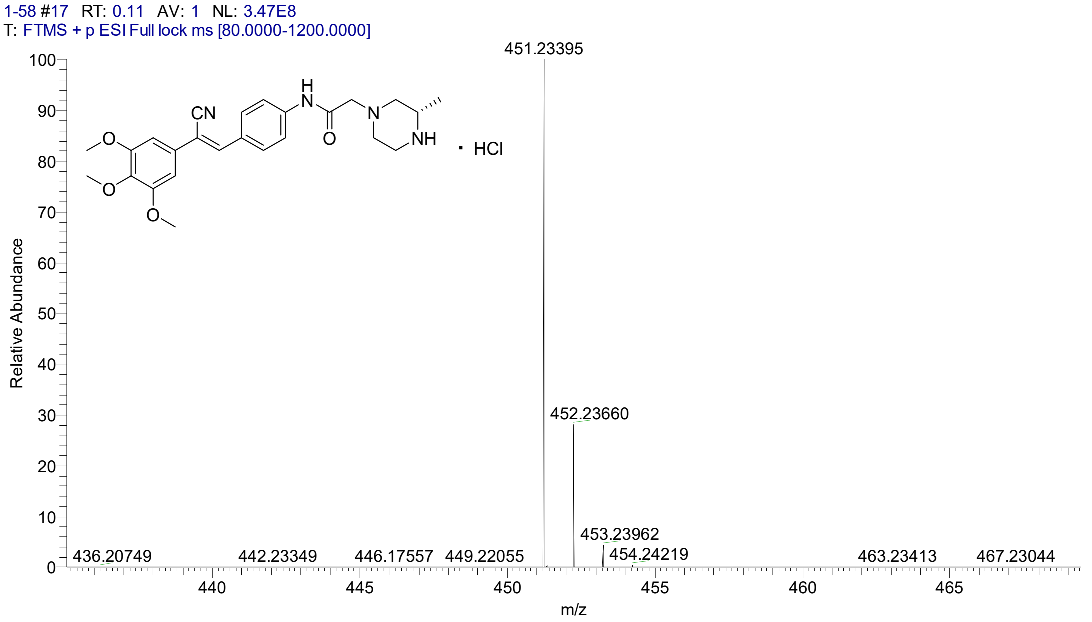

18. HRMS (ESI) m/z calcd for $C_{25}H_{31}N_4O_4^+$ $(M+H)^+$ 451.23398, found 451.23407.

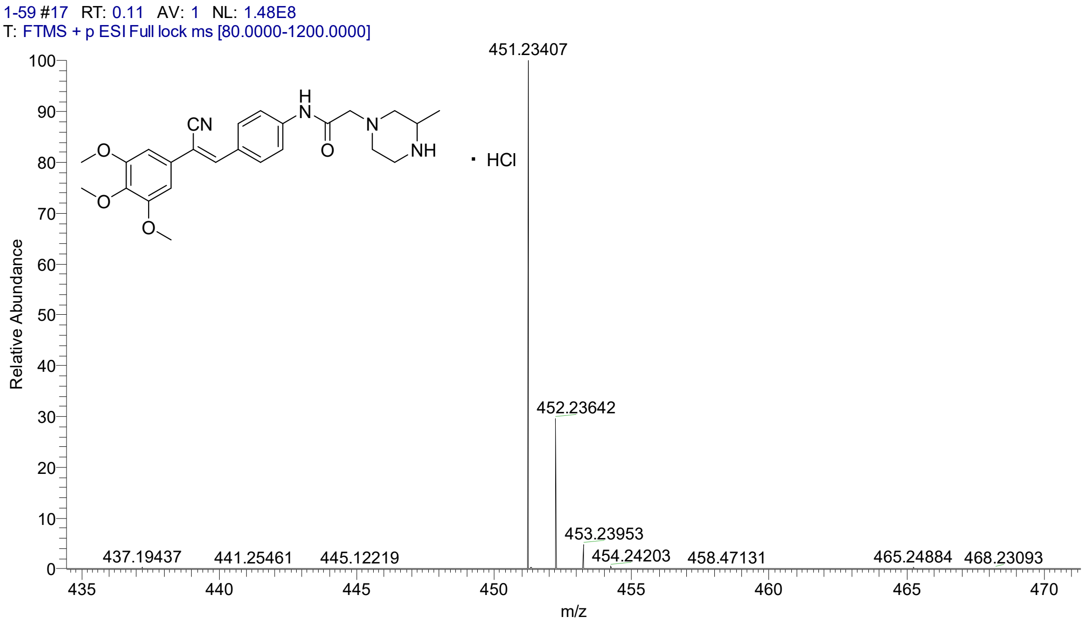

19. HRMS (ESI) m/z calcd for $C_{25}H_{31}N_4O_4^+$ $(M+H)^+$ 451.23398, found 451.23419.

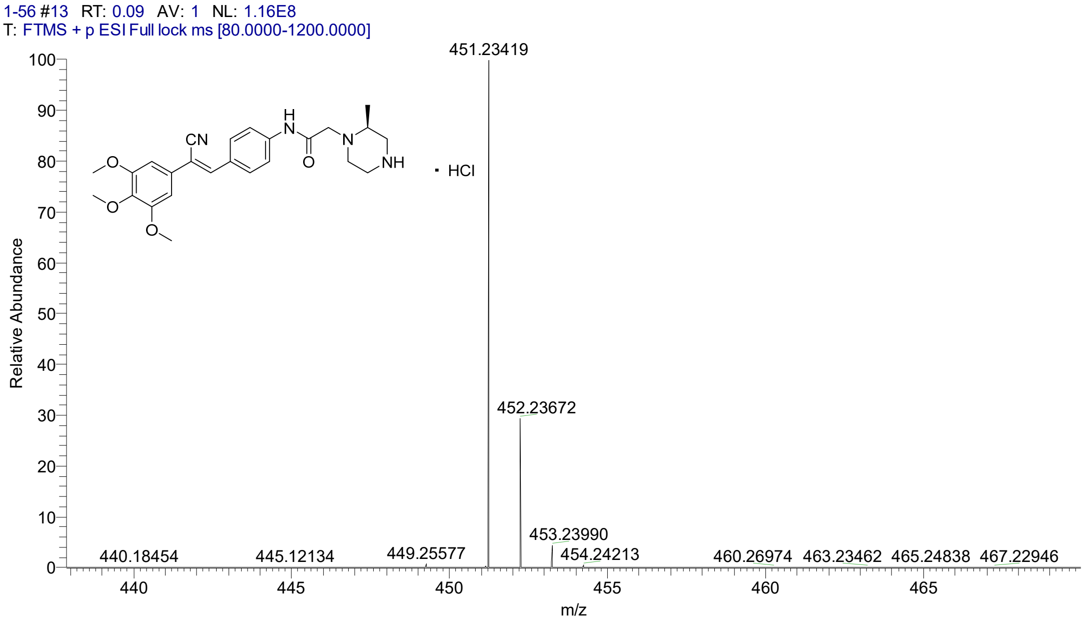

20. HRMS (ESI) m/z calcd for $C_{25}H_{31}N_4O_4^+$ $(M+H)^+$ 451.23398, found 451.23404.

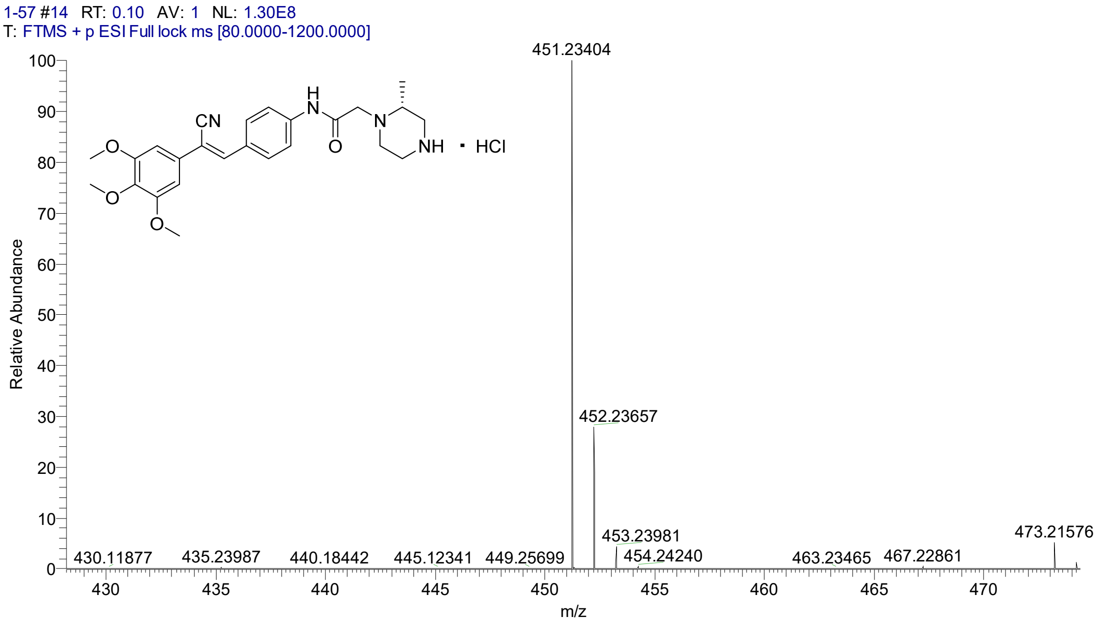

Supplement: Supplementary file 1 [file molecules-28-01717-s001.zip › molecules-2189644-supplementary.pdf]
